# A Novel and Selective Dopamine Transporter Inhibitor, *(S)*-MK-26, Promotes Hippocampal Synaptic Plasticity and Restores Effort-Related Motivational Dysfunctions

**DOI:** 10.3390/biom12070881

**Published:** 2022-06-24

**Authors:** Shima Kouhnavardi, Alev Ecevitoglu, Vladimir Dragačević, Fabrizio Sanna, Edgar Arias-Sandoval, Predrag Kalaba, Michael Kirchhofer, Jana Lubec, Marco Niello, Marion Holy, Martin Zehl, Matthias Pillwein, Judith Wackerlig, Rita Murau, Andrea Mohrmann, Kathryn R. Beard, Harald H. Sitte, Ernst Urban, Claudia Sagheddu, Marco Pistis, Roberto Plasenzotti, John D. Salamone, Thierry Langer, Gert Lubec, Francisco J. Monje

**Affiliations:** 1 Programme for Proteomics, Paracelsus Medical University, 5020 Salzburg, Austria; shima.kouhnavardi@gmail.com (S.K.); predrag.kalaba@univie.ac.at (P.K.); jana.aradska@gmail.com (J.L.); a01347420@unet.univie.ac.at (M.P.); 2 Department of Pharmaceutical Chemistry, Faculty of Life Sciences, University of Vienna, 1090 Vienna, Austria; dragacevicvladimir@gmail.com (V.D.); mikey.k.mb@gmail.com (M.K.); judith.wackerlig@univie.ac.at (J.W.); ernst.urban@univie.ac.at (E.U.); thierry.langer@univie.ac.at (T.L.); 3 Center for Physiology and Pharmacology, Department of Neurophysiology and Neuropharmacology, Medical University of Vienna, 1090 Vienna, Austria; 4 Department of Psychological Sciences, University of Connecticut, Storrs, CT 06269-1020, USA; alev.ecevitoglu@uconn.edu (A.E.); kathryn.beard@uconn.edu (K.R.B.); john.salamone@uconn.edu (J.D.S.); 5 Department of Biomedical Sciences, Division of Neuroscience and Clinical Pharmacology, University of Cagliari, 09100 Cagliari, Italy; fabrizio.sanna@unica.it (F.S.); claudiasagheddu@unica.it (C.S.); mpistis@unica.it (M.P.); 6 Department of Psychobiology, Universitat Jaume I, 12071 Castellón de la Plana, Spain; ariasdes@uji.es; 7 Institute of Pharmacology, Center for Physiology and Pharmacology, Medical University of Vienna, 1090 Vienna, Austria; marco.niello@meduniwien.ac.at (M.N.); marion.holy@meduniwien.ac.at (M.H.); harald.sitte@meduniwien.ac.at (H.H.S.); 8 Department of Analytical Chemistry, Faculty of Chemistry, University of Vienna, 1090 Vienna, Austria; martin.zehl@univie.ac.at; 9 Center for Behavioral Brain Sciences, Institut für Biochemie und Zellbiologie, Otto-von-Guericke-Universität Magdeburg, 39120 Magdeburg, Germany; rita.murau@med.ovgu.de (R.M.); andrea.mohrmann@med.ovgu.de (A.M.); 10 Division of Biomedical Research, Medical University of Vienna, 1090 Vienna, Austria; roberto.plasenzotti@univie.ac.at

**Keywords:** dopamine, dopamine transporter, synaptic plasticity, learning and memory, hippocampus, emotional behavior, effort-related behavior, modafinil

## Abstract

Dopamine (DA), the most abundant human brain catecholaminergic neurotransmitter, modulates key behavioral and neurological processes in young and senescent brains, including motricity, sleep, attention, emotion, learning and memory, and social and reward-seeking behaviors. The DA transporter (DAT) regulates transsynaptic DA levels, influencing all these processes. Compounds targeting DAT (e.g., cocaine and amphetamines) were historically used to shape mood and cognition, but these substances typically lead to severe negative side effects (tolerance, abuse, addiction, and dependence). DA/DAT signaling dysfunctions are associated with neuropsychiatric and progressive brain disorders, including Parkinson’s and Alzheimer diseases, drug addiction and dementia, resulting in devastating personal and familial concerns and high socioeconomic costs worldwide. The development of low-side-effect, new/selective medicaments with reduced abuse-liability and which ameliorate DA/DAT-related dysfunctions is therefore crucial in the fields of medicine and healthcare. Using the rat as experimental animal model, the present work describes the synthesis and pharmacological profile of (*S*)-MK-26, a new modafinil analogue with markedly improved potency and selectivity for DAT over parent drug. Ex vivo electrophysiology revealed significantly augmented hippocampal long-term synaptic potentiation upon acute, intraperitoneally delivered (*S*)-MK-26 treatment, whereas in vivo experiments in the hole-board test showed only lesser effects on reference memory performance in aged rats. However, in effort-related FR5/chow and PROG/chow feeding choice experiments, (*S*)-MK-26 treatment reversed the depression-like behavior induced by the dopamine-depleting drug tetrabenazine (TBZ) and increased the selection of high-effort alternatives. Moreover, in in vivo microdialysis experiments, (*S*)-MK-26 significantly increased extracellular DA levels in the prefrontal cortex and in nucleus accumbens core and shell. These studies highlight (*S*)-MK-26 as a potent enhancer of transsynaptic DA and promoter of synaptic plasticity, with predominant beneficial effects on effort-related behaviors, thus proposing therapeutic potentials for (*S*)-MK-26 in the treatment of low-effort exertion and motivational dysfunctions characteristic of depression and aging-related disorders.

## 1. Introduction

The neurotransmitter DA participates in the modulation of several key functions in humans, influencing processes such as cell proliferation and differentiation during embryologic development, as well as emotional processing, attention, motricity, motivation, and learning and memory in the childhood, adolescence and mature adulthood [1,2,3,4,5,6,7]. Consequently, deficiencies in dopaminergic signaling are associated with several neurodegenerative and incurable conditions such as Alzheimer’s, Parkinson’s, Huntington’s and Lewy body diseases [6,8,9,10,11,12,13,14,15,16,17,18]. Aging, which is accompanied by profound alterations in dopaminergic activity [19,20,21,22], is the predominant naturally occurring high-risk factor for the presence of several maladies associated with dysfunctional dopaminergic signaling, including Parkinson’s and Alzheimer’s diseases, and others such as delirium, depression, dementia and mood-related disorders [23,24,25,26,27,28,29,30]. In particular, disorders associated with DA dysfunctions in the young and aging brains can overlap with profound alterations in motivation-oriented behavior, classically manifested by apathy, a lack of energy, reduced initiation of effort-demanding tasks and a debilitating sense of relinquishment, whose neural bases are thought to involve the nucleus accumbens, hippocampus and prefrontal cortical regions among other neural circuitries [30,31,32]. Unfortunately, there are currently no effective treatments against these motivational disorders and DA-related maladies, and developing novel treatments for dopaminergic deficiencies is therefore crucial in the medical and healthcare fields. We synthetized (*S*)-MK-26, a novel chemical substance with structural analogy to the cognition-enhancing drugs (*R*)-Modafinil [33,34,35], and examined the affinity/selectivity of (*S*)-MK-26 for the dopamine transporter (DAT) by competition binding assays. Our data revealed (*S*)-MK-26 as a highly selective inhibitor of DAT, with a binding efficiency about 130 times superior to that of modafinil. Acute treatment with (*S*)-MK-26 only minimally influenced spatial reference memory in aged rats, but acted as a booster of synaptic plasticity in the hippocampus and as a potent enhancer of transsynaptic dopamine in the prefrontal cortex and nucleus accumbens, all areas critical for motivational, effort-exertion, and learning and memory-related functions [32,36,37,38]. All these observations suggested potential applications for (*S*)-MK-26 treatment for effort-related motivational dysfunctions. Thus, (*S*)-MK-26 was assessed using two different lever pressing tests of effort-based choice, the fixed ratio (FR) 5/chow feeding choice task and the progressive ratio (PROG)/chow feeding choice task. For the FR5/choice studies, (*S*)-MK-26 was tested across both low and high dose ranges for its ability to reverse the low-effort bias (i.e., reduced choice of high-effort lever pressing) induced by the DA storage inhibitor tetrabenazine (TBZ). TBZ is an inhibitor of the vesicular monoamine transporter type-2 and was used in these studies because it induces depressive-like symptoms in humans [31]. The PROG/chow feeding choice procedure was employed to determine if (*S*)-MK-26 could increase the selection of high-effort PROG lever pressing when administered alone. These behavioral pharmacology procedures have been used previously to assess the effects of modafinil and its analogs that act as DAT inhibitors [31,39,40]. These experiments showed that (*S*)-MK-26 has the ability to reverse pharmacologically induced motivational dysfunctions and enhance the selection of the high-effort alternatives. Given the importance of the dopaminergic molecular machinery in young and mature-adult brains in health and disease, the identification and characterization of (*S*)-MK-26 is of timely relevance due to its potential applications in both basic-experimental and applied clinical research.

## 2. Chemistry

**Figure 1:**
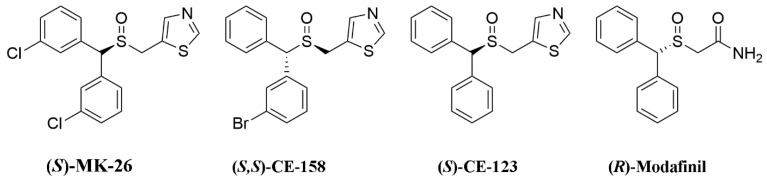
Structures of (*S*)-MK-26; (*S*,*S*)-CE-158; (*S*)-CE-123; and (*R*)-Modafinil. The (*S*)-MK-26 molecule has only one chiral center (having thus only two optical enantiomeric, “R” and “S” stereoisomers). (*S*)-MK-26 was initially generated as a racemic (an equal mixture of enantiomers) compound and afterwards separated by means of chiral-phase discrimination using HPLC chromatography. This physical separation method first yielded a less retained enantiomer ((*S*)-MK-26), and a second, stronger retained enantiomer ((*R*)-MK-26; “9” in Scheme 1).

(*S*)-MK-26, synthetized following previously described protocols [41], has structural similarities to the very well-characterized nootropic modafinil [42], with the carboxamide replaced with thiazole (five-membered heterocycle attached through position 5) and with a chlorine atom added in the *meta* position on both phenyl rings, thus also being a close structural analogue of the previously described compounds (*S*,*S*)-CE-158 and (*S*)-CE-123 [39,41,42,43] (Figure 1).

**Scheme 1:**
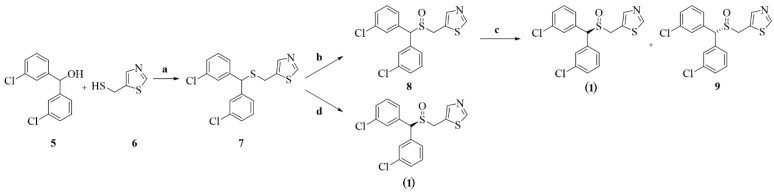
Synthetic routes for (*S*)-MK-26 ((1)*^a^*). *^a^* Reagents and conditions: (a) BF_3_•Et_2_O, CH_3_COOH, r.t. overnight; (b) CH_3_COOH, H_2_O_2_, r.t. overnight; (c) chiral separations, Chiralpack IA column, isocratic 100% EtOAc as mobile phase (d) I. (+)-diisopropyl L-tartrate, titanium (IV) isopropoxide, acetone, H_2_O, reflux 1 h, II. DIPEA, cumene hydroperoxide, acetone, r.t. 3 h.

Following their chiral resolution, as outlined above, the separated enantiomers (*S*)-MK-26 and (*R*)-MK-26 (“9” in Scheme 1) were given their respective absolute configurations. This work was outsourced to BioTools Inc. (Jupiter, Florida, FL, USA). The absolute configuration of single enantiomers was determined via a comparison of their respective measured and predicted VCD spectra. For details, please refer to Supplementary Materials. The analysis attributed (*S*) the absolute configuration to the less retained (*S*)-MK-26 and (*R*) absolute configuration to the more retained enantiomer (*R*)-MK-26.

## 3. Results

### 3.1. (S)-MK-26 Is a Potent and Selective DAT Inhibitor

**Figure 2:**
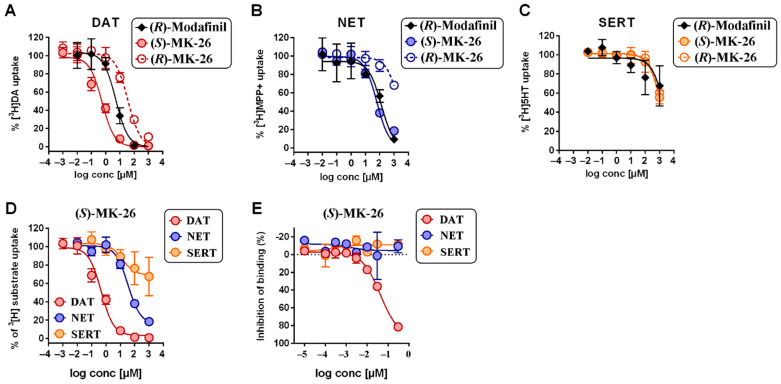
Uptake inhibition and radioligand binding assays. (**A**) Uptake inhibition of (*S*)-MK-26, (*R*)-Modafinil, and (*R*)-MK-26 at DAT (red); (**B**) NET (blue); (**C**) SERT (yellow) stably expressed in HEK293 cells. Uptake substrates used were 0.15 μM [^3^H]DA, 0.05 μM [^3^H]MPP+, and 0.1 μM [^3^H]5-HT, respectively. (**D**) Selectivity profile of (*S*)-MK-26 at DAT, NET and SERT in the substrate uptake-inhibition assay. Data are mean ± SD. (**E**) Binding affinity of (*S*)-MK-26 on DAT, NET, and SERT expressed in CHO cells. Effects were determined by specific antagonist radioligand assays. IC_50_ values and details in the main text. IC_50_ values are derived from non-linear regression using the equation Y = Bottom + (Top − Bottom)/(1 + 10^((X − LogIC_50_))).

In vitro re-uptake inhibition assays on HEK293 cells expressing hDAT, hNET or hSERT were used to obtain preliminary pharmacological information on MK-26 enantiomers (*S*) and (*R*) in comparison to (*R*)-Modafinil. At hDAT, (*S*)-MK-26 showed an IC_50_ value of 0.56 µM (95%CI: 0.41–0.76), which is a 10-fold lower IC_50_ value compared to the parent (*R*)-Modafinil (IC_50_ = 5.14 µM; 95%CI: 3.62–7.31), and approximately 65-fold lower compared to (*R*)-MK-26 (IC_50_ = 36.25 µM; 95%CI: 27.56–47.69). Both enantiomers, (*S*)-MK-26 and (*R*)-MK-26, exhibited low affinity to hNET with IC_50_ = 63.51 µM (95%CI: 48.76–82.72) and 2161 µM (95%CI: 1679–2781), respectively. These values are similar to (*R*)-Modafinil, showing an IC_50_ = 127 µM (95%CI: 68.29–236.9). Moreover, both (*S*)-MK-26 and (*R*)-MK-26 did not show any pharmacological relevant interaction with hSERT (IC_50_ > 1 mM). Taken together, these data establish (*S*)-MK-26 as a dopamine reuptake inhibitor superior to modafinil both in potency to hDAT and in selectivity to other catecholamine transporters (Figure 2). These results are in line with the binding affinities, where (*S*)-MK-26 demonstrated a high binding affinity to DAT (Ki = 26 nM) and trifling bindings to NET and SERT (Figure 2).

### 3.2. Pharmacokinetic Profiling of (S)-MK-26

**Figure 3:**
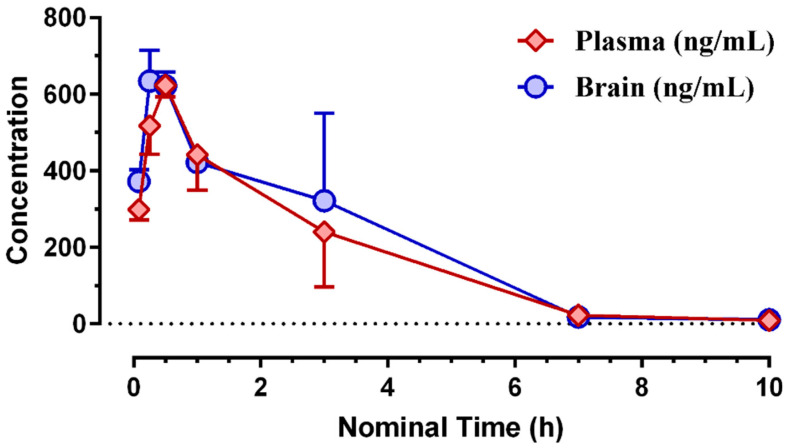
Brain (**blue**) and plasma (**red**) concentrations of (*S*)-MK-26 upon i.p. administration with dose of 10 mg/kg of body weight.

Pharmacokinetic parameters were determined after i.p. administration of (*S*)-MK-26 at a dosage of 10 mg/kg of body weight. The mean plasma concentrations peaked within 30 min after i.p. administration (C_max_ = 624 ± 51 ng/mL). Mean brain concentrations reached a maximum of 634 ± 140 ng/g at 15 min and the mean brain to plasma concentration ratio value was 1.12 ± 0.19. Pharmacokinetic parameters are summarized in Table 1. (*S*)-MK-26 was detectable up to 10 h in the brain and plasma. The plasma and brain concentrations of (*S*)-MK-26 at different time points are illustrated in Figure 3. These findings suggest that i.p. administration of 10 mg/kg body weight should be sufficient to achieve brain concentrations above the level necessary for DAT inhibition.

### 3.3. (S)-MK-26 Increases Extracellular Dopamine Levels In Vivo

**Figure 4:**
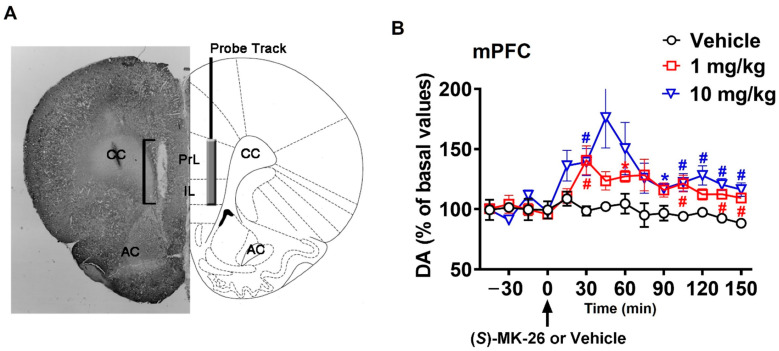
Treatment with (*S*)-MK-26 enhanced brain extracellular concentration of dopamine in vivo. (**A**) Schematic representation of a coronal section of the rat brain (adapted from Paxinos and Watson, 2014 [45]) showing the track of the microdialysis probe in the PrL and IL portions of the mPFC. The square bracket in the microphotograph indicates the portion of the histological section showing the active part of the dialyzing membrane of the microdialysis probe. (**B**) Extracellular dopamine concentrations in the mPFC dialysates obtained from rats treated with (*S*)-MK-26 (1 and 10 mg/kg) or vehicle. Rats stereotaxically implanted with a microdialysis probe aimed at the mPFC were placed individually into the experimental cage and perfused with the dialysis buffer. During the experiment, four dialysate aliquots were collected every 15 min and then vehicle or (*S*)-MK-26 (1 or 10 mg/kg) was i.p. administered (indicated by the arrow in the figure) and 10 additional dialysate aliquots were also collected for dopamine determination. Data are charted as percentages (with 100% as the average of the last four dopamine basal values before treatment) with values expressed as means ± SEM of the measurements obtained by 3–5 rats per group. *: *p* < 0.05 compared to basal values (before treatment); #: *p* < 0.05 with respect to vehicle-treated rats (two-way RM-ANOVA followed by Tukey’s multiple-comparison test). Abbreviations: PrL, prelimbic area; IL, infralimbic area; AC, anterior commissura; CC, corpus callosum.

In order to examine the effect of (*S*)-MK-26 on the dopamine system in vivo, we measured extracellular dopamine levels in the nucleus accumbens (NAc) core and shell, and medial prefrontal cortex (mPFC), both of which are VTA-innervated brain regions involved in learning and memory [44]. Basal dopamine levels in the mPFC (Figure 4) were 2.20 pg in 20 µL of dialysate, corresponding to an extracellular dopamine concentration of ≅0.72 nM. The systemic administration (i.p.) of (*S*)-MK-26 augmented dopamine concentrations in a dose-dependent manner, while no enhancing effect was observed after the treatment with vehicle. A two-way ANOVA revealed significant effects of Time [F_(4.3,43.4)_ = 4.161, *p* < 0.005], Treatment [F_(2,10)_ = 28.99, *p* < 0.0001] and a significant Time x Treatment interaction [F_(26,130)_ = 1.674, *p* < 0.032]. Subsequent Tukey’s post hoc comparisons revealed that (*S*)-MK-26, at the dose of 10 mg/kg, significantly increased dopamine concentrations at 90 min after the treatment when compared to basal values and from 30 to 150 min when compared to vehicle, with peak values at 45 min after the treatment (by about 75%). A similar trend was observed at 1 mg/kg, with peak values at 30 min after the treatment (by about 40%; Figure 4).

**Figure 5:**
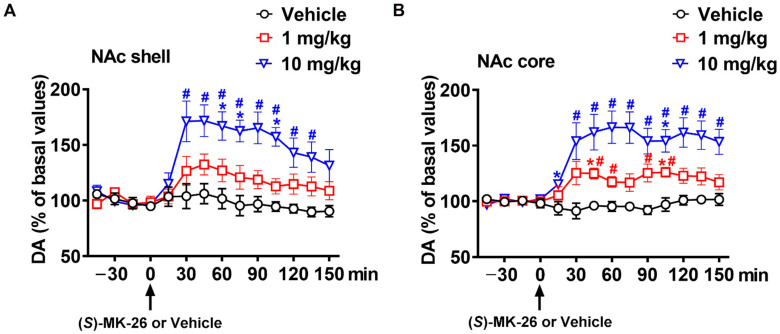
Extracellular dopamine concentrations in the accumbal shell (**A**) and core (**B**) dialysates obtained from SD male rats treated with (*S*)-MK-26 (1, 10 mg/kg) or vehicle (Kolliphor EL 30%). Rats stereotaxically implanted with a microdialysis probe aimed at the accumbal shell or core were placed individually into the experimental cage and perfused with dialysis buffer. During the experiment, four initial dialysate aliquots were collected, one every 15 min, before intraperitoneal administration (indicated by arrows) of either the vehicle (Kolliphor EL 30% 1.5 mL/kg) or (*S*)-MK-26 (1 or 10 mg/kg) and collection of subsequent 10 dialysate aliquots for dopamine determination commenced. Values are percentages (with 100% as the average of the last four dopamine basal values before treatment) and expressed as means ± SEM of the values obtained by 3–5 rats per group. *: *p* < 0.05 with respect to basal values (before treatment); #: *p* < 0.05 with respect to vehicle-treated rats (two-way ANOVA for repeated measures followed by Tukey’s multicomparison test). Basal dopamine levels in NAc were 6.53 pg (in shell) and 7.29 pg (in core) per 20 µL of dialysate, corresponding to extracellular dopamine concentrations of approximately 2.13 nM and 2.38 nM, in shell and core, respectively.

The systemic (i.p.) treatment with (*S*)-MK-26 dose-dependently increased the dopamine levels in both the NAc shell (Figure 5A) and NAc core (Figure 5B), while no effect was observed after the treatment with vehicle. The two-way ANOVA revealed significant effects of time (F_(3.351,33.51)_ = 9.257, *p* < 0.0001; F_(2.710,27.10)_ = 11.65, *p* < 0.0001; for the NAc shell and NAc core, respectively), Treatment (F_(2,10)_ = 8.595, *p* < 0.007 for the NAc shell; F_(2,10)_ = 10.10, *p* < 0.004 for the NAc core) and a significant Time × Treatment interaction (F_(26,130)_ = 3.806, *p* < 0.0001; F_(26,130)_ = 5.65, *p* < 0.0001 for the NAc shell and NAc core, respectively) in both subareas of the NAc. Tukey’s post hoc comparisons revealed that in the NAc shell (*S*)-MK-26 at 10 mg/kg body weight dose significantly increased dopamine concentrations between 60 and 120 min after the treatment compared to basal values and between 30 and 135 min when compared to vehicle, with peak values 30–45 min after the treatment (by about 70%) (see Figure 5A for individual points of statistical significance). Similarly, Tukey’s post hoc comparisons revealed that in the NAc core (*S*)-MK-26 at 10 mg/kg body weight dosage significantly increased dopamine concentrations from 15 to 105 min after the treatment compared to basal values and from 30 to 150 min when compared to vehicle, with peak values 60–75 min after the treatment (by about 65%). The lower dose of (*S*)-MK-26 (1 mg/kg) was sufficient to significantly increase dopamine concentration in the NAc core (not shell), with a peak action between 30–45 and 60–120 min after the treatment when compared to basal values and/or vehicle treatment, with peaks of about 25% above basal values (see Figure 5B for individual points of statistical significance).

The above-reported differences were confirmed by two-way ANOVA analysis of areas under the curves (AUCs), that revealed a significant effect of Treatment (F_(2,30)_ = 26.91, *p* < 0.0001), but not of Area (F_(2,30)_ = 0.6768, *p* > 0.05) nor a significant Treatment*Area interaction (F_(4,30)_ = 0.5792, *p* > 0.05) (see also Table 2).

### 3.4. (S)-MK-26 Has a Transient Effect on Spontaneous Locomotion and Exploration

**Figure 6:**
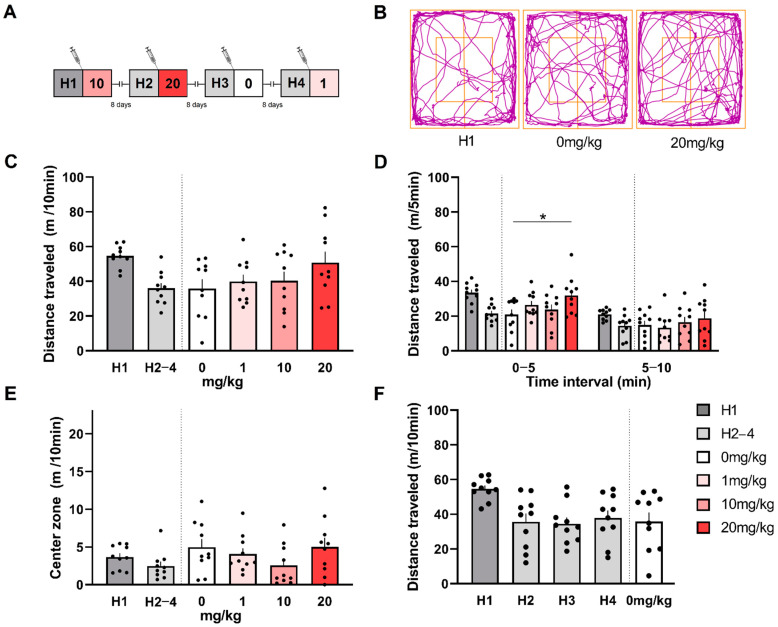
Effect of (*S*)-MK-26 (1, 10, 20 mg/kg body weight) on locomotor activity in an open-field apparatus. (**A**) Schematic demonstration of experimental design. (**B**) Representative traces of locomotor activity recorded during H1 session and test session with vehicle and 20 mg/kg treatment with (*S*)-MK-26. (**C**) Total distance travelled over the 10 min test session and (**D**) over 5 min segments. (**E**) Distance travelled in center zone. (**F**) Baseline activity measured during habituation sessions (H1—novelty OF session; H2–4—familiar OF sessions) and vehicle session (0 mg/kg). One-way RM-ANOVA followed by Dunnett post hoc test for multiple comparisons; * *p* < 0.05. Values are presented as mean ± SEM; n = 10.

The effect of (*S*)-MK-26 on spontaneous locomotor activity in naive young (12 weeks) male rats was evaluated in the open-field apparatus. (*S*)-MK-26 had no effect on total distance travelled over 10 min (F_(3,17)_ = 1.710; *p* = 0.1886, one-way ANONA) as compared to vehicle (Figure 6C). Time segment analysis of distance travelled showed that (*S*)-MK-26 at highest dose moderately increased activity within the initial 5 min of the test session (*p* = 0.0236; Dunnett test following ANOVA (F_(3,27)_ = 2.899; *p* = 0.0533)) (Figure 6D). The drug-free sessions (habituation H2 to H4) were interpolated to the study to control a possible cumulative effect of the compound. Baseline activity measured during habituation sessions (one day prior to the test) revealed no significant difference compared to vehicle (*p* > 0.05) (Figure 6F). The drug did not induce stereotypic or anxiogenic behavior (Figure 6B,E).

### 3.5. (S)-MK-26 Has Lesser Effects on Reference Memory

**Figure 7:**
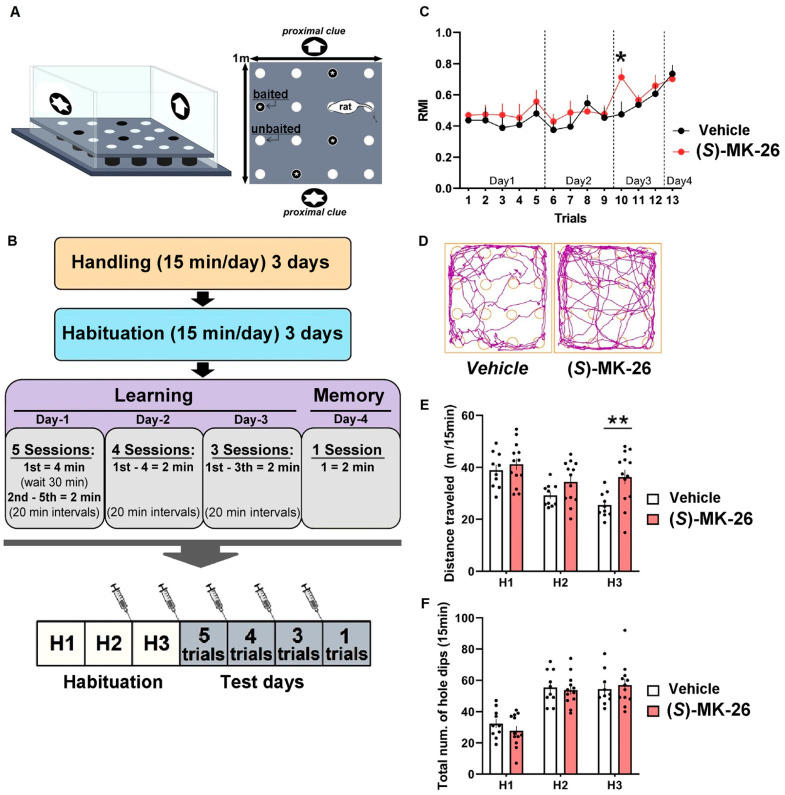
(*S*)-MK-26 has only lesser effects on reference memory. (**A**) Schematic of the hole-board maze, a paradigm for the evaluation of spatial learning and memory (left) with depictions (right) of dimensions (an animal at approximated size scale relative to the size of the hole-board arena is schematized); localization of proximal clues; spatial distribution of the holes; and representation of baited and unbaited holes (see also Materials and Methods). (**B**) Flow diagram representing the methodological strategy used during the preparation and implementation of the hole-board test. (**C**) Performance of aged rats (19 months old) in spatial memory task over thirteen trials (over 4 days) in the (*S*)-MK-26-treated and control vehicle-treated control groups showed significance though frail enhancement of reference memory induced by (*S*)-MK-26 (*p* = 0.0202) detected by Sidak’s test following repeated-measures ANOVA). (**D**) Representative traces of locomotor activity recorded during the H3 session in each treatment group. (**E**) (*S*)-MK-26 increased distance travelled in 15 min habituation session (*p* = 0.0053, unpaired *t* test) (H1, H2—drug free; H3—first treatment). (**F**) Total number of hole dips in the 15 min habituation sessions. Values are presented as mean ± SEM; *n* = 10–12; * *p* < 0.05, ** *p* < 0.01.

We next examined the effects of (*S*)-MK-26 on cognitive performance in the hole-board test, which is a standardized behavioral test used to assess different aspects of learning and memory, including the impact of aging on reference memory, in mice and rats [46,47,48]. To this aim, we randomly assigned some of the aging animals to the (*S*)-MK-26 treatment (*n* = 13; i.p. injected with 10 mg/kg of (*S*)-MK-26 in Kolliphor) and others to the control treatment (*n* = 12; i.p. injected with a weight/equivalent volume of Kolliphor) groups, and examined the acute responses to the treatments in the hole-board maze in our experimental conditions (Figure 7A,B; see also Section 6 (Materials and Methods)). Animals received the first injection 30 min prior to the third habituation (H3) session. (*S*)-MK-26, however, resulted in only a slight and transitory—yet statistically significant—enhancement of spatial reference memory (Figure 7C). (*S*)-MK-26 also induced an increase in ecology-like exploratory activity (Figure 7E) in the hole-board arena during the H3 session (*p* = 0.0053, unpaired *t*-test) (Figure 7D,E). We assumed ecology-like exploratory activity in the hole-board tests on the basis of the absence of exacerbated locomotor activity during examinations in the open field when (*S*)-MK-26 was used at the 10 mg/kg concertation. No differences in the number of hole dips were found between the two groups (Figure 7F). Given that in our hole-board assay the animals experienced several consecutive days of (*S*)-MK-26 presentation, this could have generated a cumulative tolerance-like effect. We next sought to conduct additional experiments aiming to exploring—in the very same animals—the effects of only one single acute exposure to (*S*)-MK-26. To this aim, we first let all these animals rest for at least one month without exposure to the (*S*)-MK-26 (a window of time even larger than the one given as drug-free period in the open-field experiments). Subsequently, we explored the effects of a single acute administration of (*S*)-MK-26, on the same animals, on hippocampal synaptic circuits, which are known to be important for memory storage, by using ex vivo electrophysiology (see below).

### 3.6. (S)-MK-26 Enhanced Synaptic Plasticity at CA3-CA1 Synapses of Aged Rats

**Figure 8:**
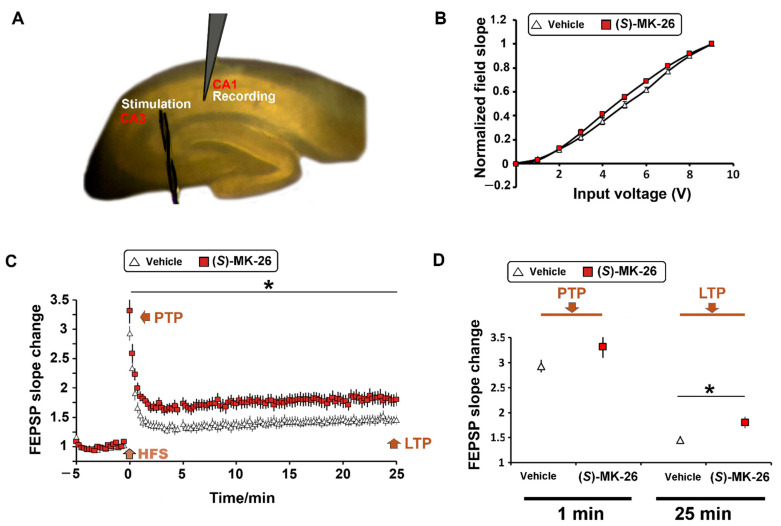
Effect of (*S*)-MK-26 on synaptic functions in the rat hippocampus as examined electro-physiologically in slices. (**A**) Illustrative setting of a rat hippocampal slice with positioned bipolar stimulation and recording electrodes located at the CA3 and CA1 synaptic regions, respectively. (**B**) Analysis of basal synaptic transmission (input/output curves) from recordings obtained in slices derived from animals treated with (*S*)-MK-26 or vehicle. No statistically significant differences were observed between experimental groups (two-way repeated-measures ANOVA; F_(1,22)_ = 0.2406; *p* = 0.6286; *n* = 12 animals per group). (**C**) Averaged values of the fEPSP slopes through time, in slices obtained from animals that underwent (*S*)-MK-26 and Koliphor/vehicle treatments. The time of delivery of the high-frequency stimulation (HFS) protocol, as well as Post-Tetanic Potentiation (PTP) and Long-Term Potentiation (LTP), are indicated by wide-pointing filled arrows (see also Materials and Methods). Whereas no differences between groups were apparent during PTP (which was markedly promoted in both cases), slices derived from animals treated with (*S*)-MK-26 presented with a marked increase in LTP compared to controls (repeated-measures ANOVA F _(1,22)_ = 7.258, *p* = 0.0133, *n* = 12/group). (**D**) Histogram for the field slope values specific to min 1 and 25 after LTP induction. * = *p* < 0.05. Data are displayed as mean ± SEM.

The hippocampus, a structure critical for learning and memory functions [49], is highly regulated by dopaminergic signaling [50,51,52,53]. Given the selective potency of (*S*)-MK-26 to inhibit DAT, and as dopaminergic signaling is critical for the modulation of hippocampal synaptic plasticity [50,51,52], we sought to examine the effects of (*S*)-MK-26 on synaptic transmission and plasticity in CA3-CA1 hippocampal synapses using ex vivo slice electrophysiology (Figure 8A) as previously described [54,55,56,57]. For this, old rats (~20 months old) were injected (i.p.) with 10 mg/kg body weight of (*S*)-MK-26 dissolved in Koliphor (BASF Corp., 30% in distilled water). Slices were prepared 1 h after (*S*)-MK-26 injections and left to recover for 1 h before electrophysiological measurements. We first obtained I/O curves (Materials and Methods) in order to examine the impact of (*S*)-MK-26 on basal synaptic transmission. Our analyses of field excitatory post-synaptic potential (fEPSPs) slopes vs. voltage stimulation intensities showed no effects of (*S*)-MK-26 on basal synaptic transmission, when compared to Koliphor/vehicle control group (Figure 8B). These observations indicate that (*S*)-MK-26, at the concentrations and administration conditions used here, does not alter hippocampal synaptic transmission.

Long-term potentiation (LTP), a proposed neuronal synaptic mechanism for memory [58], has been shown to occur in vivo during hippocampus-dependent learning and memory functions [59]. We, therefore, next examined the effects of (*S*)-MK-26 on LTP (Materials and Methods). Recordings in slices from animals treated with 10 mg/kg of (*S*)-MK-26 exhibited Post-Tetanic Potentiation (PTP, examined 1 min after LTP-induction; see also [60]) comparable to those from the Koliphor/control group but presented with a significant enhancement in their LTP responses (examined 25 min after LTP induction; Figure 8C,D).

### 3.7. (S)-MK-26 Restores Motivational Impairments in Effort-Based Choice Tasks

**Figure 9:**
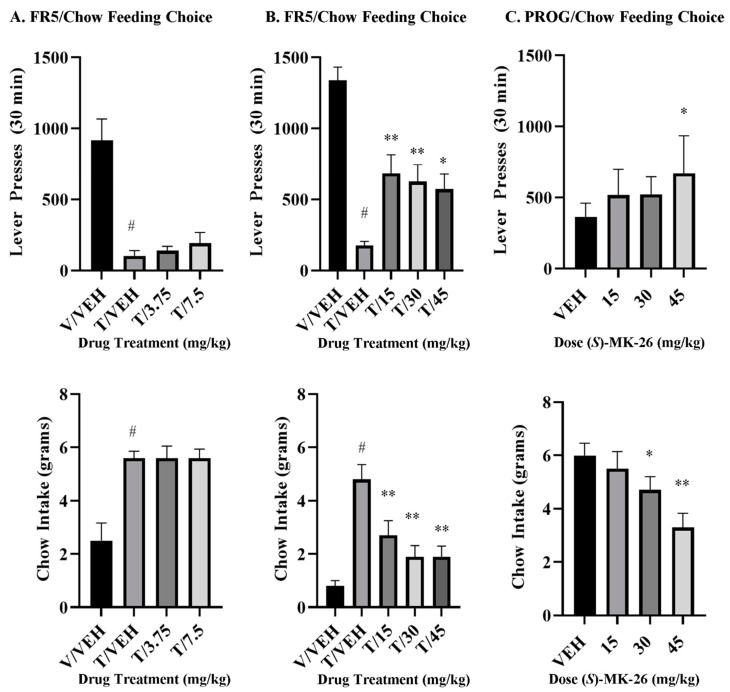
The effects of (*S*)-MK-26 on effort-based choice using the FR5/chow and PROG/chow feeding choice task. For FR5/chow procedure, rats (*n* = 8) received i.p. injections of vehicle plus vehicle (V/VEH), 1.0 mg/kg TBZ plus vehicle (T/VEH), or TBZ plus 3.75, 7.5 mg/kg for the low-dose progression (**A**), and 15.0, 30.0, or 45.0 mg/kg for the high-dose progression (**B**) of (*S*)-MK-26. For PROG/chow procedure, rats (*n* = 15) received i.p. injections of vehicle (VEH), 15.0, 30.0, or 45.0 mg/kg doses of (*S*)-MK-26 (**C**). Top: Mean (±SEM) number of lever presses during the 30 min session. TBZ plus vehicle significantly differed from vehicle plus vehicle (# *p* < 0.001); TBZ plus 15.0 and 30.0 mg/kg of (*S*)-MK-26 (** *p* < 0.01); and TBZ plus 45.0 mg/kg of (*S*)-MK-26 (* *p* < 0.05). For PROG/chow schedule, 45.0 mg/kg (*S*)-MK-26 significantly differed from vehicle (* *p* < 0.05). Bottom: Mean (±SEM) gram quantity of chow intake. TBZ plus vehicle significantly differed from vehicle plus vehicle (# *p* < 0.001); TBZ plus 15.0, 30.0 and 45.0 mg/kg of (*S*)-MK-26 (** *p* < 0.01). For PROG/chow schedule, 30.0 and 45.0 mg/kg of (*S*)-MK-26 significantly differed from vehicle (* *p* < 0.05 and ** *p* < 0.01, respectively).

Figure 9 shows the findings of the concurrent FR5/chow and PROG/chow feeding choice experiments. For FR5/chow feeding choice task, a repeated-measures analysis of variance (ANOVA) with the low dose of (*S*)-MK-26 yielded an overall treatment effect on the lever pressing (F_(3,21)_ = 20.84, *p* < 0.001; η^2^ = 0.75) and chow intake (F_(3, 21)_ = 10.20, *p* < 0.001; η^2^ = 0.59). Planned comparisons showed that TBZ shifted effort-based choice by decreasing lever pressing (F_(1,21)_ = 45.95, *p* < 0.001) and increasing chow intake (F_(1,21)_ = 21.01, *p* < 0.001).

However, using the low-dose progression (3.75 and 7.5 mg/kg), (*S*)-MK-26 failed to reverse the effects of TBZ (*p* > 0.05). To test the effects of high-dose progression on FR5/chow feeding choice task, a repeated-measures ANOVA yielded an overall treatment effect on the lever pressing (F_(4,28)_ = 15.80, *p* < 0.001; η^2^ = 0.69) and chow intake (F_(4,28)_ = 14.28, *p* < 0.001; η^2^ = 0.67). Planned comparisons showed that TBZ shifted effort-based choice by decreasing lever pressing (F_(1,28)_ = 60.85, *p* < 0.001) and increasing chow intake (F_(1,28)_ = 51.04, *p* < 0.001). Planned comparisons showed that coadministration of the doses 15.0, 30.0 and 45.0 mg/kg of (*S*)-MK-26 with TBZ significantly attenuated the effects of TBZ on lever pressing (F_(1,28)_ = 11.57, *p* < 0.01; F_(1,28)_ = 9.10, *p* < 0.01; and F_(1,28)_ = 7.12, *p* < 0.05, respectively) and chow intake (F_(1,28)_ = 14.60, *p* < 0.001; F_(1,28)_ = 26.81, *p* < 0.001; and F_(1,28)_ = 27.99, *p* < 0.001, respectively).

For analysis of the PROG/chow feeding choice task, non-parametric testing was used due to high variability in lever pressing (Shapiro–Wilk Normality test, *p* < 0.05). A non-parametric test for related samples, Friedman’s two-way analysis of variance by ranks, reported a statistically significant effect of treatment on lever presses (χ^2^ (3) = 9.80, *p* = 0.02). The pairwise comparisons yielded a statistically significant difference between the vehicle and the highest dose (45.0 mg/kg) after the Bonferroni adjustment (*p* = 0.011). To analyze chow intake, a repeated-measures ANOVA showed that there was a significant treatment effect (F_(3,42)_ = 8.476, *p* < 0.001). The planned comparisons revealed the two highest dose (30.0 and 45.0 mg/kg) decreased chow intake (F_(1,42)_ = 5.77, *p* < 0.05 and F_(1,42)_ = 23.76, *p* < 0.001, respectively).

## 4. Discussion

### 4.1. Design and Synthesis of (S)-MK-26

The initial efforts in our ongoing development of more potent and selective atypical DAT inhibitors, using modafinil as the starting scaffold, yielded several candidates [61,62], the most promising of which were CE-123 [63,64] and its more active (S)-enantiomer (analogue 3 in Figure 1) [43,64]. Further structure–activity relationship studies revealed that the introduction of a second chiral center to the scaffold via the para-substitution of one of its phenyl moieties greatly improves the pharmacological profile of our compounds [65,66]. An additional investigation of diastereomeric analogues’ potential led to the discovery of (*S*,*S*)-CE-158 (Figure 1), a singly meta-bromo substituted analogue of (*S*)-CE-123 with a markedly improved pharmacological profile [41]. The enantioselective synthesis of such diastereomeric compounds can be rather challenging. While the highly desirable pharmacological profile of (*S*,*S*)-CE-158 and its thus far demonstrated in vivo effects more than off-set difficulties of its current preparation, we developed the herein firstly described (*S*)-MK-26, seeking to preserve the desirable features whilst making the enantioselective synthesis significantly easier.

### 4.2. Relevance of DAT-Mediated Dopaminergic Regulation in the Young and Aging Brains

DAT plays a crucial role in the regulation of the levels of transsynaptic dopamine in the central nervous system, modulating a variety of cardinal biological processes, including motricity, emotion, food intake, reward, and learning and memory functions [67,68,69,70]. Consequently, DAT has been also associated with the occurrence of several brain neurological diseases [10,15,71,72,73,74,75]. The natural course of aging is generally accompanied by a marked detriment in several cognitive abilities and, in particular, comes with a striking reduction in cognitive functions, including memory storage and motivational drive [22,76,77,78,79]. Aging, in of itself, brings also profound alterations in dopaminergic activity [19,20,21]. The combined overlap of aging with alterations in dopaminergic signaling thus becomes a truly dominant risk factor for the presence of severe brain malaises, motivational disfunctions and other disorders including dementia and Alzheimer’s and Parkinson’s diseases [8,21]. The physiological relevance of dopaminergic activity in health and disease, in both young and the aged brains, has positioned the molecular elements of dopaminergic signaling, and in to particular DAT [10,15,72,74,75], as ideal targets in clinical pharmacotherapeutics and biomedical research [80]. However, there are several limitations with the commonly available pharmacological compounds used to aid in dopaminergic signaling deficiency [81,82]. These limitations include—but are not restricted to—poor absorption, blood–brain barrier impediments, short half-lives, fast neuroadaptation, low or transitory effectiveness, reduced target specificity, and unwanted side effects such as heart dysfunctions, altered cognition, and distorted motricity and sleep disturbances [29,81,82,83,84]. Other interventions, such as cell transplantations or deep brain stimulation, are not only not exempted of unwanted side effects [85,86], but are also very invasive, expensive, and the technology required for these procedures remains inaccessible in many countries [87,88]. Given all these limitations, over recent years, many research groups, including ours and collaborators, have embarked on a search for novel, effective and highly selective eugeroic/nootropic molecules, giving special emphasis to DAT as a primary target [34,40,41].

Dopaminergic signaling is central for the modulation of neuronal synaptic transmission and plasticity in the human hippocampus, a brain structure central for learning acquisition and memory retrieval functions [49,89,90,91,92] and which is also detrimentally affected by aging [8,76,78,93,94,95,96,97,98]. Numerous animal studies, particularly with rodents, have verified the evolutionarily-conserved relevance of the hippocampus for memory storage [99,100,101,102]; corroborated the detriments of hippocampus-dependent learning and memory resulting from aging [79,93,103,104,105,106,107]; and demonstrated the importance of dopaminergic signaling for hippocampal synaptic plasticity [50,108,109]. Moreover, not only is the human hippocampus markedly affected by aging, but profound dopaminergic alterations have been also described in Alzheimer’s disease and dementia [8,10,11,12,23,24,25], two neurological disorders directly linked to hippocampal morphofunctional deterioration [8,110,111,112,113]. The human hippocampus is moreover both structurally and functionally associated with other brain structures enriched in molecular dopaminergic complexes that modulate memory and mood-related functions [114,115,116]. This morpho-functional dopaminergic interconnectivity is also highly conserved across many species, highlighting its pivotal biological relevance. For example, immunochemical and functional studies from rodents and primates have described that the hippocampus receives active dopaminergic regulatory inputs originating from the nucleus accumbens and the ventral tegmental areas [53,117,118,119], and hippocampal dopaminergic complexes participate in the regulation of memory-related synaptic plasticity [51,120,121].

The fact that the hippocampus itself is not so profusely enriched in the expression of molecular elements belonging to the dopaminergic system, compared to other brain structures such as the VTA or the nucleus accumbens, could lead to the misperception that dopaminergic activity is not that functionally relevant in the hippocampus [122]. However, abundant evidence highlights the relevance of dopaminergic signaling for hippocampus-dependent memory functions in vivo and in vitro [51,52,53,98,115,117,120,123,124,125,126,127,128,129,130,131]. Moreover, the hippocampus is also structurally and functionally linked to dopaminergically enriched brain structures such as the nucleus accumbens, the VTA and the striatum [125,132], forming a dynamically interactive circuit that has been associated with neurological disorders such as schizophrenia and depression [8,21,110,114,133]. The hippocampus thus has, in its own right, all the structural and functional elements of classical dopaminergically hot structures, including of course the presence of uptake areas [134] that could act in vivo as potential target regions for (*S*)-MK-26 and other newly generated/selective DAT inhibitors. Our findings on (*S*)-MK-26 therefore provide a valuable avenue in the search for additional potential applications in currently established experimental animal models of dopamine-related diseases [135], and additional behavioral studies are encouraged using other modalities of hippocampus-dependent memory tasks.

### 4.3. (S)-MK-26 Reverses TBZ-Induced Effort-Related Motivational Dysfunctions and Increases Selection of High-Effort Choices

Consistent with previous studies [31,136], TBZ produced an effort-related motivational impairment in rats tested on the FR5/chow feeding choice task; it reduced work output on the lever pressing component, but increased the intake of the concurrently available chow. In the higher dose range of 15.0–45.0 mg/kg but not the lower dose range, (*S*)-MK-26 significantly but partially reversed the effects of TBZ, increasing lever pressing and decreasing chow intake in TBZ-treated rats. Furthermore, when administered alone to animals trained on the PROG/chow feeding task, (*S*)-MK-26 enhanced the exertion of effort, significantly increasing high-effort PROG lever pressing, and reducing chow intake. Taken together, the effects of (*S*)-MK-26 on these tests of effort-based choice are similar to the results of several other drugs that inhibit DAT, including the antidepressant bupropion [136,137,138], modafinil [31], and the modafinil analogs (*S*)-CE-123 and (*S*,*S*)-CE-158 [39,40]. When compared to the aforementioned modafinil analogs, (*S*)-MK-26 appears to have a lower potency than (*S*,*S*)-CE-158 but has a similar potency to (*S*)-CE-123. Based upon its ability to enhance effort-related aspects of motivation, it is possible that (*S*)-MK-26 could be useful for treating the motivational dysfunctions seen in depression and other disorders.

### 4.4. (S)-MK-26 and Modafinil

(*S*)-MK-26 is structurally related to the DAT inhibitor modafinil, which is a eugeroic/cognition-enhancing compound recently approved by the U.S. Food and Drug Administration for specific clinical applications [139,140]. However, (*S*)-MK-26 is about 130 times stronger as a DAT blocker compared to modafinil, which has a rather low DAT-inhibiting power (IC_50_ = 6.4 µM, [141]) vs. the IC_50_ = 0.049 µM of (*S*)-MK-26 described here. Moreover, modafinil (with a still controversial low-abuse liability [142,143,144,145]) is also not exempted of unwanted side effects [146]; its effectiveness in applications such as the treatment of drug dependency remains debated [147,148], and modafinil is also not as specific as (*S*)-MK-26, as modafinil also targets and blocks the norepinephrine transporter and alters histamine and serotoninergic activity [140,141]. The virtual absence of activity of (*S*)-MK-26 on the norepinephrine transporter, its superior potency on DAT, and the capability of (*S*)-MK-26 to enhance transsynaptic dopamine and to augment both hippocampal synaptic plasticity and hippocampus-dependent learning and memory functions in the aging brain thus highlights (*S*)-MK-26 as a highly promising and very suitable/selective DAT blocker for its potential use in clinical research. Moreover, the preliminarily effects of (*S*)-MK-26 on learning and memory upon acute dosage administration examined here further suggest that (*S*)-MK-26 could give rise to fewer abuse-risk concerns due, e.g., to excessively augmented dopamine-boosting effects on addiction-related brain structures such as the dorsal striatum, which is highly involved in the development of dopamine-mediated drug dependence [149]. However, as described in the experiments shown in Figure 2, whereas (*S*)-MK-26 has more effect on DAT, there are also some minor yet detectable effects of (*S*)-MK-26 on other neuronal receptors that, although very unlikely given the differences in selectivity compared to DAT, cannot be completely ruled out as potential off-targets mediating in some of the behavioral, electrophysiological and other effects observed for (*S*)-MK-26 in this work. Off-target effects have been described for modafinil and its analogues [150]. Our observations therefore imply that, together with NET and SERT, other signaling pathways besides DAT could account for the general physiological effects of (*S*)-MK-26.

Thus, while a great deal of attention has been paid to modafinil in both experimental and clinical/applied research [151,152], several limitations regarding the effectivity and degree of specificity of modafinil have encouraged the search for alternative modafinil analogues and other molecules acting on the DAT signaling system with potential therapeutic applications [39,40,41,43,150]. Nevertheless, modafinil continues prevailing as an attractive compound in experimental biomedical research. Recent findings derived from electroencephalographic studies in healthy adult humans describe that modafinil promotes higher-order neuronal circuitry activity by inducing the deactivation of large-scale sensory networks [153]. Other experiments have also recently addressed the changes in the effects of modafinil when administrated in combination with approved antidepressant drugs and obtained very interesting results [154]. However, and while additional research is still required to reach undisputable final conclusions, potential and quite alarming adverse effects compromising the safety of modafinil under specific circumstances have also recently started to emerge [155,156]; see also [157].

## 5. Limitations

The data presented in this work were exclusively derived from basic research conducted using male rats as the experimental animal model and, therefore, our results must be interpreted without making biological generalizations. Further independent research with both male and female rats, as well as with other animal models, must therefore be conducted in order to investigate any potential positive or possible negative effects of (*S*)-MK-26 on other major brain structures not examined here (including the substantia nigra and the ventral tegmental area [158]), as well as on the peripheral nervous systems, since (*S*)-MK-26 could potentially also act on other organs that present with dopaminergic molecular components, and in which DAT activity might occur (see, for example, [159,160,161,162]). Additionally, controversy still exists around the proper terminology and the definitions used when referring to concepts relating to the age of animals (see also [163,164,165] for age-related (e.g., young vs. old) terminology and definitions). Thus, the appropriate selection of the age of the animals used in experimental studies is critical not only because age can dramatically influence the properties of several different brain neuronal functions, but also when one wants to establish analogies with similar cellular mechanisms and related systemic functions that can occur in the aged human brain [165,166,167]. All these different factors (e.g., strain, sex, etc.; [55,168,169,170,171,172,173,174,175]) could thus distinctly influence the outcome of the (*S*)-MK-26 pharmacological treatment. Indeed, several reports have described that brain physiological processes and dopamine-associated neuropathological maladies are differentially manifested depending on the sex of the patient [176,177,178,179], as is the case for Parkinson’s disease, which manifests predominantly in aged men. Our work therefore invites further independent and exhaustive research aiming to further characterize the nootropic properties of (*S*)-MK-26 in different species, on female subjects, and in other brain areas more classically known as dopaminergic, such as the nucleus accumbens.

## 6. Materials and Methods

### 6.1. Synthesis

Reaction conditions and yields were not systematically optimized. Anhydrous solvents were purchased from Sigma-Aldrich and were used as such without further purification. All other chemicals and reagents were purchased from Sigma-Aldrich, TCI Europe/Germany, Synthonix, Acros Organics, Activate Scientific, Fluorochem, Enamine and Alfa Aesar.

^1^H and ^13^C NMR spectra were recorded on a Bruker Avance 500 NMR spectrometer (UltraShield) using a 5 mm switchable probe (PA BBO 500SB BBF-H-D-05-Z, 1H, BB = ^19^F and ^31^P–^15^N) with z axis gradients and automatic tuning and matching accessory (Bruker BioSpin). The resonance frequency for ^1^H NMR was 500.13 MHz and for ^13^C NMR was 125.75 MHz. All measurements were performed for a solution in fully deuterated chloroform or DMSO at 298 K. Standard 1D and gradient-enhanced (ge) 2D assays, such as double-quantum-filtered (DQF) COSY, NOESY, HSQC, and HMBC, was used as supplied by the manufacturer. Chemical shifts are referenced internally to the residual, non-deuterated solvent signal for chloroform ^1^H (*δ* = 7.26 ppm) or DMSO ^1^H (*δ* = 2.50 ppm) and to the carbon signal of the solvent for chloroform ^13^C (*δ* = 77.00 ppm) or DMSO ^13^C (*δ* = 39.57 ppm).

HRESIMS spectra were obtained on a maXis UHR ESI-Qq-TOF mass spectrometer (Bruker Daltonics, Bremen, Germany) in the positive-ion mode. Samples were dissolved in ACN/MeOH/H2O 99:99:2 (*v*/*v*/*v*) and directly infused into the ESI source with a syringe pump. The ESI ion source was operated as follows: capillary voltage: 4.2 kV; nebulizer: 0.4 bar (N2); dry gas flow: 4 L/min (N2); and dry temperature: 150 or 180 °C. The sum formulas of the detected ions were determined using Bruker Compass DataAnalysis 5.1 based on the mass accuracy (Δ *m*/*z* ≤ 5 ppm) and isotopic pattern matching (SmartFormula algorithm).

The overall purity of the compounds was determined by HPLC on an UltiMate 3000 series system equipped with VWD detector (Dionex/Thermo Fisher Scientific, Germering, Germany). Separation was carried out on an Acclaim 120 C18, 2.1 mm × 150 mm, 3 µm HPLC column (Thermo Fisher Scientific) using LC-MS-grade water and acetonitrile as mobile phases A and B, respectively. The sample components were separated and eluted with a linear gradient from 10% to 90% B for 25 min followed by an isocratic column cleaning and re-equilibration step. The flow rate was 0.2 mL/min and the column oven temperature was set to 25 °C. The purity was determined from the UV chromatogram (254 nm) as the ratio of the peak area of the compound to the total peak area (i.e., the sum of the areas of all peaks that were not present in the solvent blank). Based on the HPLC data, all final compounds were ≥95% pure.

The enantioselectivity of asymmetric synthesis of (*S*)-MK-26, as well as chiral purity of individual enantiomers (*S*)-MK-26 and (*R*)-MK-26 ((1) and (9) in Schema 1, respectively) accrued after chiral resolution of its racemic synthesis were assessed via an LC-2010A HT Liquid Chromatograph device (Shimadzu Corporation, Tokyo, Japan) using a Chiralpack IA analytical column (4.6 × 250 mm, 5 µm) (Diacel Inc., Tokyo, Japan) with the column oven temperature set to 25 °C. HPLC-grade ethyl acetate at 1 mL/min flow rate was used as the mobile phase to measure chiral purity of enantiomers resolved after racemic oxidation, while a 7 to 3 mixture of HPLC-grade ethyl acetate and hexane was used as the mobile phase in analysis of enantioselectively synthesized (*S*)-MK-26.

### 6.2. 5.-(((B(3-chlorophenyl)methyl)thio)methyl)thiazole (7)

An amount of 1.62 g (6.0 mmol) of 3,3′-dichlorobenzhydrol was dissolved in 20 mL of acetic acid in a 100 mL round-bottomed flask equipped with a magnetic stirring bar; 0.78 g (6.0 mmol) of thiazol-5-ylmethanethiol and 1.68 mL (6.6 mol, 1.1 equivalent) of boron trifluoride diethyl etherate were added to the mixture and the reaction proceeded under reflux until total expenditure of the starting alcohol and mercaptan, as monitored via thin-layer chromatography. The reaction mixture was poured into a 500 mL Erlenmeyer flask equipped with a magnetic stirring bar and containing ice and around 150 mL of water. Acetic acid was neutralized with gradual addition of 28 g of NaHCO_3_ powder, the mixture was transferred to a 500 mL separation funnel and the product was extracted 3 × with 100 mL portions of ethyl acetate. The extracts were pooled, washed in sequence with 100 mL of 5% NaHCO_3_ and with 100 mL of brine, dried over anhydrous Na_2_SO_4_, filtered and condensed under reduced pressure to yield a crude oily mixture which was purified by column chromatography on silica gel (2 to 1 mixture of petroleum ether and ethyl acetate was used as the mobile phase) to afford 1.92 g of the desired product, corresponding to an 88.64% yield (see also Schema 1).

### 6.3. 5.-(((B(3-chlorophenyl)methyl)sulphinyl)methyl)thiazole (8)

An amount of 0.64 g (1.8 mmol) of **7** was dissolved in 10 mL of acetic acid in a 100 mL round-bottomed flask equipped with a magnetic stirring bar; 0.18 mL (1.8 mol) of 30% H_2_O_2_ was introduced via a syringe and the reaction proceeded overnight at room temperature. The reaction mixture was poured into a 500 mL Erlenmeyer flask equipped with a magnetic stirring bar and containing ice and around 150 mL of water. Acetic acid was neutralized with gradual addition of 14 g of pulverised NaHCO_3_, the mixture was transferred to a 500 mL separation funnel and the product was extracted 3 × with × 100 mL portions of ethyl acetate. The extracts were pooled, washed in sequence with 100 mL of 5% NaHCO_3_ and with 100 mL of brine, dried over anhydrous Na_2_SO_4_, filtered and condensed under reduced pressure to afford 0.66 g of the racemate of the desired product, which presented as a single spot under thin-layer chromatography and was without further manipulation resolved to individual enantiomers (see also Schema 1).

### 6.4. (S)-5-(((B(3-chlorophenyl)methyl)sulphinyl)methyl)thiazole (S)-MK-26 and (R)-5-(((bis(3-chlorophenyl)methyl)sulphinyl)methyl)thiazole (9)

Compound 8 (see also Schema 1), was resolved to individual enantiomers on a Shimadzu 10AVP HPLC System (Shimadzu Corporation, Tokyo, Japan) equipped with Chiralpak IA semipreparative column (10 mm diameter × 20 cm length) (Chiral Technologies Europe, France) using pure HPLC-grade ethyl acetate as the mobile phase and employing stacked injection method, similar to one previously described [180]. This afforded 0.28 g of the less retained enantiomer, (*S*)-MK-26, and 0.28 g of the more retained enantiomer, (*R*)-MK-26 ((1) and (9) in Schema 1, respectively), corresponding to a total yield of 83.23%.

1. ^1^H NMR (500 MHz, CDCl_3_) δ: 8.88 (1H, s); 7.71 (1H, s); 7.41–7.27 (8H, m); 4.57 (1H, s); 4.17 (1H, d, ^2^J_HH_ = 14.45 Hz); 3.92 (1H, d, ^2^J_HH_ = 14.45 Hz). ^13^C NMR (125 MHz, CDCl_3_) δ: 155.16 (CH); 144,29 (CH); 136.20 (C); 135.62 (C); 135.36 (C); 134.85 (C); 130.93 (CH); 130.14 (CH); 129.32 (CH); 129.29 (CH); 129.06 (CH); 128.67 (CH); 127.60 (CH); 126.70 (CH); 124.51 (C); 68.55 (CH); 46.96 (CH_2_). HRESIMS *m*/*z*: 381.9890 [M+H]^+^ (calculated for C_17_H_14_Cl_2_NOS_2_^+^, 381.9888, err. −0.4 ppm) and 403.9704 [M+Na]^+^ (calculated for C_17_H_13_Cl_2_NNaOS_2_^+^, 403.9708, err. 1.0 ppm). Overall purity: 99.76%; ret. Time: 23.70 min; Chiral purity: 99.53%; ret. time: 6.03 min.

9. ^1^H NMR (500 MHz, CDCl_3_) δ: 8.88 (1H, s); 7.71 (1H, s); 7.41–7.27 (8H, m); 4.57 (1H, s); 4.17 (1H, d, ^2^J_HH_ = 14.45 Hz); 3.92 (1H, d, ^2^J_HH_ = 14.45 Hz). ^13^C NMR (125 MHz, CDCl_3_) δ: 155.16 (CH); 144.29 (CH); 136.20 (C); 135.62 (C); 135.36 (C); 134.85 (C); 130.93 (CH); 130.14 (CH); 129.32 (CH); 129.29 (CH); 129.06 (CH); 128.67 (CH); 127.60 (CH); 126.70 (CH); 124.51 (C); 68.55 (CH); 46.96 (CH_2_). HRESIMS m/z: 381.9889 [M+H]^+^ (calculated for C_17_H_14_Cl_2_NOS_2_^+^, 381.9888, err. −0.2 ppm) and 403.9702 [M+Na]^+^ (calculated for C_17_H_13_Cl_2_NNaOS_2_^+^, 403.9708, err. 1.6 ppm). Overall purity: 99.89%; ret. time: 23.72 min; Chiral purity: 99.50%; ret. time of 9.54 min.

Enantioselective synthesis of **1**. An amount of 0.924 g (2.5 mmol) of **7** was dissolved in 10 mL of HPLC-grade acetone (Sigma-Aldrich) in a 100 mL round-bottomed flask equipped with a magnetic stirring bar. A total of 0.26 mL (1.5 mmol, 0.6 equivalent) of (+)-diisopropyl L-tartrate was added to the mixture via a syringe, followed by 0.22 mL (0.75 mmol, 0.3 equivalent) of titanium (IV) isopropoxide. After 5 min of stirring, 6.75 µL (0.375 mmol, 0.15 equivalent) of distilled water was added, for a total ratio of alcoxide to tartrate to water of 1:2:0.5. It is crucial that these three be introduced exactly in the specified order. Two minutes after addition of water, the flask was connected to a reflux and the reaction mixture refluxed for 1 h; following this, the mixture was given 10 min to cool down to room temperature, 90 µL (5 mmol, 0.2 equivalent) of *N*,*N*-diisopropylethylamine was added and after 10 min of stirring the flask was equipped with a dropping funnel through which 0.51 mL (2.5 mmol, 1 equivalent) of cumene hydroperoxide dissolved in 5 mL of HPLC-grade acetone was introduced over 20 min. The reaction ran for a total of 3 h, until the complete expenditure of the starting thiol, as monitored via thin-layer chromatography. An amount of 15 mL of water was added to the reaction mixture, which was stirred for 20 min to cause precipitation of titanate species, and was filtered through a layer of celite on a Hirsch funnel connected to a vacuum pump. The celite cake was washed with 35 mL portions of acetone and the collected filtrate was condensed under reduced pressure to a brown oily residue, which was dissolved in 75 mL of dichloromethane in a 500 mL Erlenmeyer flask equipped with a magnetic stirring bar. A total of 75 mL of 1 to 1 mixture of brine and 1 M NaOH was added to the organic solution and stirred vigorously for 1 h to de-esterify residual diethyl tartrate. The mixture was transferred to a 250 mL extraction funnel, the organic phase was isolated and the aquatic phase was treated with 3 additional 50 mL portions of dichloromethane. Organic extracts were pooled, washed in sequence with 100 mL of 5% citric acid and with 100 mL of brine, dried over anhydrous Na_2_SO_4_, filtered and condensed under reduced pressure to afford 0.80 g of the colorless viscous oily desired product, corresponding to an 83.7% yield.

Asymmetrically synthesized **1**. ^1^H NMR (500 MHz, CDCl_3_) δ: 8.83 (1H, s); 7.66 (1H, s); 7.36–7.23 (8H, m); 4.53 (1H, s); 4.13 (1H, d, ^2^J_HH_ = 14.45 Hz); 3.88 (1H, d, ^2^J_HH_ = 14.45 Hz). ^13^C NMR (125 MHz, CDCl_3_) δ: 155.12 (CH); 144,28 (CH); 136.19 ©; 135.58 (C); 135.35 (C); 134.81 (C); 130.90 (CH); 130.12 (CH); 129.26 (CH); 129.30 (CH); 129.03 (CH); 128.64 (CH); 127.58 (CH); 126.68 (CH); 124.49 (C); 68.51 (CH); 46.95 (CH_2_). HRESIMS *m*/*z*: 381.9885 [M+H]^+^ (calculated for C_17_H_14_Cl_2_NOS_2_^+^, 381.9888, err. 1.0 ppm) and 403.9702 [M+Na]^+^ (calculated for C_17_H_13_Cl_2_NnaOS_2_^+^, 403.9708, err. 1.4 ppm). Overall purity: 99.78%; ret. Time: 23.70 min; Chiral purity: 96.43%; ret. Time: 10.27 min.

### 6.5. Determination of Absolute Configuration

This part of the work was outsourced to BioTools Inc. (Jupiter, Florida, FL, USA). Absolute configuration of single enantiomers 1 and 9 (see Schema 1) was determined via comparison of their respective measured and predicted VCD spectra. For detail, please refer to the Supplementary Materials.

### 6.6. Uptake Inhibition Assays

Human embryonic kidney 293 cells (HEK293 cells) were cultivated in Dulbecco’s Modified Eagle Medium (DMEM), containing 10% heat-inactivated fetal calf serum (FCS; 100 mL/L), streptomycin (100 μg/100 mL) and penicillin (100 U × HEK293). Geneticin (50 μg/mL) was added to select cells stably expressing the human isoforms of dopamine, norepinephrine and serotonin transporter (hDAT, hNET, and hSERT, respectively) as described earlier [181,182]. Cell lines were maintained in culture in humidified atmosphere (37 °C, 5% CO_2_), and passaged when reached 80–90% confluency. Cells expressing the desired transporter were seeded the day before the experiment (40,000 cells per well) or two days before the experiments (20,000 cells per well) onto poly-D-lysine (PDL)-coated 96-well plates.

Uptake inhibition experiments were carried out as reported previously [144]. Ninety-six-well plates containing hDAT-, hNET- or hSERT-expressing cells were washed with Krebs-HEPES buffer (KHB; 25 mM HEPES, 120 mM NaCl, 5 mM KCl, 1.2 mM CaCl_2_, and 1.2 mM MgSO_4_ and 5 mM D-glucose, pH = 7.3). Cells were then preincubated with increasing concentrations of the compound diluted in KHB for 6 min. Following the pre-incubation of the cells with the compound, the tritiated substrate (hDAT: 0.15 μM [^3^H]DA; hNET: 0.05 μM [^3^H]MPP^+^; hSERT: 0.1 μM [^3^H]5-HT) was added to the wells. The uptake was terminated by removal of tritiated substrate and by washing the cells with ice-cold KHB after 1 min (for hDAT and hSERT) or 3 min (for hNET). Thereafter, 200 μL of Ultima Gold^TM^ XR scintillation cocktail (PerkinElmer, MA, USA) was added, the plates were shaken and the radioactivity accumulated into the cells determined in a Wal-lac 1450 MicroBeta TriLux Liquid Scintillation Counter & Lumi (GMI, Ramsey, MN, USA). Non- specific tritiated substrate uptake was measured in the presence of 100 μM cocaine (for hDAT and hNET) or 10 μM paroxetine (for hSERT), and subtracted from the data. Uptake in the presence of test drugs was normalized to the uptake in the presence of the vehicle.

### 6.7. hDAT, hNET and hSERT Binding Assays

Binding affinity to the hDAT, hNET and hSERT expressed in CHO cells was evaluated in in vitro radioligand binding assays. Binding assays were conducted according to the standard and validated protocols under conditions defined by the contractor (Eurofins Cerep SA, Celle-’Evescault, France; study number 100054410). Antagonist radioligand assays were conducted for the human norepinephrine, the human dopamine, and the serotonin transporters (using CHO cells), using [^3^H]nisoxetine (1 nM), [^3^H]BTCP (4 nM), and [^3^H]imipramine (2 nM) as ligands and desipramine (1 µM), BTCP (10 µM), and imipramine (10 µM) as non-specific agents, respectively (with scintillation counting as detection method).

### 6.8. Plasma and Brain Compound Levels and Pharmacokinetic Parameters

The bioavailability of (*S*)-MK-26 after intraperitoneal (i.p.) administration in male Sprague–Dawley rats (260–351 g) was outsourced to Evotec (Toulouse CEDEX, France, Study Number VDD10431). (*S*)-MK-26 at dose of 10 mg/kg of body weight was i.p. administered and blood samples and brains were collected at 0.083, 0.25, 0.5, 1, 3, 7, 10, and 24 h post-administration (3 rats per time point). Plasma and brain levels of (*S*)-MK-26 were measured by LC-MS by a standard validated protocol defined by the contractor, and the pharmacokinetic parameters were calculated from individual concentrations by WinNonLin7.0 (Phoenix64), a non-compartmental model. No adverse effects were observed during the study.

### 6.9. In Vivo Microdialysis

Male Sprague Dawley rats (250–350 g, Janvier, France) were used for all in vivo microdialysis experiments. Animals were kept in groups of four in standard cages (38 cm × 60 cm × 20 cm) and were acclimated to the facility for at least ten days before the beginning of the experiment under controlled environmental conditions (22 ± 2 °C, 60% humidity, 12 h light/dark cycle, with lights on from 08:00 to 20:00 h), with water and standard laboratory food provided ad libitum. To limit the stress due to manipulation during the experiments, each animal was daily handled for approximately 1–2 min throughout the habituation period and contact with the animal house maintenance personnel was limited to a single attendant. The bedding in the home cages was never changed, neither the day before nor the day of the experiment. All experiments were performed between 10:00–18:00 h. (*S*)-MK-26 (1 or 10 mg/kg) or vehicle (Kolliphor 30% in distilled water) were freshly prepared on the day of the experiment and i.p. administered at a volume of 1.5 mL/kg. The day before the microdialysis experiment, rats were positioned in a stereotaxic apparatus (Stoelting Co., Wood Dale, IL, USA) and under isoflurane anaesthesia (1.5–2%) (Harvard Apparatus, Holliston, MA, USA) implanted with a vertical microdialysis probe. A probe with dialysis membrane of approximately 2–3 mm of free surface (prepared as previously described [183,184]) was directed unilaterally at the mPFC (PrL and IL; coordinates: 3.0 mm anterior and 0.7 mm lateral to bregma, and 5.5 mm ventral to dura), at the NAc shell (coordinates: 2.0 mm anterior and 0.8 mm lateral to bregma, and 8.0 mm ventral to dura) or NAc core (coordinates: 1.6 mm anterior and 1.6 mm lateral to bregma, and 7.6 mm ventral to dura), according to the rat brain atlas [45]. On the day of the experiment, the animals were transferred to a sound-proof room and after 1 h habituation period, the microdialysis probe was connected with polyethylene tubing to a CMA/100 microinfusion pump (Harvard Apparatus, Holliston, MA, USA) and perfused with a Ringer’s solution (147 mM NaCl, 3 mM KCl and 1.2 mM CaCl_2_, pH 6.5) at a flow rate of 2.5 µL/min. After an equilibration period of a 2 h of the perfusion medium with the extracellular fluid, dialysate aliquots of 37.5 µL were collected (every 15 min during the experiment) in polyethylene tubes kept on ice for the determination of dopamine concentrations. After collecting at least four dialysate aliquots, rats were i.p. injected with vehicle or (*S*)-MK-26 (1 or 10 mg/kg), and ten more dialysate aliquots were collected every 15 min. Dopamine concentrations in the dialysates from the NAc (core and shell) and mPFC were measured by high pressure liquid chromatography (HPLC) on a 7.5 cm × 3.0 mm i.d., Supelcosil C18, 3 µm particle size column, (Supelco, Supelchem, Milan, Italy) coupled to electrochemical detection (Coulochem II, ESA, Cambridge, MA, USA) using a 4011 dual cell, as previously described [183,184]. Detection was performed in reduction mode, with potentials set to +350 and −180 mV. The mobile phase was 0.06 M citrate/acetate pH 4.2, containing methanol 20% *v*/*v*, 0.1 mM EDTA, 1 µM triethylamine, and 0.03 mM sodium dodecyl sulphate at a flow rate of 0.6 mL/min. The sensitivity of the assay was 0.125 pg. At the end of the experiments, the rats were sacrificed by decapitation; the brains were immediately removed from the skull and stored in 4% aqueous formaldehyde for 12–15 days. After this period, 40 µm coronal brain sections were prepared with a freezing microtome, stained with Neutral Red and inspected on a phase contrast microscope. The position of the tip of the microdialysis probe in the NAc (core and shell) and mPFC were then corroborated by following the tract of the microdialysis probe through a series of brain sections. Only rats with the active part of the dialyzing membrane positioned correctly in the NAc (core and shell) and mPFC were considered for the statistical evaluation of the results.

### 6.10. Hippocampal Slice Preparation and Electrophysiology

All electrophysiological experiments were conducted post mortem. Hippocampal slices (*n* = 12 animals/group) were prepared 1 h after the pharmacological treatments (animal experiments conducted under BMBWF-66.009/235/V/3b/2018 regulations). Animals underwent a brief sedative exposure to low CO_2_ inhalation and were immediately afterwards euthanized using a sharp-blade guillotining (DCAP-M, World Precision Instruments, Inc., Sarasota, FL, USA). The brains were removed and transferred to an ice-cold artificial Cerebrospinal Fluid (aCSF) solution containing (in mM): 125 NaCl, 2.5 KCl, 20 NaHCO_3_, 2.5 CaCl_2_, 1 MgCl_2_, 25 D-glucose, 1 NaH_2_PO_4_ (pH 7.4). Hippocampi were extracted and cut transversally with a tissue chopper (McIlwain TC752, Campden Instruments LTD., Loughborough, England) to obtain 400 µm-thick slices. Before electrophysiological assays, the slices rested for at least 1 h in a submerged recovery chamber (filled with aCSF) placed in a water bath with the temperature set at 30 °C. The aCSF solution was bubbled with a carbogen gas mixture (95% medical O_2_ + 5% medical CO_2_).

Slice recordings were carried out in a submerged chamber receiving 3–4 mL/min of carbogenated aCSF (30 ± 2 °C). Field excitatory postsynaptic potentials (fEPSPs) were recorded using glass pipettes made of pulled capillary-glass obtained from Harvard Apparatus (Harvard Apparatus, GmbH; Hugo Sachs Elektronik, Germany). Evoked fEPSPs were recorded at the CA1 stratum-radiatum layer after electrically stimulating the collateral projections of Schaffer coming from the CA3 region as previously reported [56,185]. For the recordings, the hippocampi were divided into four major sections along the septo-temporal axis, taking its anatomical transversal middle as the absolute center, and slices within the approximately 30–40% section from the nominal center towards the ventral region (longitudinally) were selected for recordings; a region that has been previously showing to display robust LTP responses [186,187] and that receives inputs from dopaminergic neurons projecting from the locus coeruleous and ventral tegmental areas [188]. Recording pipettes were pulled with a P-87 horizontal puller (Sutter Instrument, Novato, CA) and filled with aCSF (series resistances were of 3 ± 1 MΩ). fEPSPs were evoked by delivering square biphasic steps of electrical pulses via bipolar electrodes made of Teflon-coated tungsten wire isolated to the tip (~50 µm diameter tip). An ISO-STIM 01D stimulator (NPI Electronics, Tamm, Germany) was used to provide electrical stimulation. Basal synaptic transmission was assessed by the generation of input/output (I/O) curves, resulting from the plotting of the field slope values (output; normalized to maximum) against the presented electrical stimulus intensity (input voltage). Input voltages consisted of increasing square pulses of electrical stimulation of 200 μs (0–9 V pulses were given with 1 V increments and 15 s inter-pulse intervals). LTP was induced by applying 5 stimulation bursts (each consisting of 10 electrical pulses delivered 100 Hz (200 μs/pulse)) with 500 ms intervals (see also [54,55,56,189,190]). I/O curves and LTP measurements were examined using voltage stimulation intensities that elicited 40–50% of the maximum evoked field amplitude (with no changes in that 40–50% stimulus intensity introduced for LTP-inducing high frequency stimulation (HFS)). LTP was determined from the temporal progression of the fEPSP slope values (decaying phase) after LTP induction, normalized to the averaged slope values obtained before LTP-induction (base line values). The data for LTP from all slices were averaged for each animal (2–5 slices per rat were measured and averaged to yield one single value per animal). Recordings were obtained using a SEC-10L amplifier (NPI electronics, Tamm Germany) via an LIH-8+8 interface (HEKA Elektronik, Germany) and using the PATCHMASTER and the FITMASTER software packages (both from HEKA Elektronik, Germany) for the acquisition and the analysis of data, respectively.

### 6.11. Open Field

Animals were habituated to the experimental room twenty-four hours prior to the start of the experiment. The open field consisted of a black wooden board (1.20 m × 1.20 m) surrounded by white wooden walls (50 cm in height); light was adjusted to 150 lux. Each animal was placed in the middle of the open-field arena (animal experiments conducted under BMBWF-66.009/235/V/3b/2018 regulations) and behavior was recorded for 10 min. Data analysis was performed using the AnyMaze software (Stoelting Co., IL, USA). The test consisted of one habituation session on the first day and one test session on the following day. Each animal received (*S*)-MK-26 at 10, 20, 0 and 1 mg/kg of body weight with wash-out period of nine days between each treatment.

### 6.12. Hole-Board Test

In order to examine the effect of (*S*)-MK-26 on hippocampus-dependent spatial reference memory, we used the standardized hole-board test [47,191] (animal experiments conducted under BMBWF-66.009/235/V/3b/2018 regulations) following published procedures [47,191,192,193] with minor modifications. In brief, food restriction and controlled body weight loss with it (until steadily reaching 85% of the average body weight sustained during ad libitum feeding) was initially imposed for this experimental study as cognitive/behavioral incentive. A black plastic hole-board maze (1 m^2^ base) with 16 symmetrically distributed holes located at the floor (7 cm in diameter and depth) and walls enriched with spatial cues was used (See Figure 4). Accessory distal cues were also visible in the walls of the experimental room. Dustless precision food pellets (45 mg, Bioserv^®^, Flemington, NJ, USA) were placed as motivational baits in four randomly designated holes, unchanged throughout the test. Several inaccessible pellets were also placed all across the entire area immediately below all the holes of the board maze in order to induce a uniform distribution of the food smell and thus preventing the acute orientating response of the sense of smell from prevailing over the evocation of spatial memories associated with the location of accessible food. Animals were allowed to familiarize to the experimental room and handled by the same experimenter during 15 min/day for 3 days in order to get them familiarized to human interaction. Animals went next throughout a 3-day habituation phase. For this, they were allowed to explore—and familiarize with—the maze environment (15 min/day; the first habituation day, a number of 10 food pellets were distributed outside the holes; for the second and third habituation days, all holes were filled with food pellets). On the third day of the board habituation phase, animals from the experimental group received the first (*S*)-MK-26 treatment, whereas the controls just received the Kolliphor vehicle. During the learning acquisition and memory retention phases, the animals were exposed for four days to different sessions as follows: Day-1 (5 learning sessions; with a first trial of 4 min followed by four trials of 2 min; time intervals between trials were 30 min between 1&2 and 20 min between the following ones); Day-2 (4 learning sessions; with all trials lasting 2 min and 20 min between trials); Day-3 (3 learning sessions; with all trials lasting 2 min and 20 min between trials); Day-4 (1 session). Animals were i.p. injected once every day with either vehicle or (*S*)-MK-26 (see also Animals and Pharmacological Treatments) 30 min before the learning sessions. The entire hole-board maze was cleaned and disinfected (with 1% Incidin) after every session to eliminate remaining secretions, excretions and other possible odor cues from the previous animal that could alter the subsequent behavioral examinations. The behavioral experiments were conducted in a noise-isolated room; all sessions were monitored by a distal overhead conventional digital camera and behavioral data digitalized using the ANY-Maze video tracking system (Stoelting Europe, 8-Terenure Place, Terenure, Dublin, D6W Y006, Ireland). Spatial memory performance was assessed from the number of errors made by the animal, that is, the number of times the animals visit unbaited holes (reference memory). A reference memory index (RMI) was estimated from: (the number of total visits to baited holes/the number of total visits to all available holes). Thus, from these measurements, an RMI value of “1” would constitute quantifiable indicator of optimal reference memory, with a performance without empty-hole visiting mistakes. Rats with fewer than 52-hole entries in total over the thirteen trials were excluded from the analysis. Based on this criterium, two animals from the vehicle group and one animal from (*S*)-MK-26 group were removed from the analysis. All behavioral training/testing was performed during the light phase of the light–dark cycle.

### 6.13. Effort-Based Choice Behavior Procedures

Pharmacological agents: (*S*)-MK-26 was obtained from the Lubec Laboratory (University of Vienna, Austria) and dissolved in dimethyl sulfoxide (DMSO) (10%), Tween 80 (15%), and 0.9% saline (75%). The DMSO/Tween 80/saline mixture was administered as the vehicle control. The dose range was selected as 3.75–45.0 mg/kg based on its DAT affinity and pilot studies. It was administered 30 min before testing. TBZ (9,10-dimethoxy3-(2-methylpropyl)-1,3,4,6,7, 11b hexahydrobenzo[a]quinolizin2-one) was obtained from Tocris Bioscience (Ellisville, MO) and was dissolved in DMSO (20%) and 0.9% saline (80%) and was titrated with 1.0 N HCl until the drug was dissolved at a pH of 4.0–4.5. The DMSO/saline solution with equal amounts of HCl was administered as the vehicle control. The selected dose was 1.0mg/kg based on the previous research from our laboratory. It was administered 120 min prior to testing.

FR5/chow feeding choice task: A total of 8 adult male Sprague Dawley rats (Envigo Sprague Dawley, Indianapolis, IN, USA; weights 275–299 g upon arrival) were pair-housed in a colony maintained at 23 °C, with a 12 h light/dark cycle (lights on 07:00). Rats were food deprived to 85% of their free-feeding body weight with modest growth throughout the experiment and water was available ad libitum in their home cages. All the animal protocols were approved by the University of Connecticut Institutional Animal Care and Use Committee, and the experiments were completed according to National Institutes of Health guidelines.

Behavioral sessions were conducted in operant chambers (28 × 23 × 23 cm^3^; Med Associates, Fairfax, VT, USA) with 30 min sessions 5 days/week. Rats were initially trained to lever press on magazine training for 3 days, followed by an FR1 schedule for 3 days (high-carbohydrate 45-mg pellets, Bio-Serv, Frenchtown, NJ, USA), and then moved to the FR5 schedule. After 5 weeks of training on the FR5 schedule, lab chow was introduced as the concurrently available choice food (Laboratory Diet, 5P00 Prolab RMH 3000, Purina Mills, St. Louis, MO, USA; typically 15–20 g). At the end of each session, rats were immediately removed from the chambers. The number of lever pressing was recorded and the amount of consumed chow was calculated by weighing the remaining food, including spillage. Rats were trained on the FR5/chow feeding choice task for 5 weeks, after which drug testing began.

To test the effects of (*S*)-MK-26 on FR5/Chow feeding choice task, trained rats were administered either with TBZ (1.0 mg/kg) or vehicle 120 min before testing, and (*S*)-MK-26 also with doses of 15.0, 30.0, 45.0 mg/kg or vehicle 30 min before testing, via intraperitoneal (IP) injections on drug testing days. A repeated-measures design was utilized where each rat received each drug treatment in a randomly varied order, once per week. The following five treatment combinations were given: TBZ vehicle + (*S*)-MK-26 vehicle; 1.0 mg/kg TBZ + (*S*)-MK-26 vehicle; 1.0 mg/kg TBZ + 15.0 mg/kg (*S*)-MK-26; 1.0 mg/kg TBZ + 30.0 mg/kg (*S*)-MK-26; 1.0 mg/kg TBZ + 45.0 mg/kg (*S*)-MK-26. Next, to see if the effects of lower doses, the following four treatment combinations were given in a repeated measure design: TBZ vehicle + (*S*)-MK-26 vehicle; 1.0 mg/kg TBZ + (*S*)-MK-26 vehicle; 1.0 mg/kg TBZ + 3.75 mg/kg (*S*)-MK-26; 1.0 mg/kg TBZ + 7.5 mg/kg (*S*)-MK-26.

PROG/chow feeding choice task: A total of 15 adult male Sprague Dawley rats (Envigo Sprague Dawley, Indianapolis, IN, USA; weights 275–299 g upon arrival) were pair-housed in a colony maintained at 23 °C, with a 12 h light/dark cycle (lights on 07:00). Rats were food deprived to 85% of their free-feeding body weight with modest growth throughout the experiment and water was available ad libitum in their home cages. All the animal protocols were approved by the University of Connecticut Institutional Animal Care and Use Committee, and the experiments were completed according to National Institutes of Health guidelines.

Behavioral sessions were conducted in operant chambers (28 × 23 × 23 cm^3^; Med Associates, Fairfax, VT) with 30 min sessions 5 days/week. Rats were initially trained to lever press on magazine training for 3 days, followed by an FR1 schedule for 3 days (high-carbohydrate 45-mg pellets, Bio-Serv, Frenchtown, NJ, USA), and then moved to the PROG schedule [137,138]. During PROG sessions, the ratio started at FR1 and was increased by one additional response every time 15 reinforcements were obtained (FR1 × 15, FR2 × 15, etc.). If 2 min have passed without completing a ratio, a “time-out” feature deactivated the response lever for the rest of the session. After 9 weeks of training on the PROG schedule, lab chow was introduced as the concurrently available choice food (Laboratory Diet, 5P00 Prolab RMH 3000, Purina Mills, St. Louis, MO, USA; typically, 15–20 g). At the end of each session, rats were immediately removed from the chambers. The number of lever pressing was recorded and the amount of consumed chow was calculated by weighing the remaining food, including spillage. Rats were trained on the PROG/chow feeding choice task for 5 weeks, after which drug testing began.

Animals were trained in PROG/Chow Feeding Choice Task was utilized to test the effect of (*S*)-MK-26 on the PROG/chow feeding choice task when administered alone using a repeated measure design. Rats were administered either vehicle or 15.0, 30.0, or 45.0 mg/kg doses of (*S*)-MK-26, 30 min before testing, via IP injections on drug testing days.

### 6.14. Statistical Analysis

GraphPad Prism (GraphPad Software, San Diego, CA, USA), SPSS Statistics v28 (IBM), and STATISTICA 12 (Statsoft; Tulsa, OK, USA) software packages were used for the statistical analysis. To analyze the effort-based choice behavior procedures, the total number of lever presses and chow intake from the 30 min sessions were evaluated using repeated-measures ANOVA. For all the significant overall F values, nonorthogonal planned comparisons were performed, using the overall error term to assess differences between each treatment and the vehicle condition. The number of comparisons was limited to the number of treatments minus one ([194]). If the lever presses or chow intake were not normally distributed according to the Shapiro–Wilk Normality test, a non-parametric test for related samples, Friedman’s two-way analysis of variance by ranks, was utilized. Data from ex vivo electrophysiology were analyzed by a two-way ANOVA with repeated-measures (RM) followed by Sidak post hoc test. For in vivo microdialysis studies, statistical analyses were performed with two-way RM-ANOVAs using treatment as the between-subjects factor and time (i.e., dialysate fractions) as the within-subjects factor. Before performing ANOVA, raw data were percent transformed (with 100% as the average of the last four dopamine basal values before treatment) and data sets inspected for normal distribution with the Shapiro–Wilk’s test and for homogeneity of variances among the experimental groups with the Levene’s test. When significant differences in the variances of a data set were found, data were analyzed with ANOVAs and Geisser-Greenhouse correction. Moreover, an overall analysis of dopamine concentration data obtained from each rat during the microdialysis experiment was conducted by calculating the area under the curve (AUC) obtained by plotting the values of the concentrations of dopamine vs. time with the classical trapezoidal rule and then comparing the obtained values by two-way ANOVA with the treatment and brain area as between-subjects factors. When ANOVA revealed statistically significant main effects or interactions, pairwise comparisons were performed by using Bonferroni’s corrected paired *t* tests or the Tukey’s multicomparison test, respectively. Statistical analyses were carried out with the software STATISTICA 12 (Statsoft; Tulsa, OK, USA) with the significance level set at *p* < 0.05. For the hole-board maze test, RMIs were assessed separately each day as they inform about different phases of the learning task. Day-1 and Day-2 and Day-3 were examined by two-way repeated-measures ANOVA followed by Sidak’s test with multiple comparisons; Day-4 was evaluated by unpaired *t* test for two groups comparisons. Data are presented as Means ± standard error of the mean (SEM).

## 7. Conclusions

Here, we report the synthesis and pharmacological characterization of (*S*)-MK-26, a novel modafinil analogue with improved selectivity and increased inhibitory potency to DAT. In experiments using rats as the animal model, acute (*S*)-MK-26 treatment significantly increased synaptic plasticity and enhanced the transsynaptic levels of DA in both the prefrontal cortex and nucleus accumbens. Additionally, (*S*)-MK-26 reversed the effort-related motivational dysfunctions induced by TBZ treatment and increased the selection of high-effort choices. The capability of (*S*)-MK-26 to restore motivational deficiencies that resemble those typical of depression and other neurological disorders in humans suggests its potential for therapeutic use in the experimental and applied biomedical research fields.

## Figures and Tables

**Table 1:** Pharmacokinetic parameters of (*S*)-MK-26 upon i.p. administration with dose of 10 mg/kg of body weight.

Matrix	T_max_ (h)	C_max_ (ng/mL)	C_max_ (nM)	T_last_ (h)	C_last_ (nM)	AUC_last_ (h*ng/mL)	AUC_last_ (h*nM)
brain	0.25	634	1665	10.0	19.2	1959	5143
plasma	0.50	624	1637	10.0	15.7	1713	4497

**Table 2:** Overall assessment of the effect of (*S*)-MK-26 on DA concentrations in the NAc shell, NAc core and mPFC dialysate, as measured by the AUCs calculated from the data shown in Figure 4 and Figure 5. AUCs values are expressed as mean ± SEM. One-way ANOVA followed by Bonferroni’s multicomparison test. *: *p* < 0.05 vs. vehicle-treated rats; #: *p* < 0.05 vs. 1 mg/kg-treated rats.

DA Concentrations (as Determined from AUCs)				
	Treatment	Vehicle (Kolliphor 30%) 1.5 mL/kg	(*S*)-MK-26 1 mg/kg	(*S*)-MK-26 10 mg/kg
Brain Area				
NAc shell		664 ± 525	2972 ± 1094	7730 ± 1389 *#
NAc core		276 ± 104	3056 ± 644	7861 ± 1697 *#
mPFC		530 ± 129	3159 ± 223	4983 ± 547 *

## Data Availability

All the data derived and/or analyzed during this research study are included in this article.

## Supplementary Materials

The following supporting information can be downloaded at: https://www.mdpi.com/article/10.3390/biom12070881/s1, ^1^H, ^13^C and 2D NMR spectra (Page 2–62), high-resolution mass spectrometry data (Page 63–70), HPLC-determined purity data (Page 71–76), VCD report (Page 77–85), binding study (Page 86–97), and molecular formula strings (uploaded as CSV file).

## Abbreviations

DA	Dopamine
DAT	Dopamine Transporter
hDAT	human dopamine Transporter
hNET	human norepinephrine transporter
hSERT	human serotonin transporter
DIPEA	potassium tert-butoxide
BF_3_·Et_2_O	boron trifluoride diethyl etherate
[^3^H] MPP^+^	methyl-4-phenylpyridinium
KHB	Krebs–Henseleit Buffer
PDL	Poly-D-Lysine
CDCl_3_	deuterated chloroform
DMSO-d_6_	deuterated dimethyl sulfoxide
i.p.	intraperitoneal
LTP	long-term potentiation
PTP	post-tetanic potentiation
I/O	input/output
