# Supplementary material for: A Novel and Selective Dopamine Transporter Inhibitor, (S)-MK-26, Promotes Hippocampal Synaptic Plasticity and Restores Effort-Related Motivational Dysfunctions
Source: Biomolecules. 2022 Jun 24;12(7):881. doi: 10.3390/biom12070881 (PMC9312958; doi:10.3390/biom12070881)
Supplement: Supplementary file 1 [file biomolecules-12-00881-s001.zip › biomolecules-1744521-supplementary.pdf]

Supplementary material

1. Chirally resolved *(S)*-MK-26 after racemic synthesis

1.1. ^{1}H and ^{13}C NMR spectra

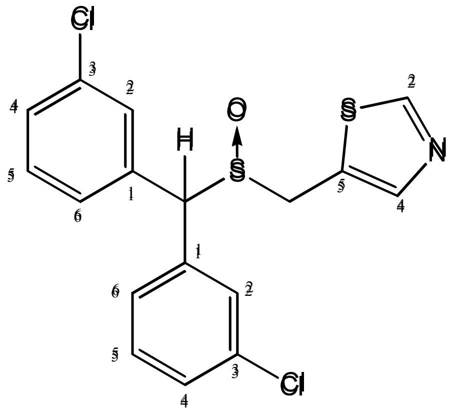

**MK026p1** in $CDCl_3$		^{1}H	^{13}C
Thia 2	CH	8,88	155,16
Thia 4	CH	7,71	144,29
Thia 5	C	--	124,51
Ph 1	C	--	136,20
Ph 1'	C	--	135,36
Ph 2	CH	7,36	128,67
Ph 2'	CH	7,34	129,32
Ph 3	C	--	135,62
Ph 3'	C	--	134,85
Ph 4	CH	7,41	130,93
Ph 4'	CH	7,33	130,14
Ph 5	CH	7,40	129,29
Ph 5'	CH	7,34	129,06
Ph 6	CH	7,27	127,60
Ph 6'	CH	7,28	126,70
CH	CH	4,57	68,55
CH_2	CH_2	4,17/3,92	46,96

MK026p1 in cdcl3 (Proton) 4.11.2019

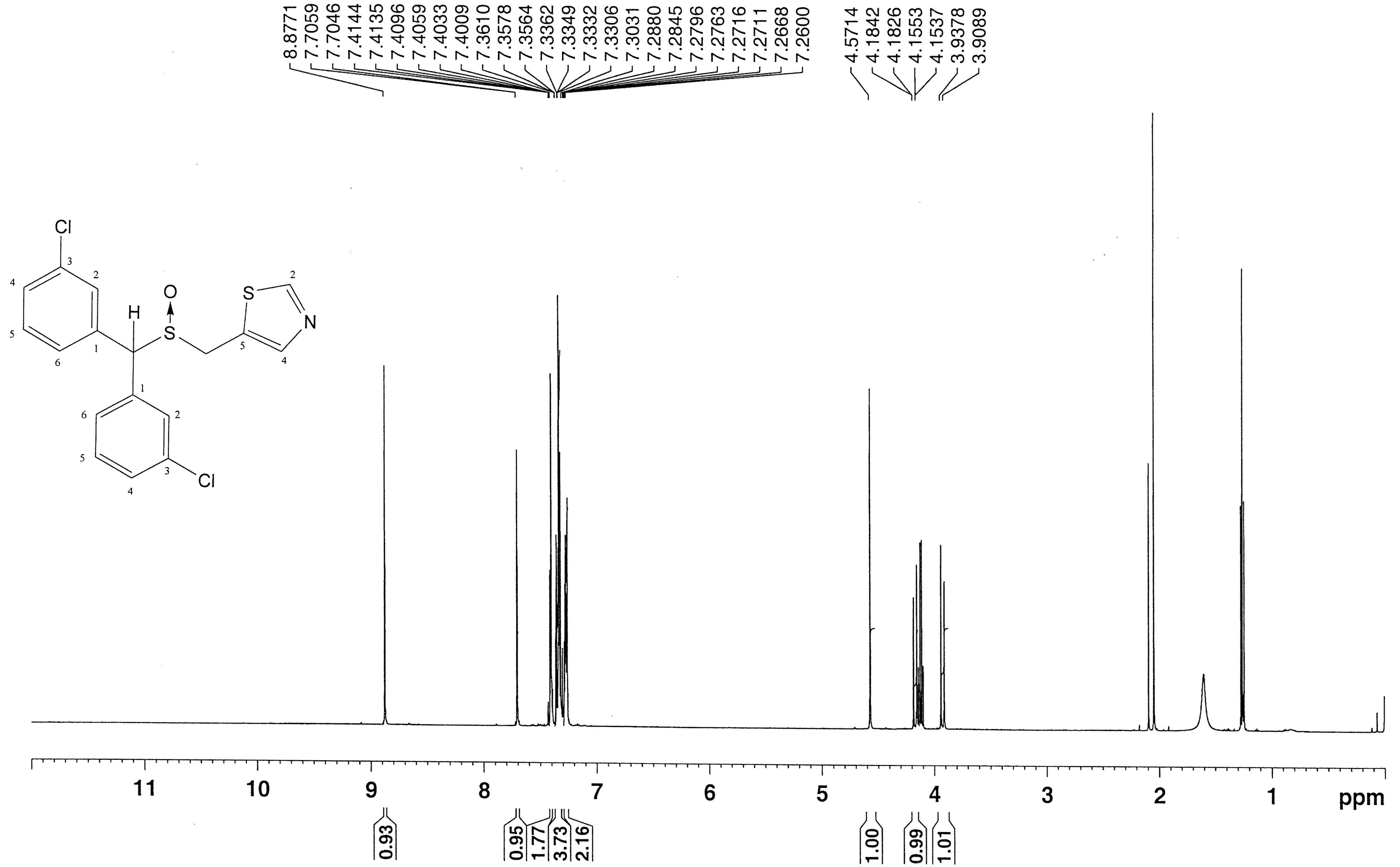

MK026p1 in cdcl3 (Proton) 4.11.2019

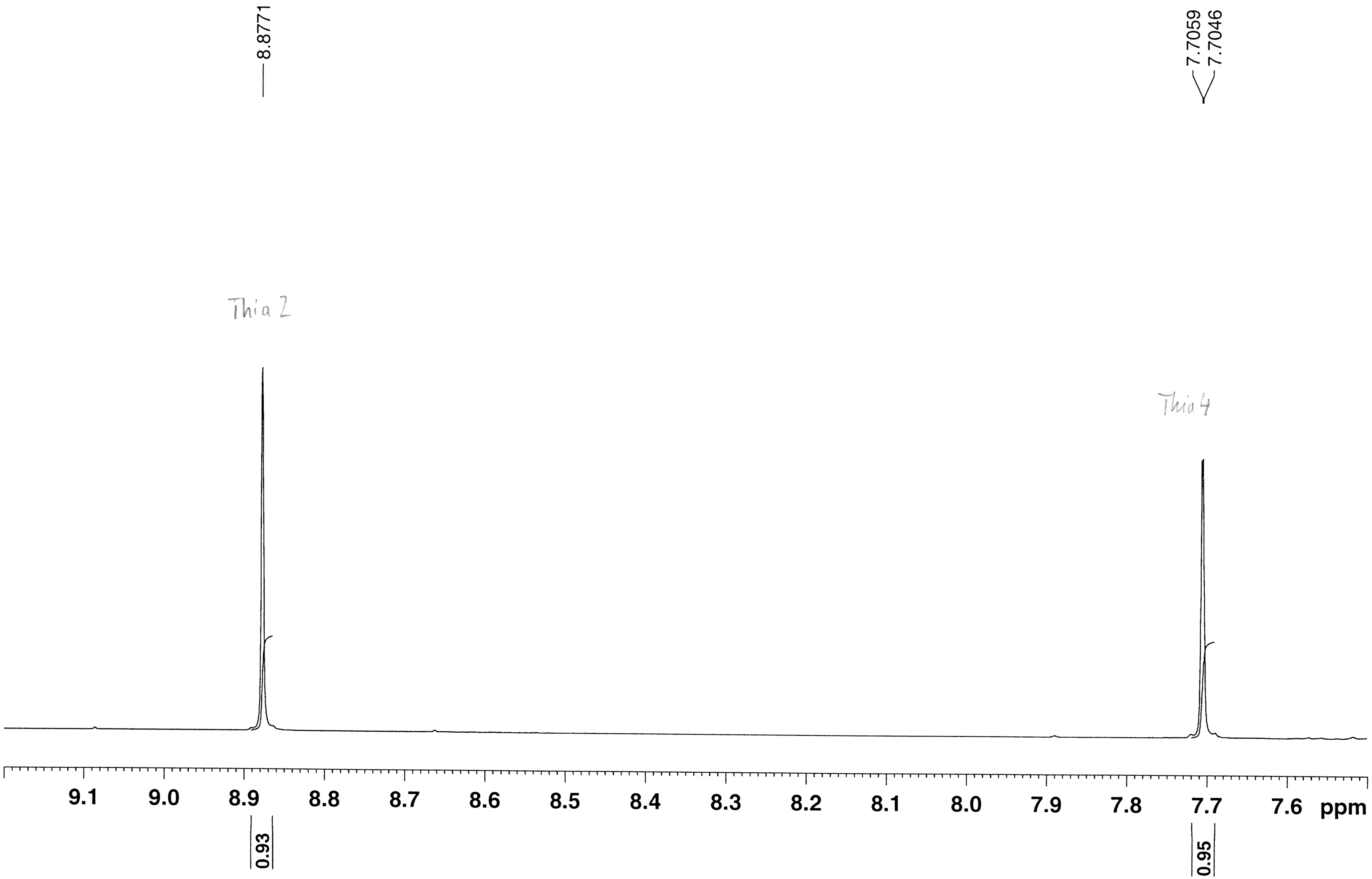

MK026p1 in cdcl3 (Proton) 4.11.2019

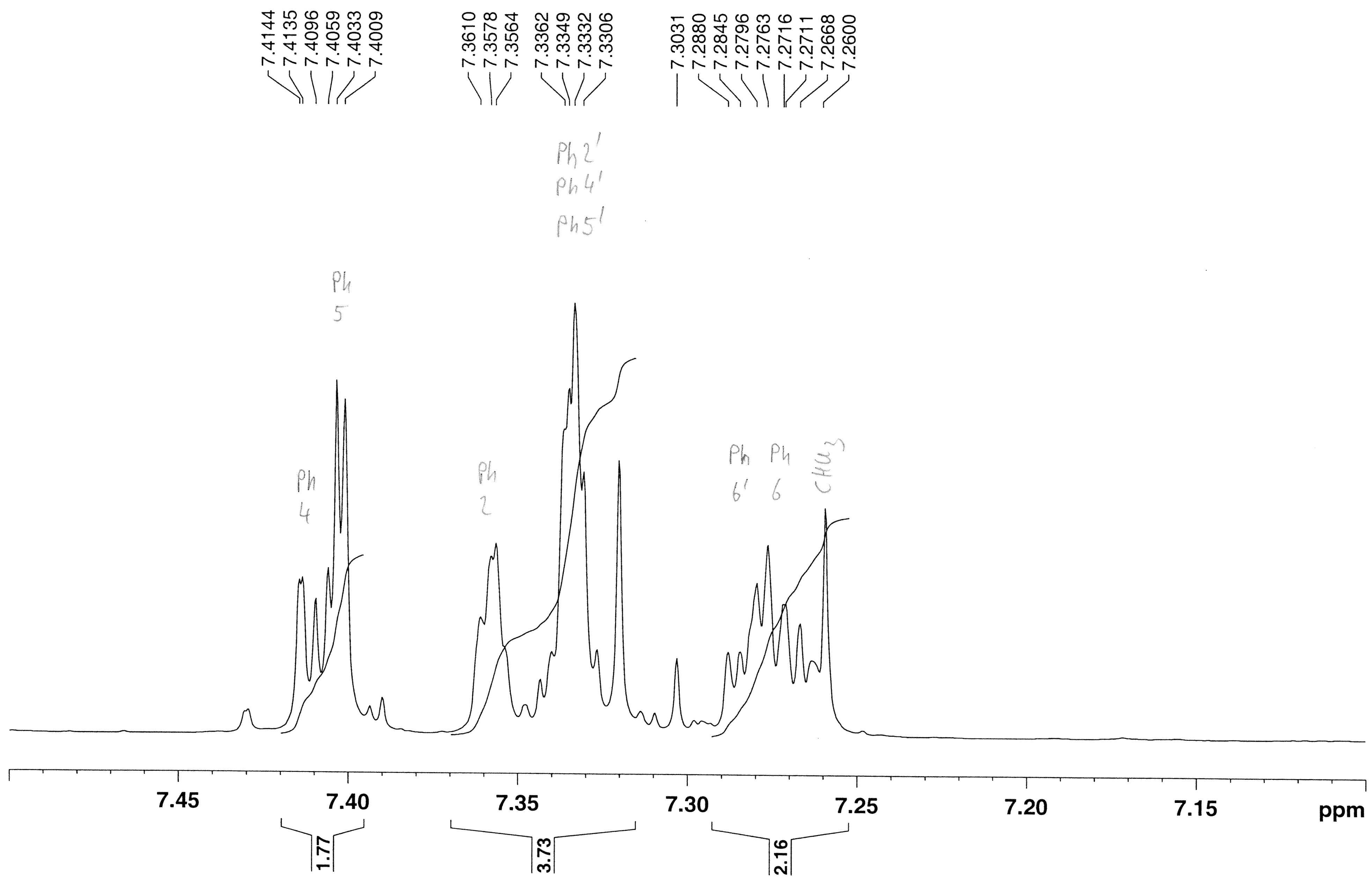

MK026p1 in cdcl3 (Proton) 4.11.2019

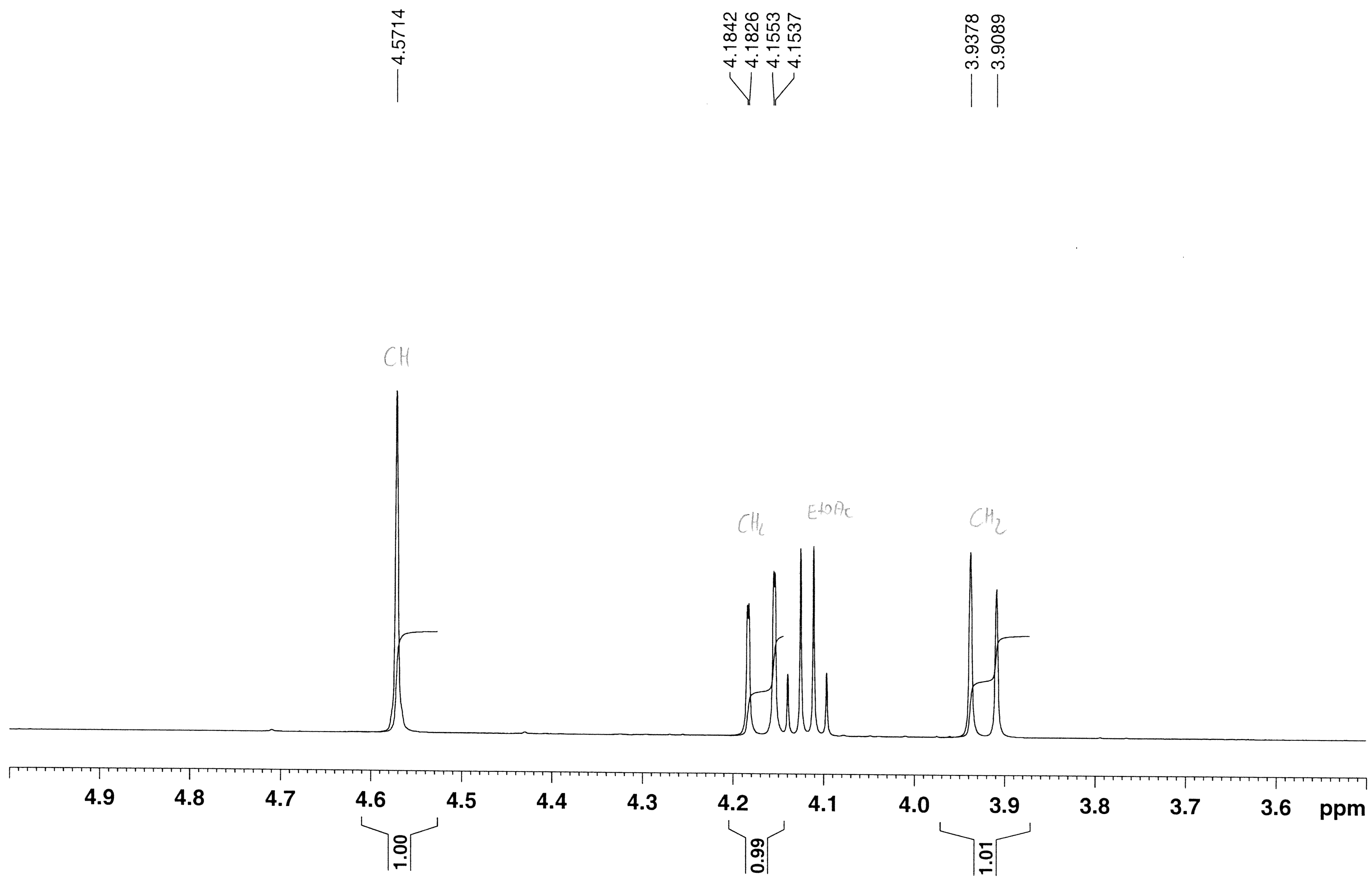

MK026p1 in cdcl3 (APT) 4.11.2019

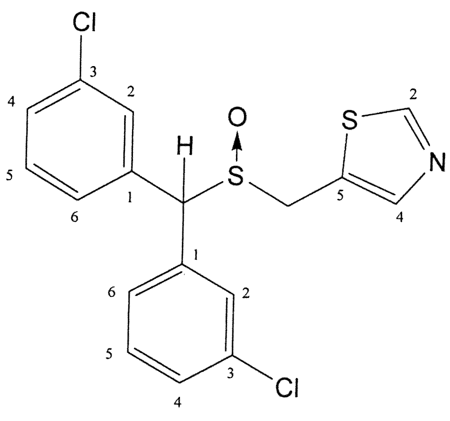

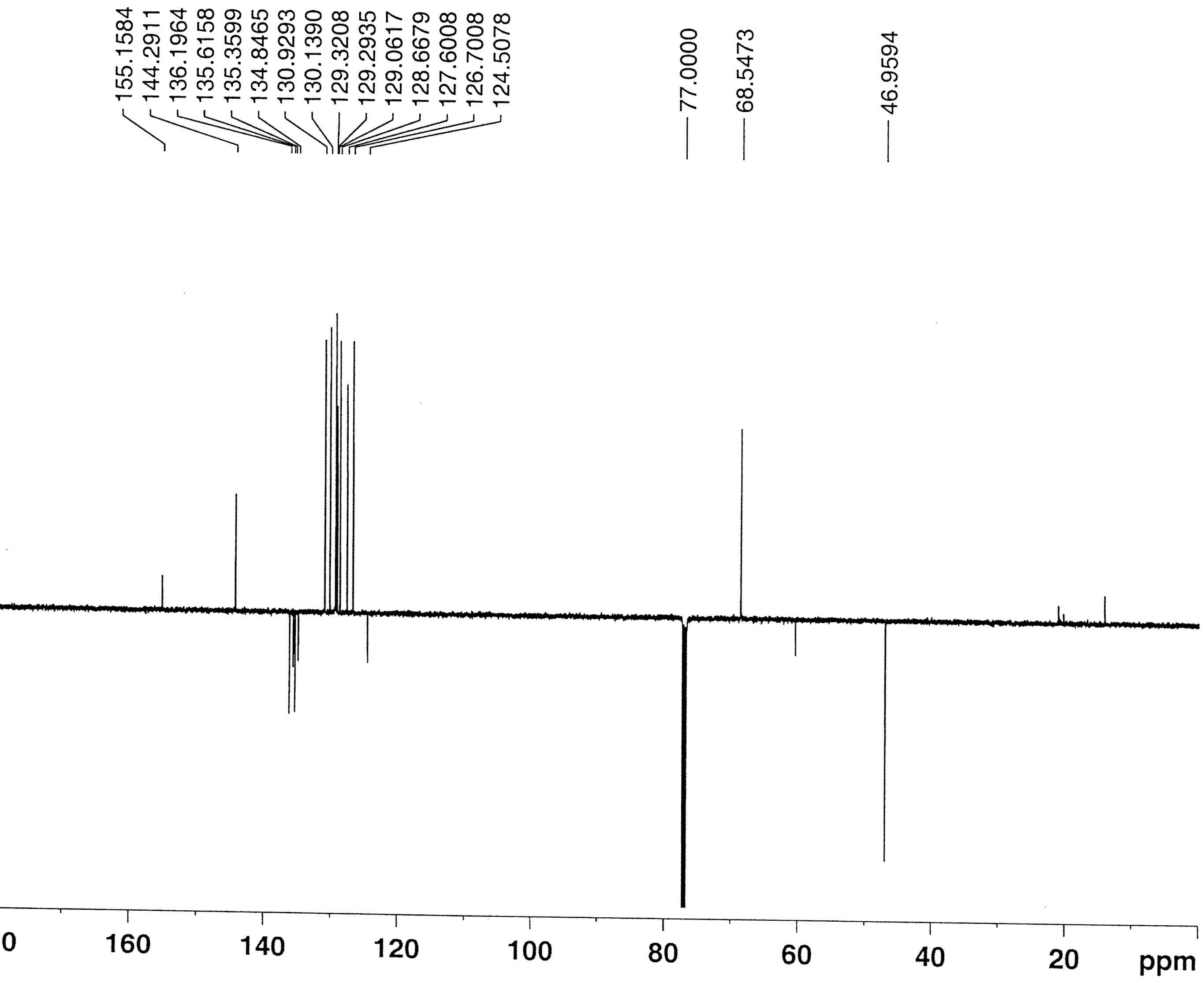

MK026p1 in cdcl3 (APT) 4.11.2019

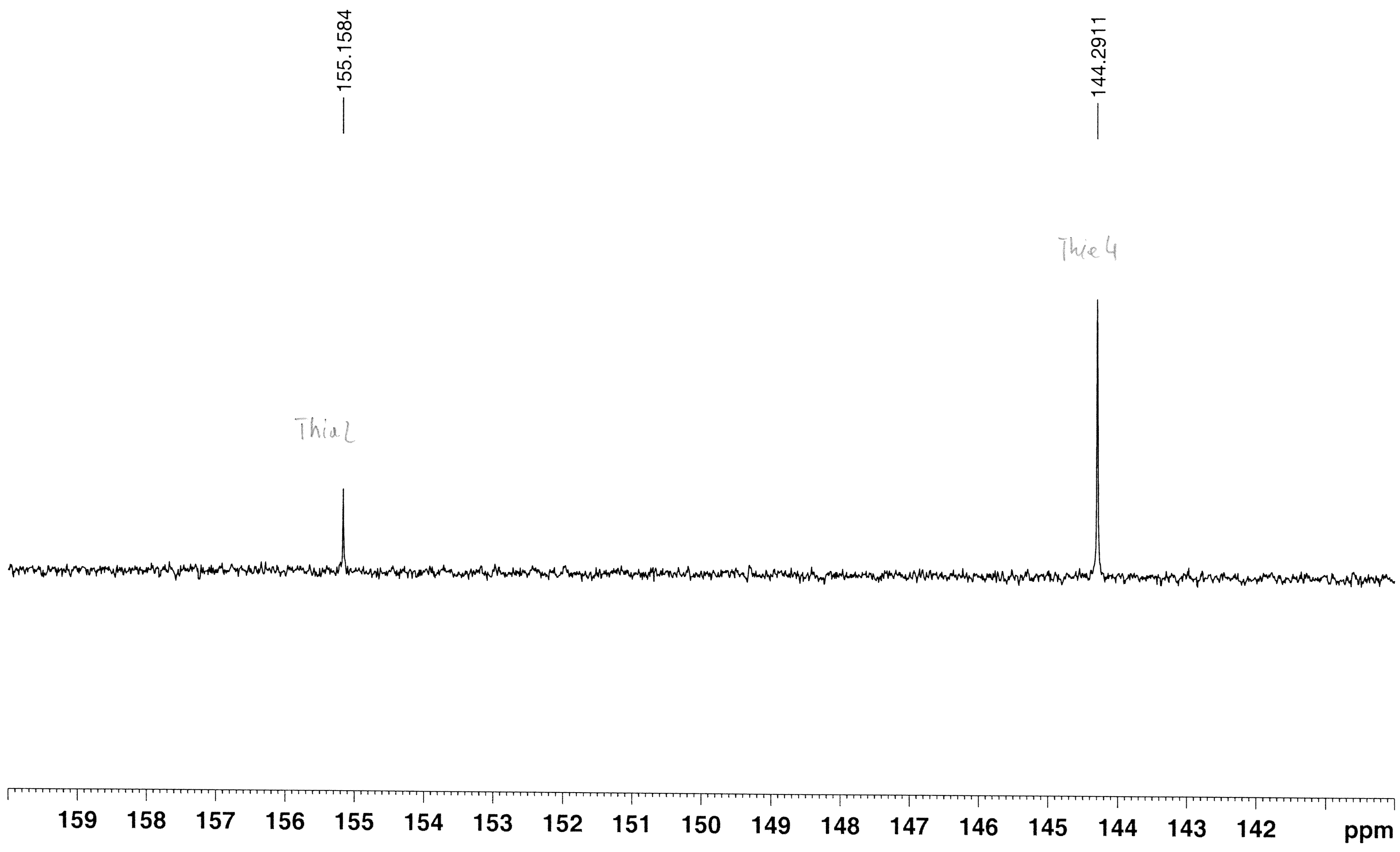

MK026p1 in cdcl3 (APT) 4.11.2019

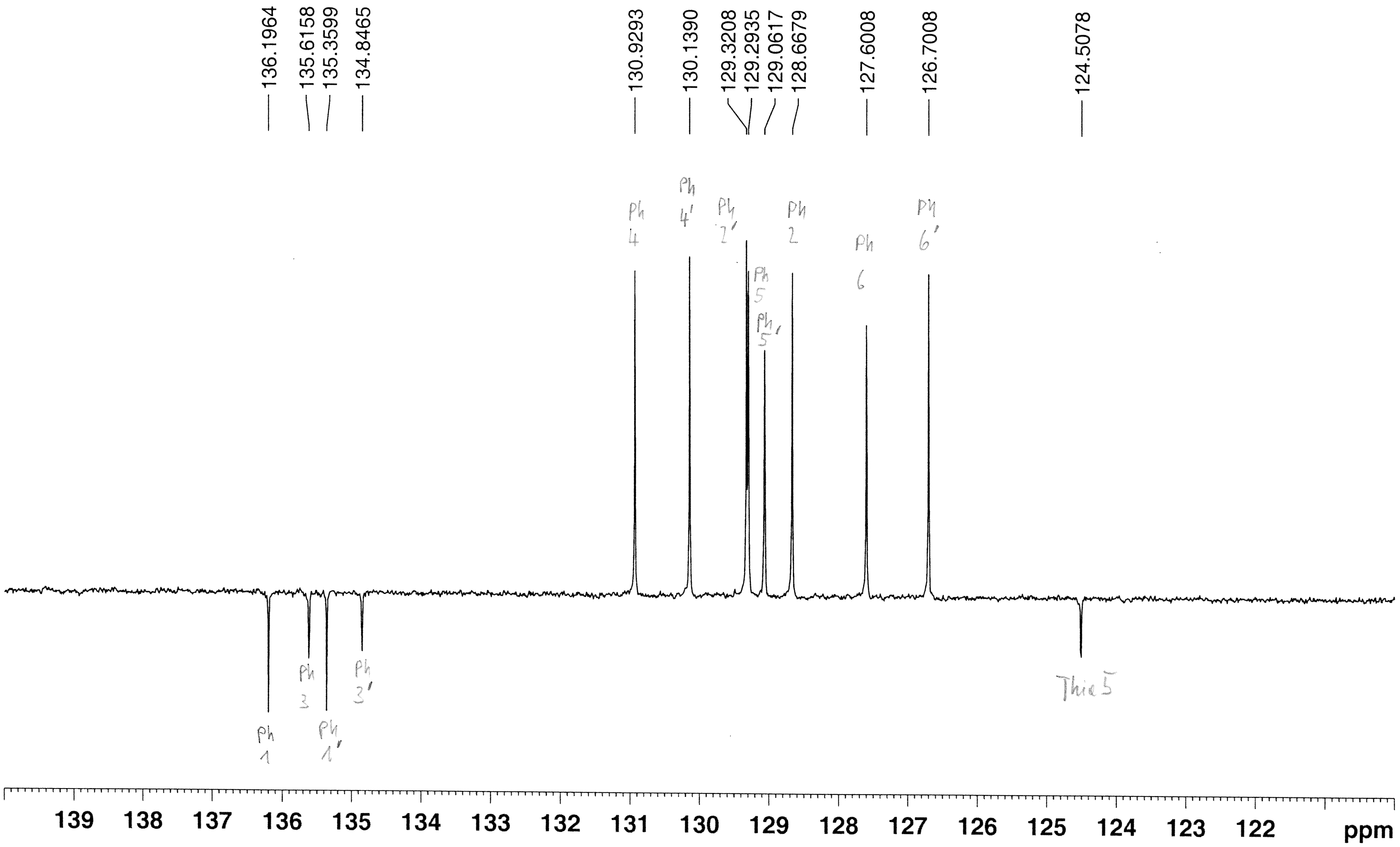

MK026p1 in cdcl3 (APT) 4.11.2019

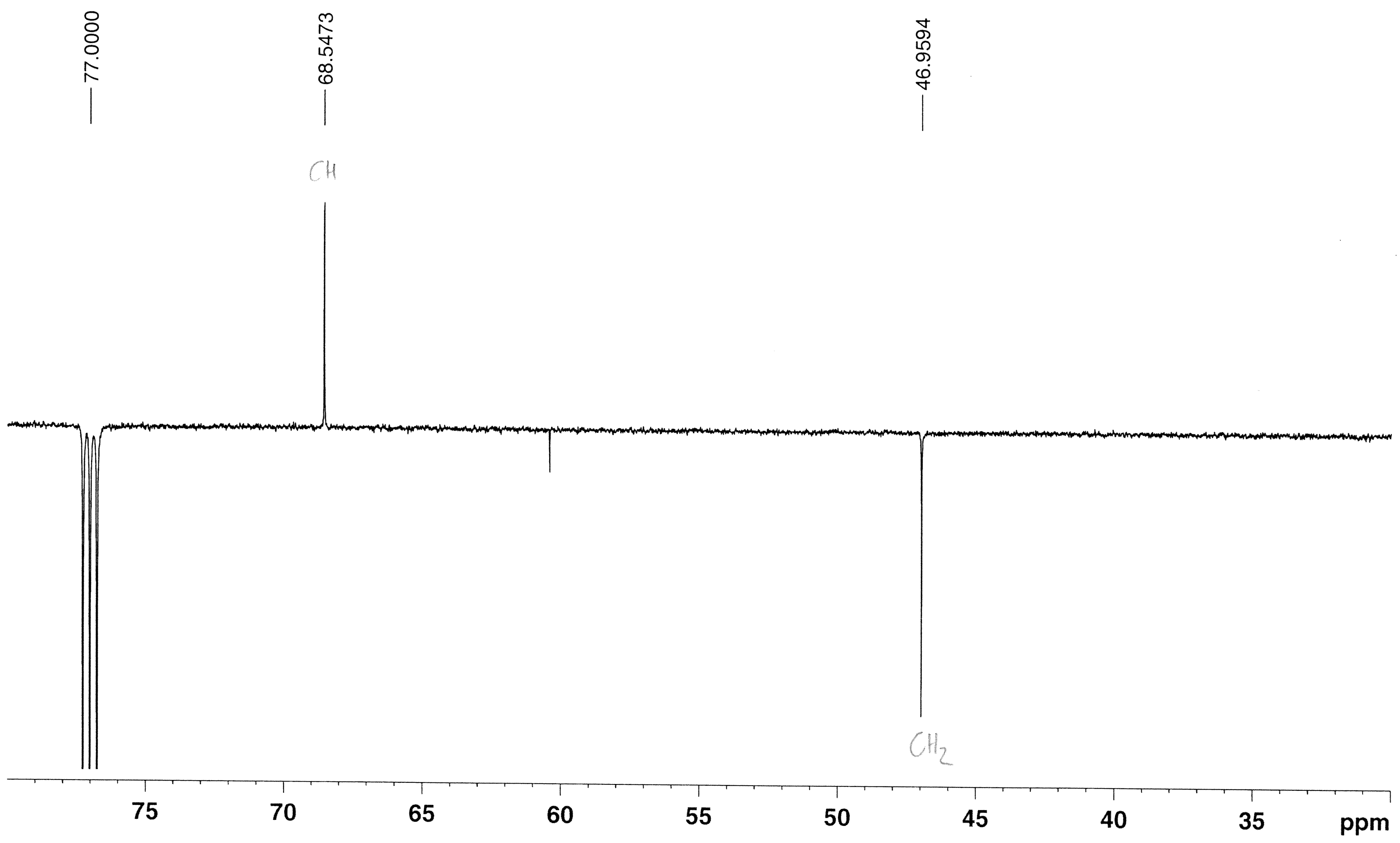

MK026p1 in cdcl3 (COSY) 4.11.2019

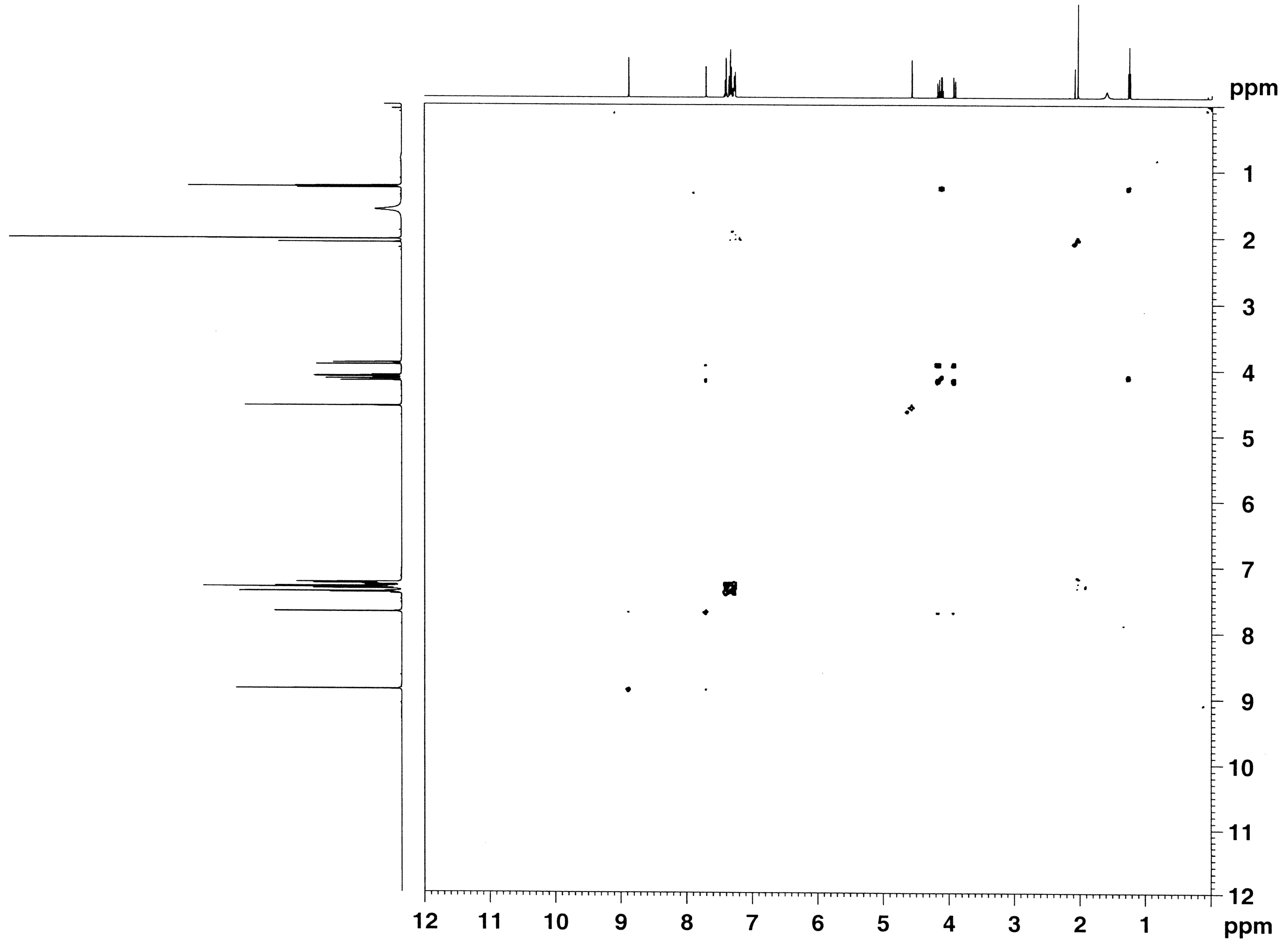

MK026p1 in cdcl3 (COSY) 4.11.2019

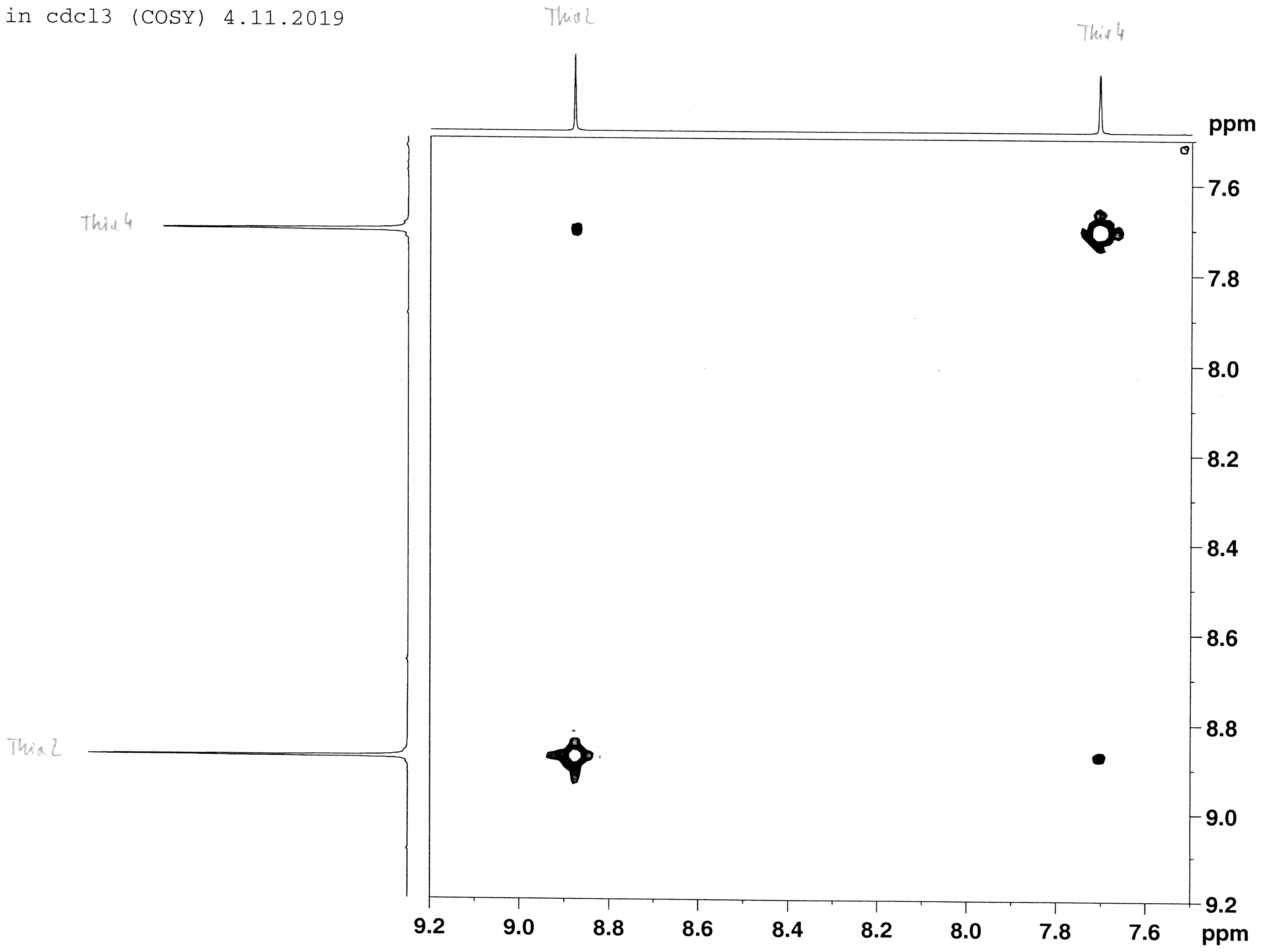

MK026p1 in cdcl3 (COSY) 4.11.2019

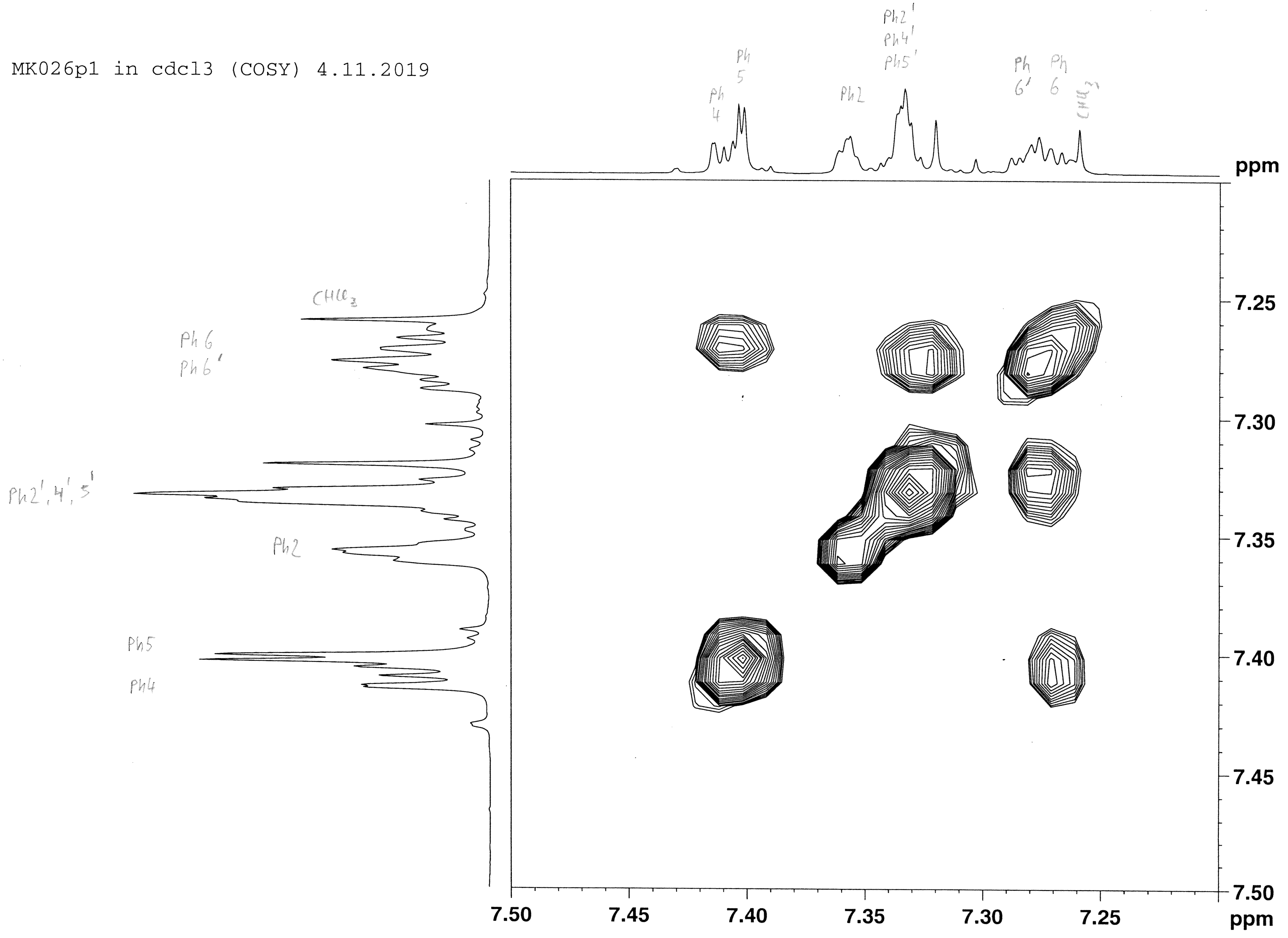

MK026p1 in cdcl3 (COSY) 4.11.2019

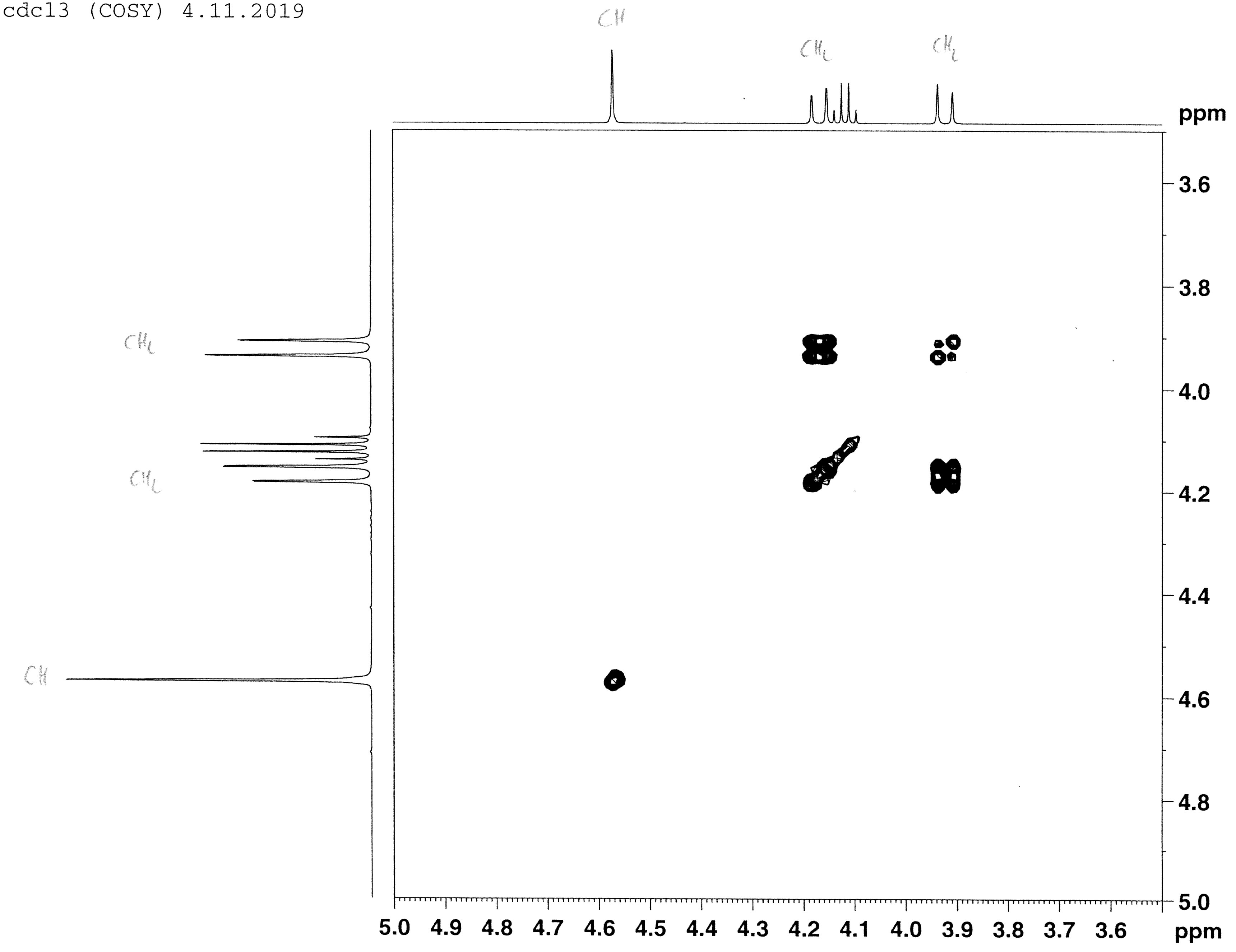

MK026p1 in cdcl3 (HSQC) 4.11.2019

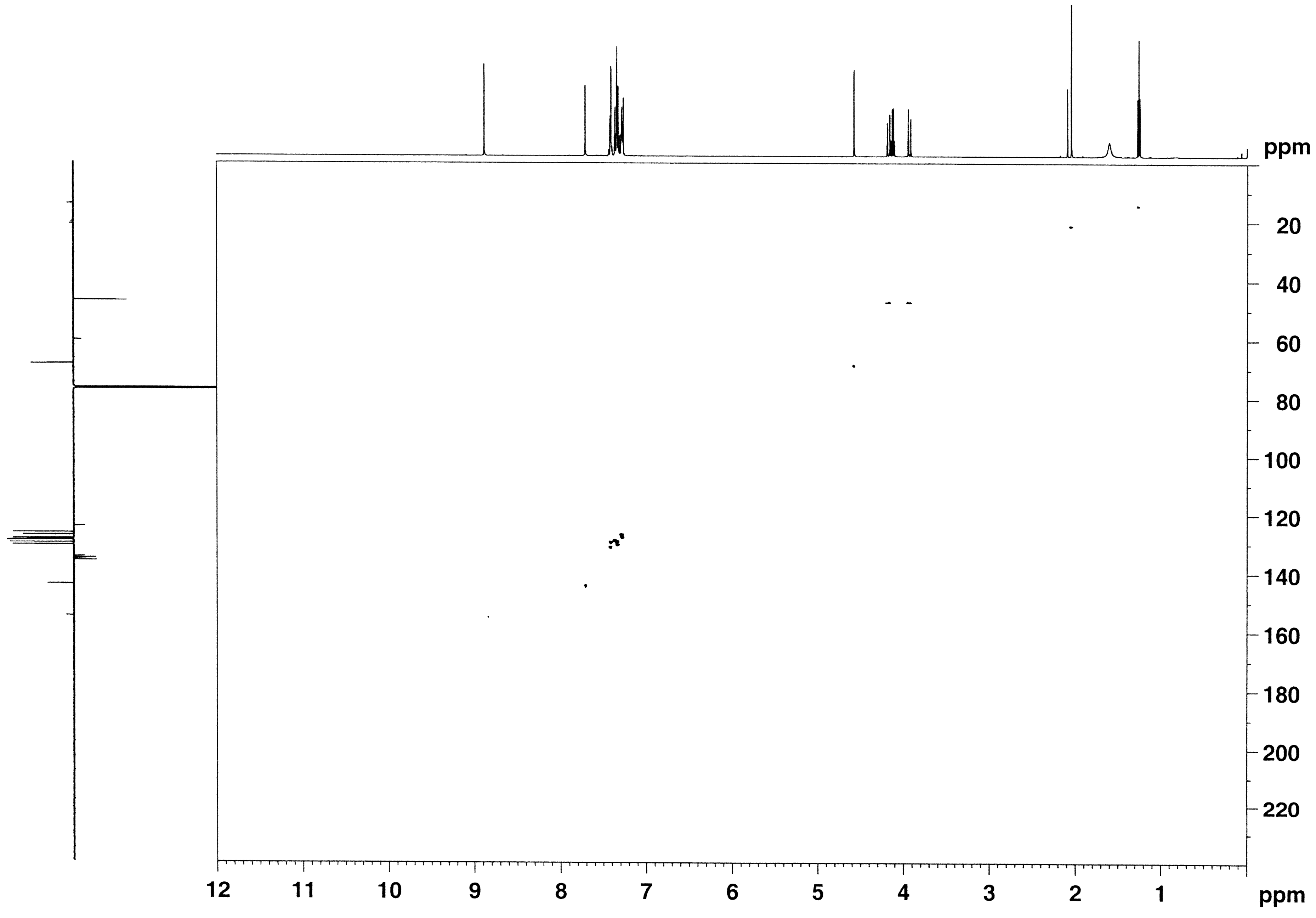

MK026p1 in cdcl3 (HSQC) 4.11.2019

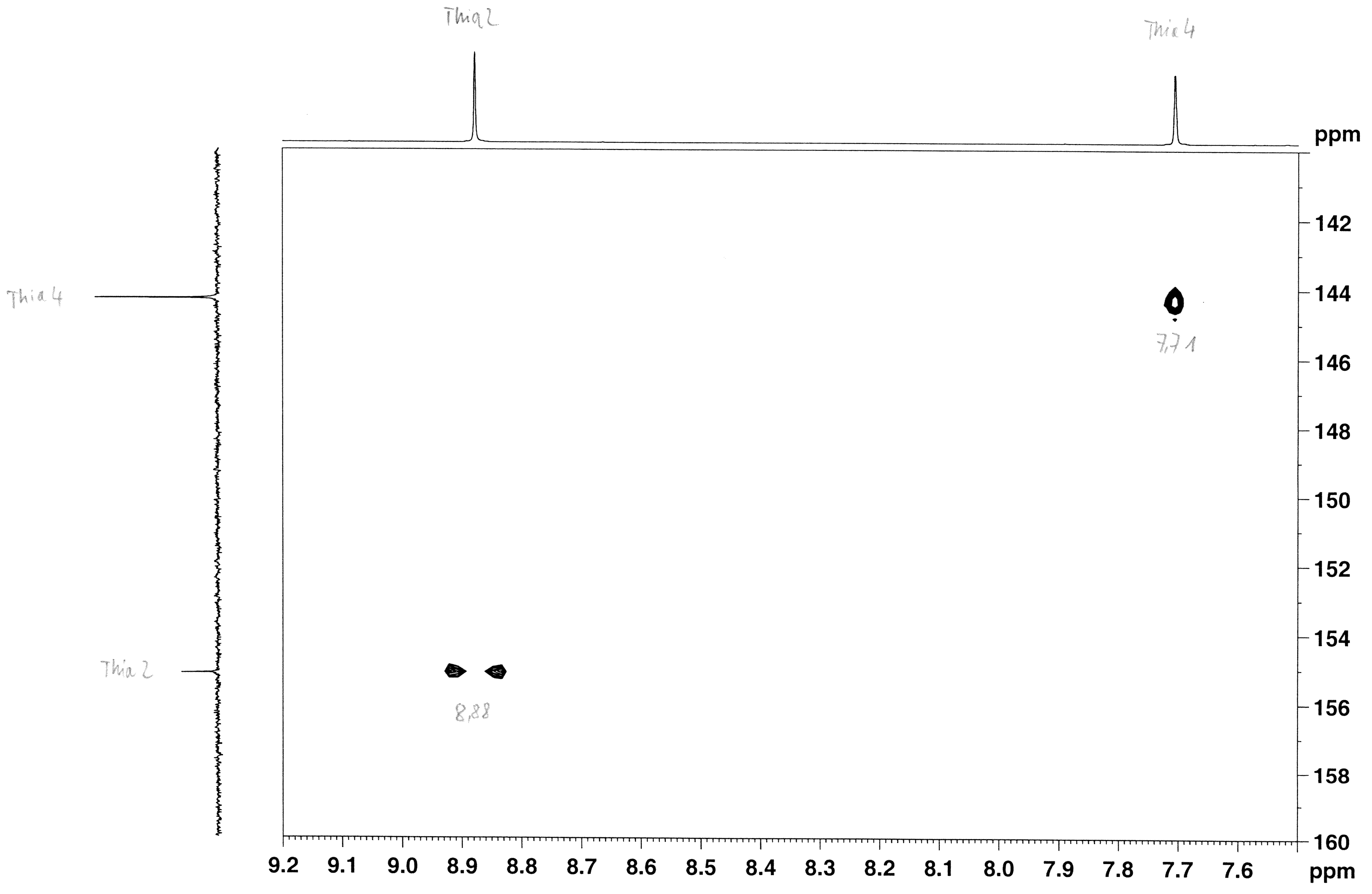

MK026p1 in cdcl3 (HSQC) 4.11.2019

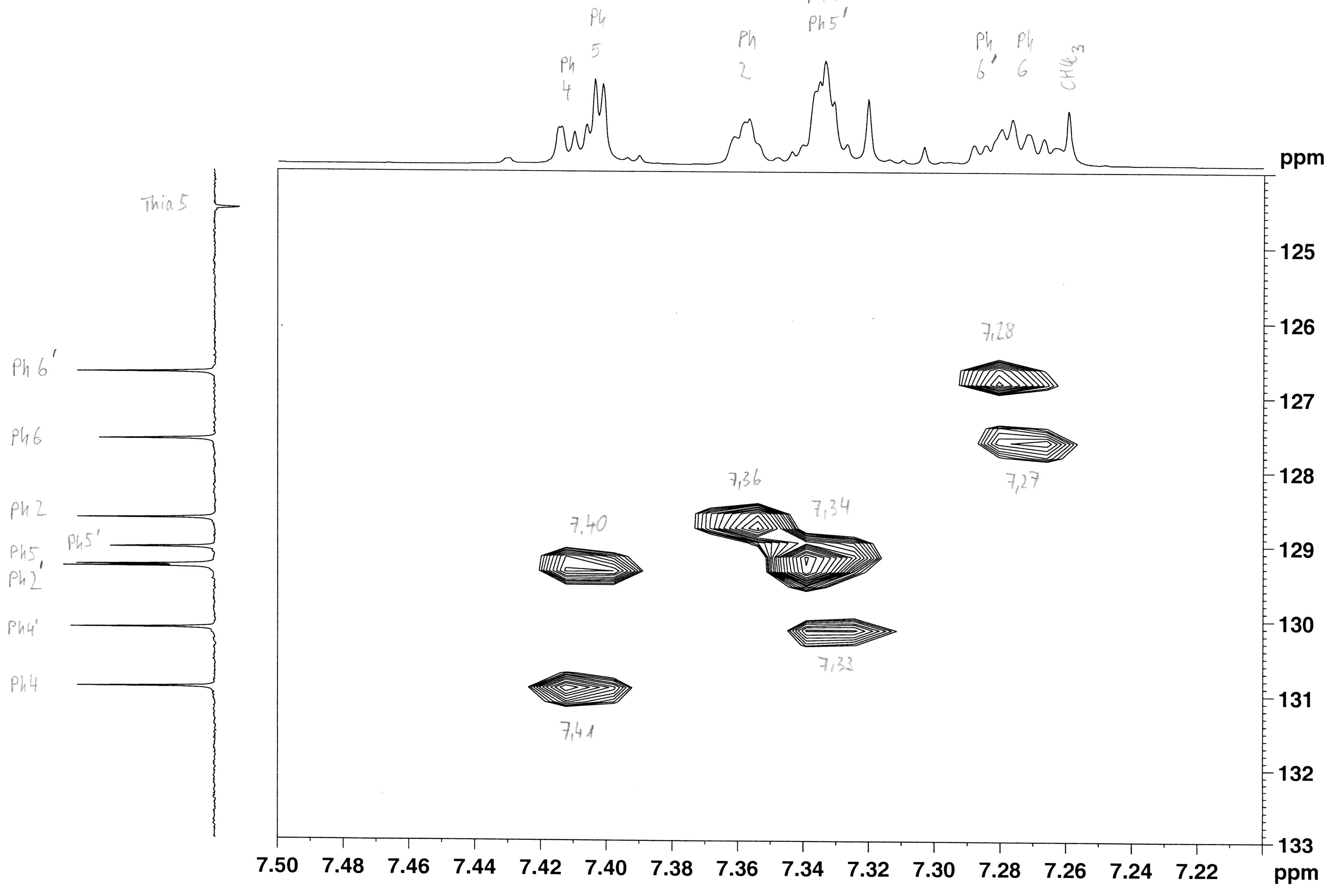

MK026p1 in cdcl3 (HSQC) 4.11.2019

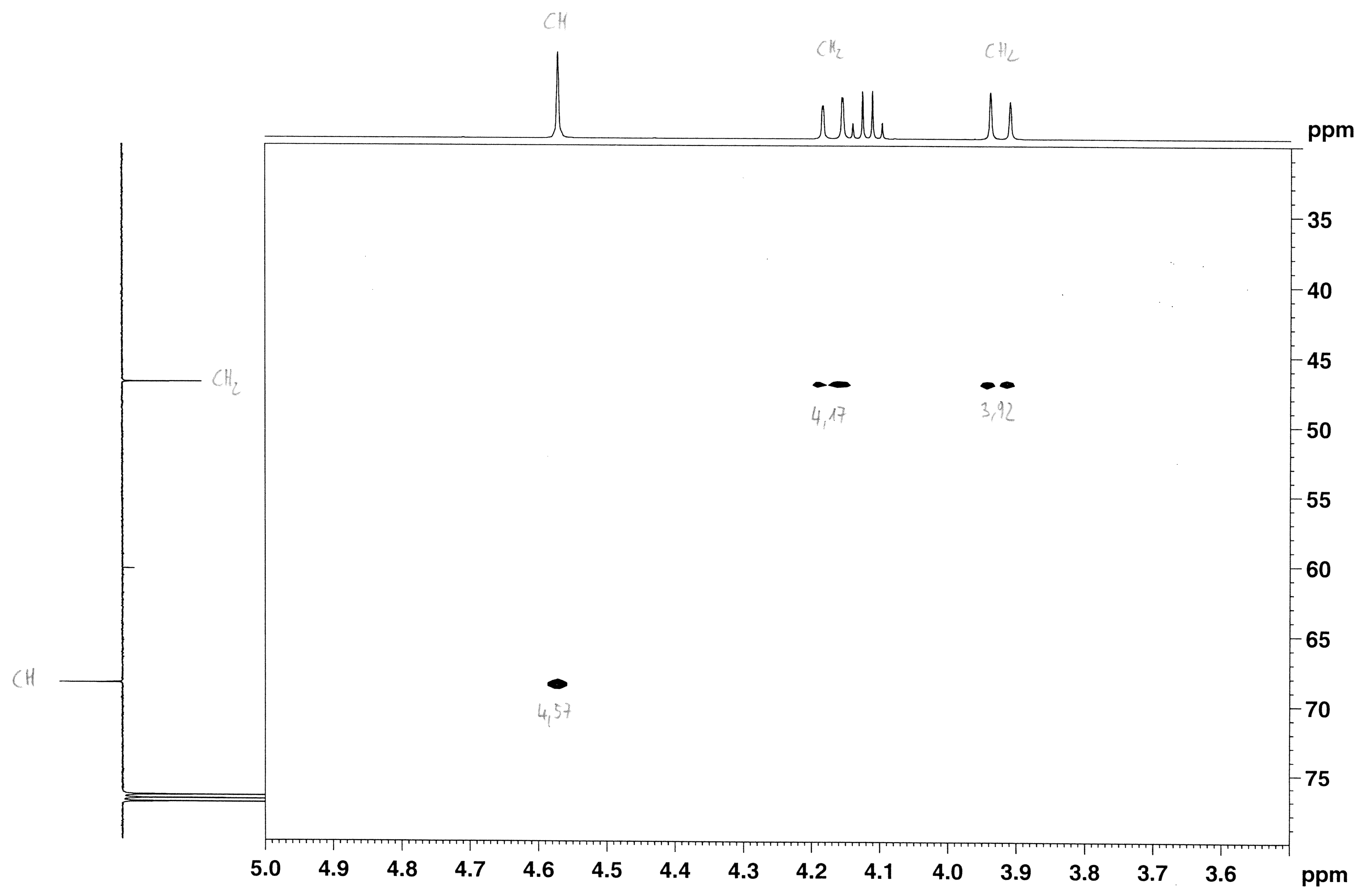

MK026p1 in cdcl3 (HMBC) 4.11.2019

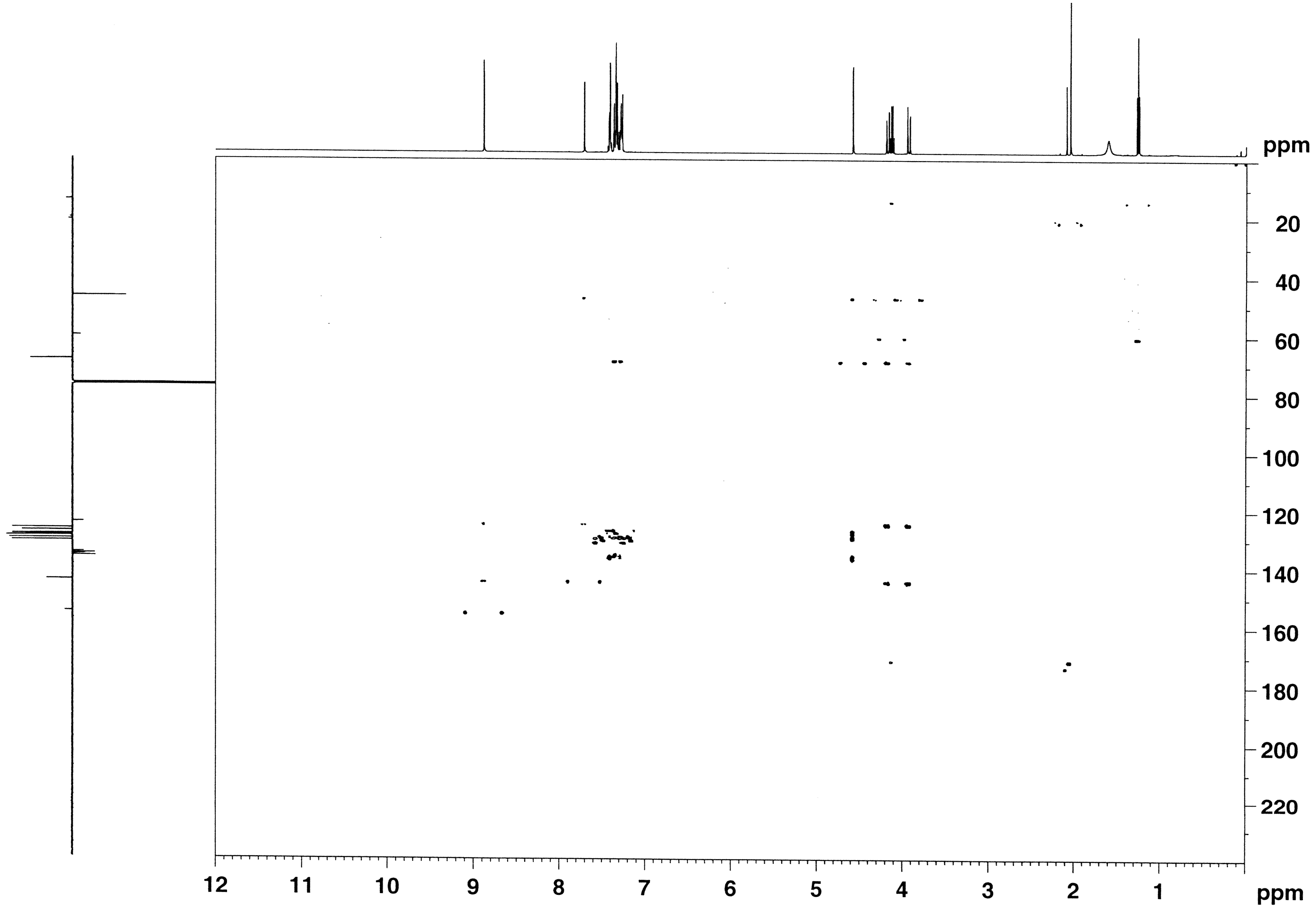

MK026p1 in cdcl3 (HMBC) 4.11.2019

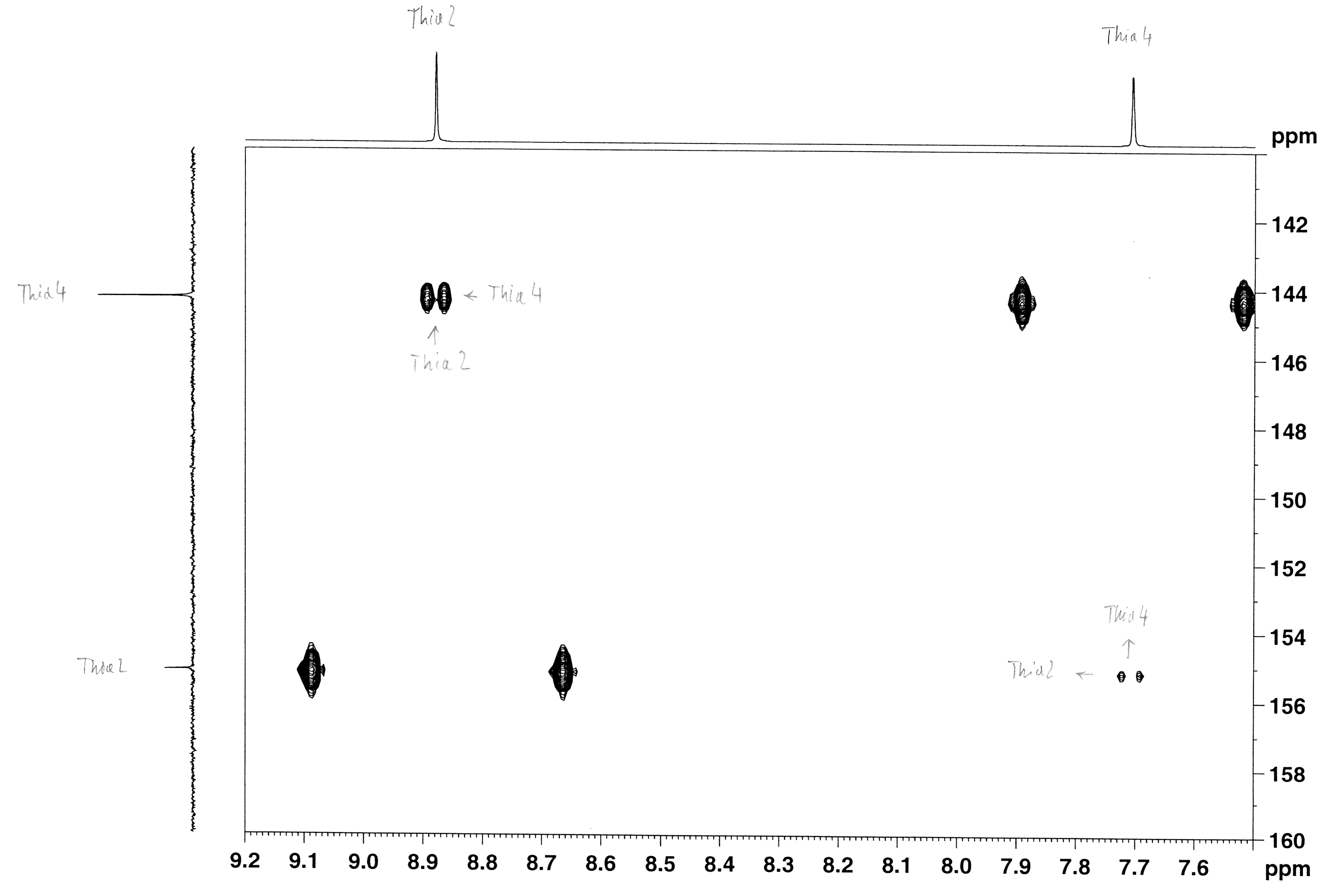

MK026p1 in cdcl3 (HMBC) 4.11.2019

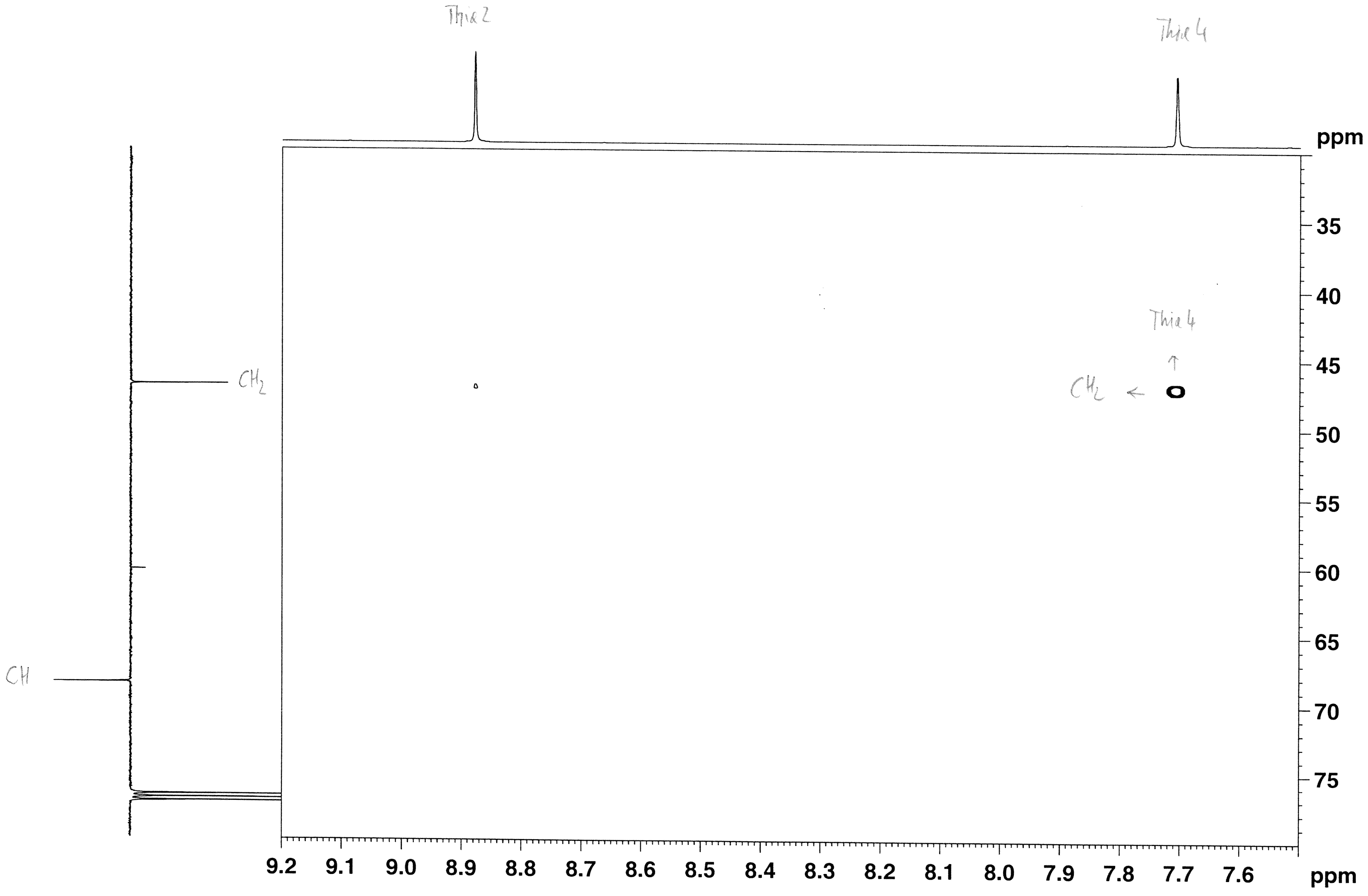

MK026p1 in cdcl3 (HMBC) 4.11.2019

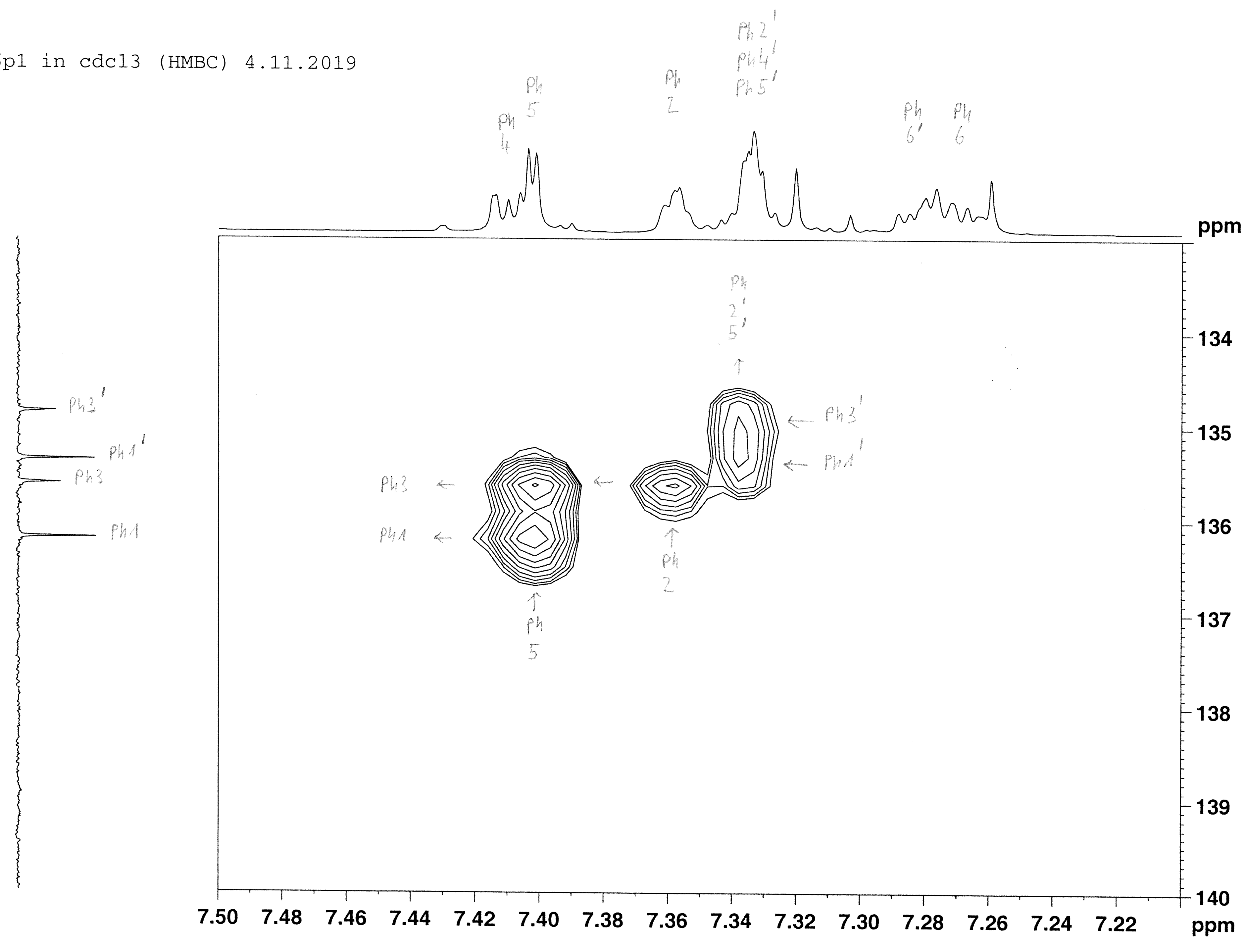

MK026p1 in cdcl3 (HMBC) 4.11.2019

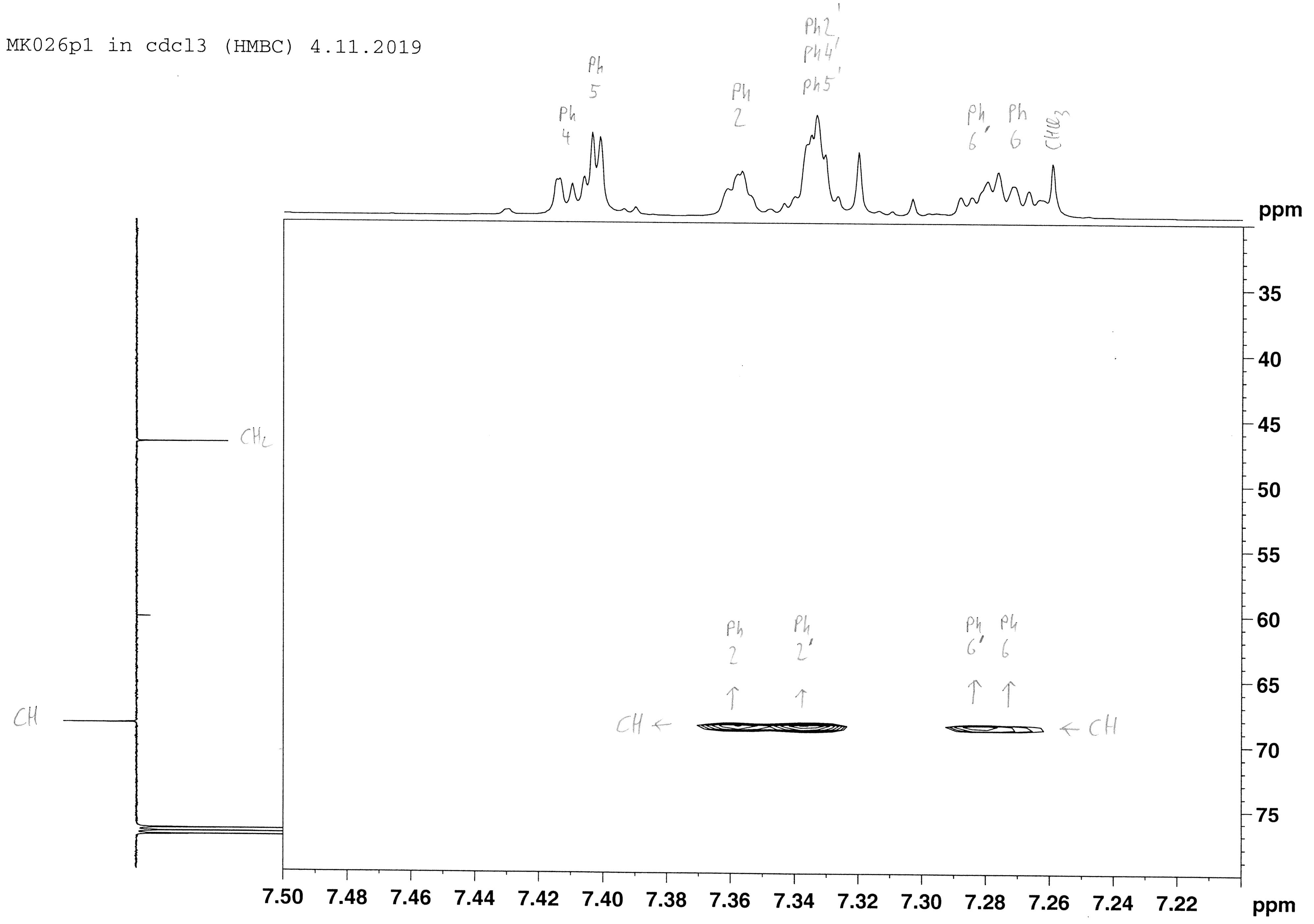

MK026p1 in cdcl3 (HMBC) 4.11.2019

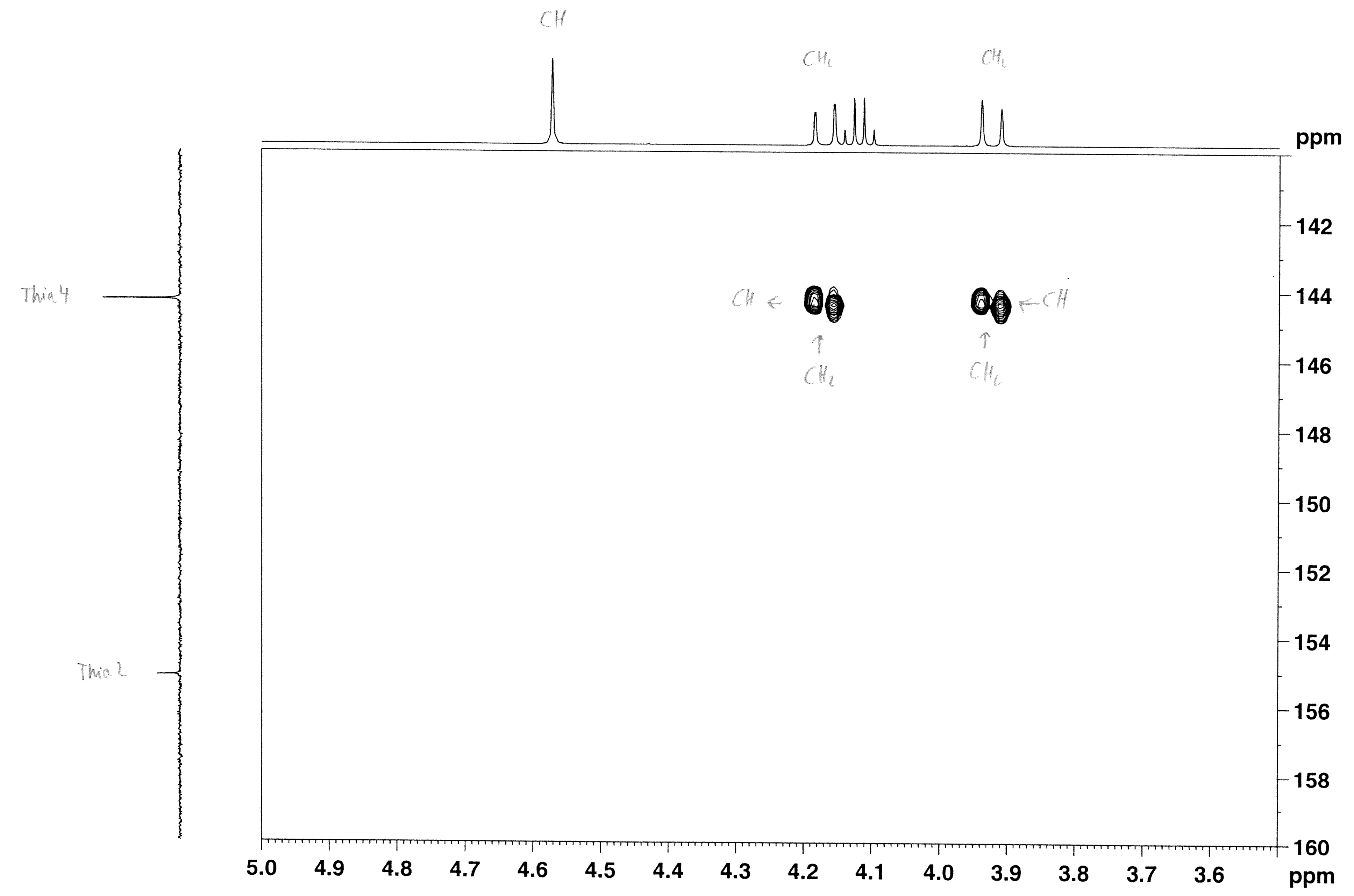

MK026p1 in cdcl3 (HMBC) 4.11.2019

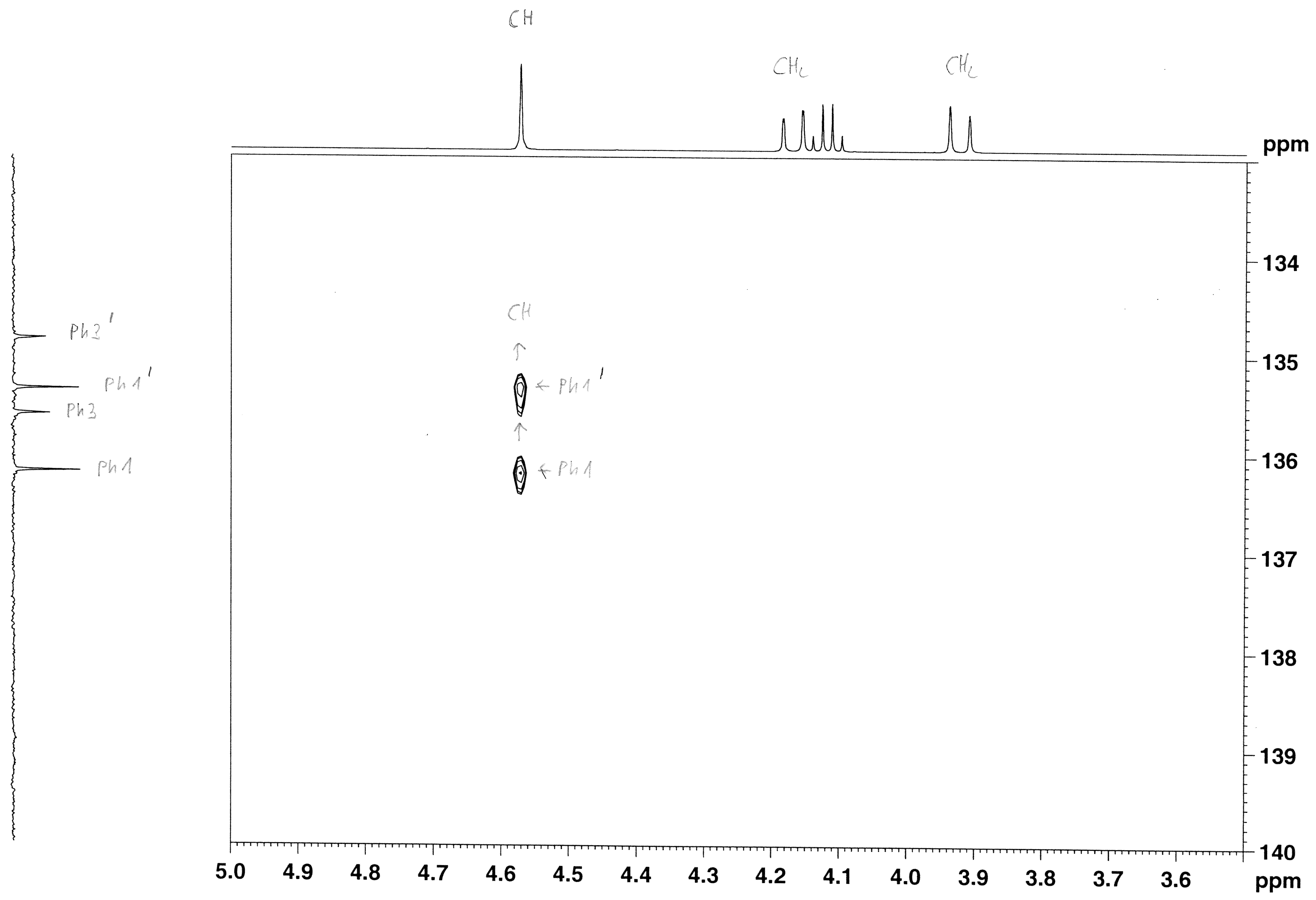

MK026p1 in cdcl3 (HMBC) 4.11.2019

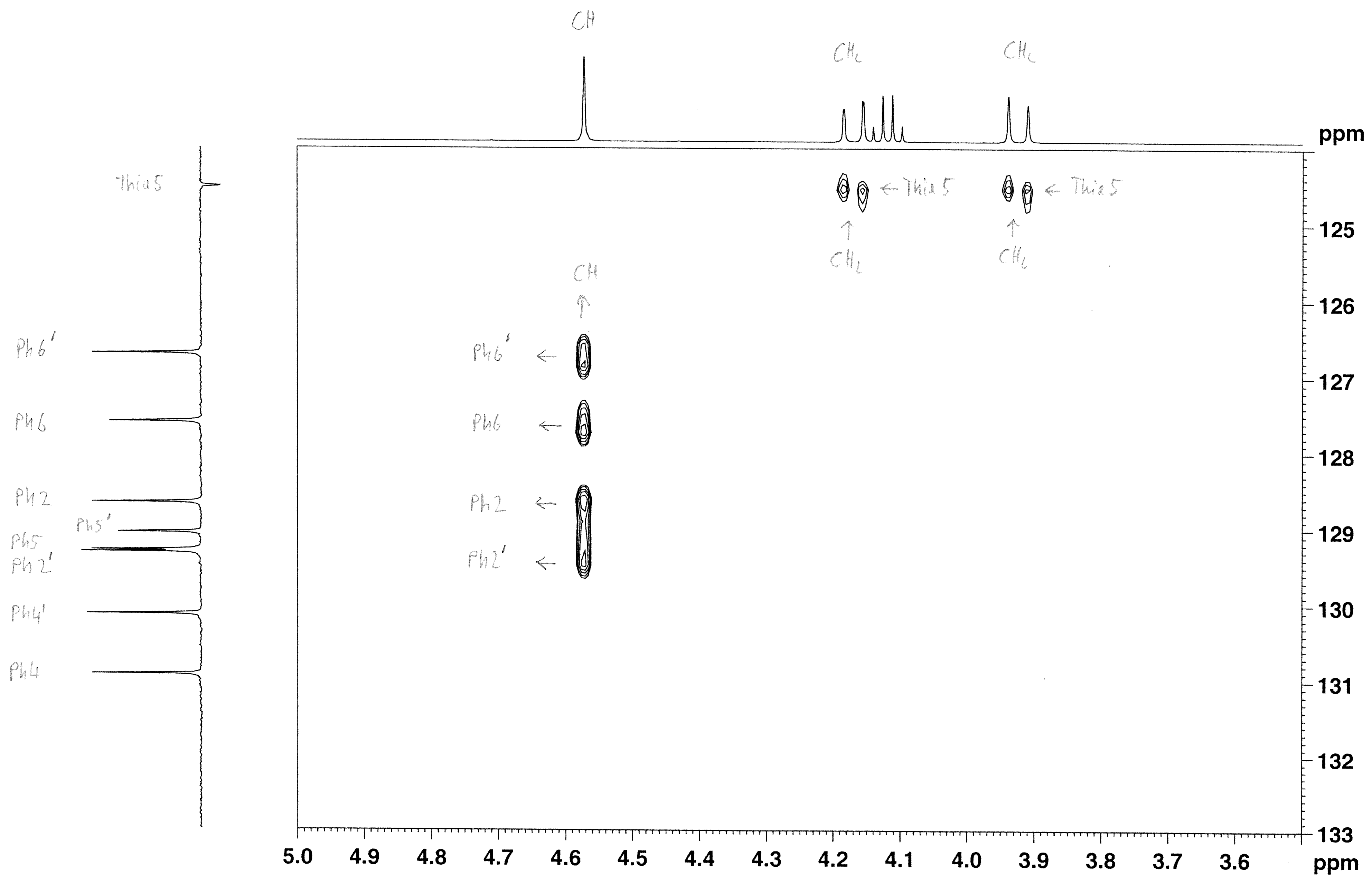

MK026p1 in cdcl3 (HMBC) 4.11.2019

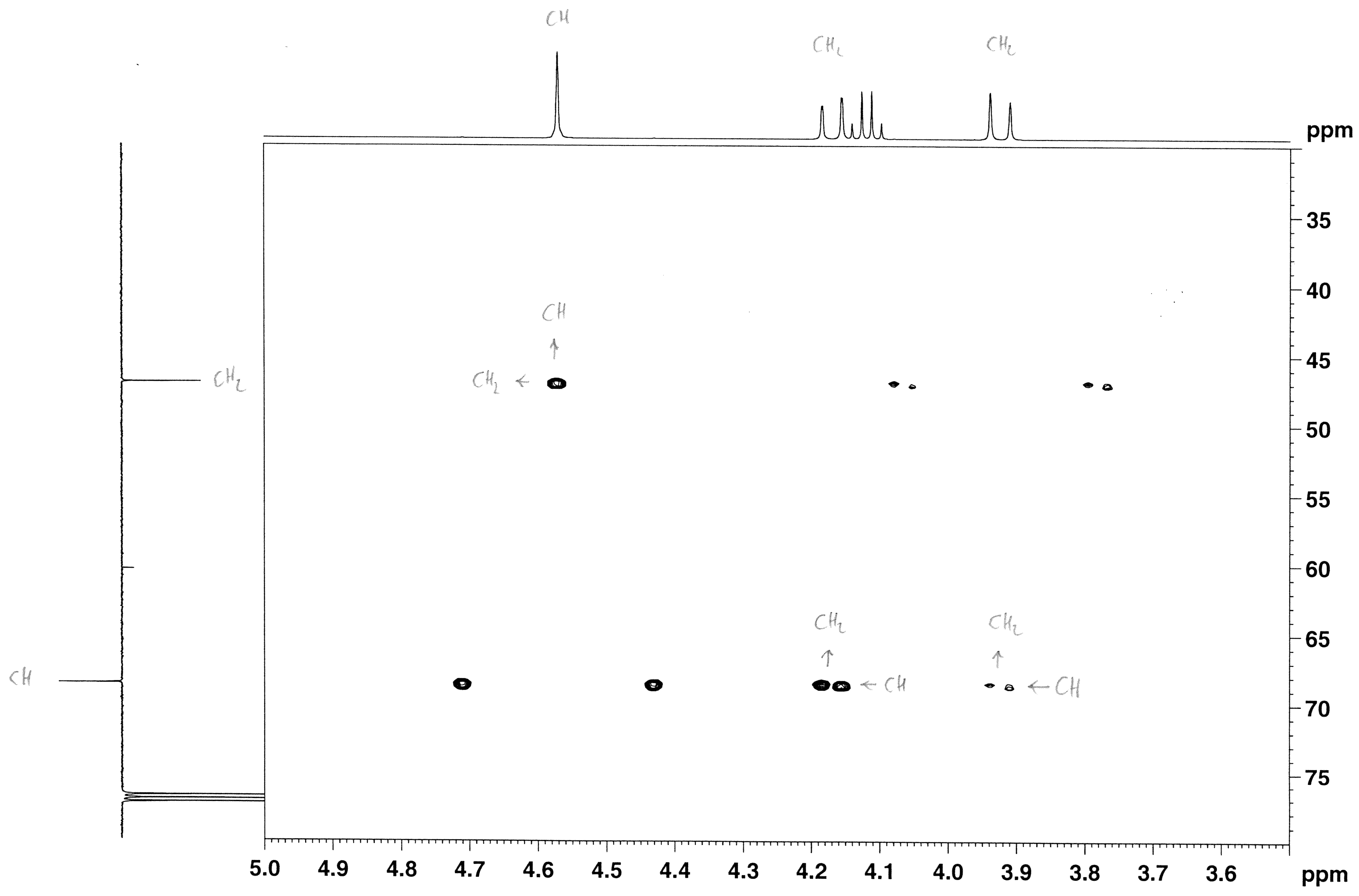

1.2. HRes MS spectra

Generic Display Report

Analysis Info

Analysis Name	D:\Data\Kalaba\68890000001.d	Acquisition Date	29/01/2020 19:03:02
Method	tune_low_MS_Service_01_20.m	Operator	msc
Sample Name	MK-26-peak-I	Instrument	maXis
Comment	Kalaba/Zehl<br>Ergebnis +/- 5ppm<br>ACN/MeOH + 1%H2O		

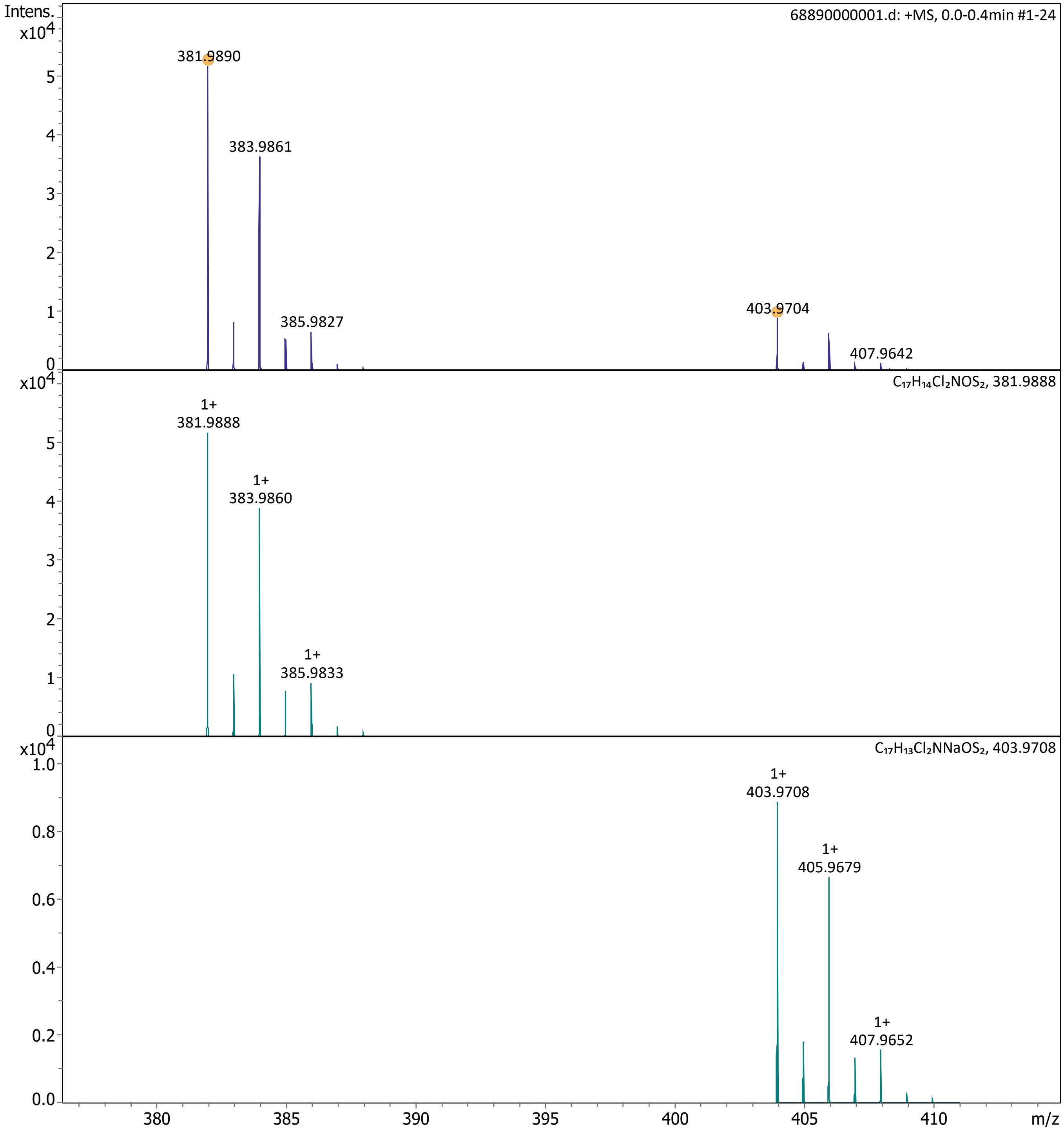

Mass Spectrum SmartFormula Report

Analysis Info

Acquisition Date 29/01/2020 19:03:02

Analysis Name D:\Data\Kalaba\68890000001.d

Method tune_low_MS_Service_01_20.m

Operator msc

Sample Name MK-26-peak-I

Instrument maXis 255552.00016

Comment Kalaba/Zehl
Ergebnis +/- 5ppm
ACN/MeOH + 1%H2O

Acquisition Parameter

Source Type	ESI	Ion Polarity	Positive	Set Nebulizer	0.4 Bar
Focus	Not active	Set Capillary	4200 V	Set Dry Heater	150 Â°C
Scan Begin	50 m/z	Set End Plate Offset	-500 V	Set Dry Gas	4.0 l/min
Scan End	2400 m/z	Set Charging Voltage	0 V	Set Divert Valve	Source
		Set Corona	0 nA	Set APCI Heater	0 Â°C

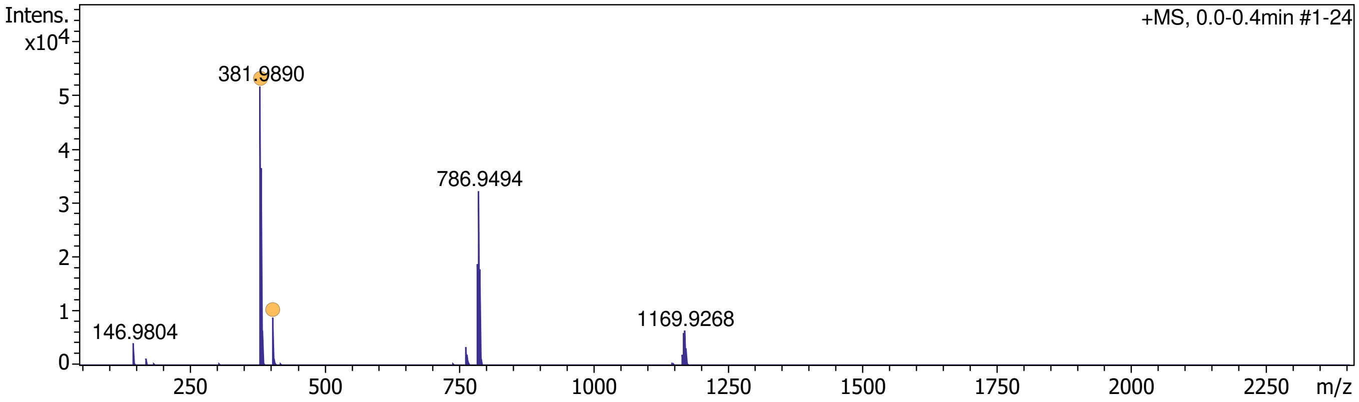

Meas. m/z	#	Ion Formula	m/z	err [ppm]	mSigma	# mSigma	Score	rdb	eÂ¯ Conf	N-Rule
381.9890	1	C10H6Cl2N11S	381.9900	2.6	6.0	1	81.43	21.0	even	ok
	2	C9H10Cl2N7O4S	381.9887	-0.9	13.4	2	100.00	16.0	even	ok
	3	C16H10Cl2NO6	381.9880	-2.7	18.3	3	63.62	18.0	even	ok
	4	C17H6Cl2N5O2	381.9893	0.8	27.2	4	74.89	23.0	even	ok
	5	C17H14Cl2NOS2	381.9888	-0.4	36.3	5	74.99	21.0	even	ok
	6	C9H18Cl2N3O3S3	381.9882	-2.1	44.9	6	43.36	14.0	even	ok
	7	C15H13ClN3OS3	381.9904	3.6	99.2	7	3.36	20.0	even	ok
	8	C12H7Cl3N9	381.9885	-1.4	127.2	8	1.47	22.0	even	ok
	9	C11H11Cl3N5O4	381.9871	-4.9	128.7	9	0.60	17.0	even	ok
	10	C14H9ClN3O6S	381.9895	1.4	148.5	10	0.29	17.0	even	ok
	11	C15H5ClN7O2S	381.9908	4.9	150.8	11	0.11	22.0	even	ok
	12	C11HClN13S	381.9882	-2.2	154.3	12	0.17	23.0	even	ok
	13	C22H5ClNO4	381.9902	3.1	168.3	13	0.05	24.0	even	ok
	14	C18HClN7O2	381.9875	-4.0	170.9	14	0.03	25.0	even	ok
	15	C11H12Cl4N7	381.9903	3.4	188.0	15	0.02	20.0	even	ok
	16	C10H16Cl4N3O4	381.9889	-0.1	189.3	16	0.03	15.0	even	ok
	17	C16H16NS5	381.9881	-2.4	212.8	17	0.00	20.0	even	ok
	18	C12H16NO5S4	381.9906	4.2	232.0	18	0.00	14.0	even	ok
	19	C9H21Cl5NO4	381.9908	4.7	248.7	19	0.00	13.0	even	ok
	20	C15H12NO5S3	381.9872	-4.7	250.8	20	0.00	17.0	even	ok
	21	C16H8N5OS3	381.9885	-1.2	253.1	21	0.00	22.0	even	ok
	22	C11H12NO10S2	381.9897	1.9	295.7	22	0.00	11.0	even	ok
	23	C15H4N5O6S	381.9877	-3.4	318.7	24	0.00	19.0	even	ok
	24	C10H8NO15	381.9888	-0.4	385.8	26	0.00	8.0	even	ok
	25	C11H4N5O11	381.9902	3.1	388.2	28	0.00	13.0	even	ok
403.9704	1	C10H5Cl2N11NaS	403.9719	3.9	13.7	1	58.43	21.0	even	ok
	2	C16H9Cl2NNaO6	403.9699	-1.1	23.2	2	89.80	18.0	even	ok
	3	C17H13Cl2NNaOS2	403.9708	1.0	26.5	3	100.00	21.0	even	ok
	4	C17H5Cl2N5NaO2	403.9713	2.2	28.1	4	65.10	23.0	even	ok

Mass Spectrum SmartFormula Report

Meas. m/z	#	Ion Formula	m/z	err [ppm]	mSigma	# mSigma	Score	rdb	eÂ¯ Conf	N-Rule
	5	C13HCl2N11Na	403.9686	-4.5	28.9	5	35.16	24.0	even	ok
	6	C15H12ClN3NaOS3	403.9723	4.8	106.8	6	1.85	20.0	even	ok
	7	C12H6Cl3N9Na	403.9704	0.0	119.7	7	3.31	22.0	even	ok
	8	C11H10Cl3N5NaO4	403.9691	-3.3	122.0	8	1.49	17.0	even	ok
	9	C18H8ClN3NaOS2	403.9690	-3.5	125.5	9	0.99	23.0	even	ok
	10	C11H18Cl3NNaO3S2	403.9686	-4.4	147.0	10	0.24	15.0	even	ok
	11	C14H8ClN3NaO6S	403.9715	2.7	157.6	11	0.14	17.0	even	ok
	12	C10H4ClN9NaO4S	403.9688	-4.0	162.2	12	0.07	18.0	even	ok
	13	C11ClN13NaS	403.9701	-0.7	163.5	13	0.14	23.0	even	ok
	14	C22H4ClNNaO4	403.9721	4.3	176.0	14	0.02	24.0	even	ok
	15	C18ClN7NaO2	403.9694	-2.4	179.1	15	0.03	25.0	even	ok
	16	C11H11Cl4N7Na	403.9722	4.6	183.3	16	0.02	20.0	even	ok
	17	C10H15Cl4N3NaO4	403.9709	1.3	185.1	17	0.04	15.0	even	ok
	18	C16H15NNaS5	403.9700	-0.9	221.4	18	0.00	20.0	even	ok
	19	C15H11NNaO5S3	403.9692	-3.0	259.8	19	0.00	17.0	even	ok
	20	C16H7N5NaOS3	403.9705	0.3	261.8	20	0.00	22.0	even	ok
	21	C11H11NNaO10S2	403.9717	3.2	305.7	21	0.00	11.0	even	ok
	22	C15H3N5NaO6S	403.9696	-1.9	328.2	22	0.00	19.0	even	ok
	23	C10H7NNaO15	403.9708	1.0	395.8	23	0.00	8.0	even	ok
	24	C11H3N5NaO11	403.9721	4.3	398.0	24	0.00	13.0	even	ok

1.3. General purity determined by HPLC on a C18 column

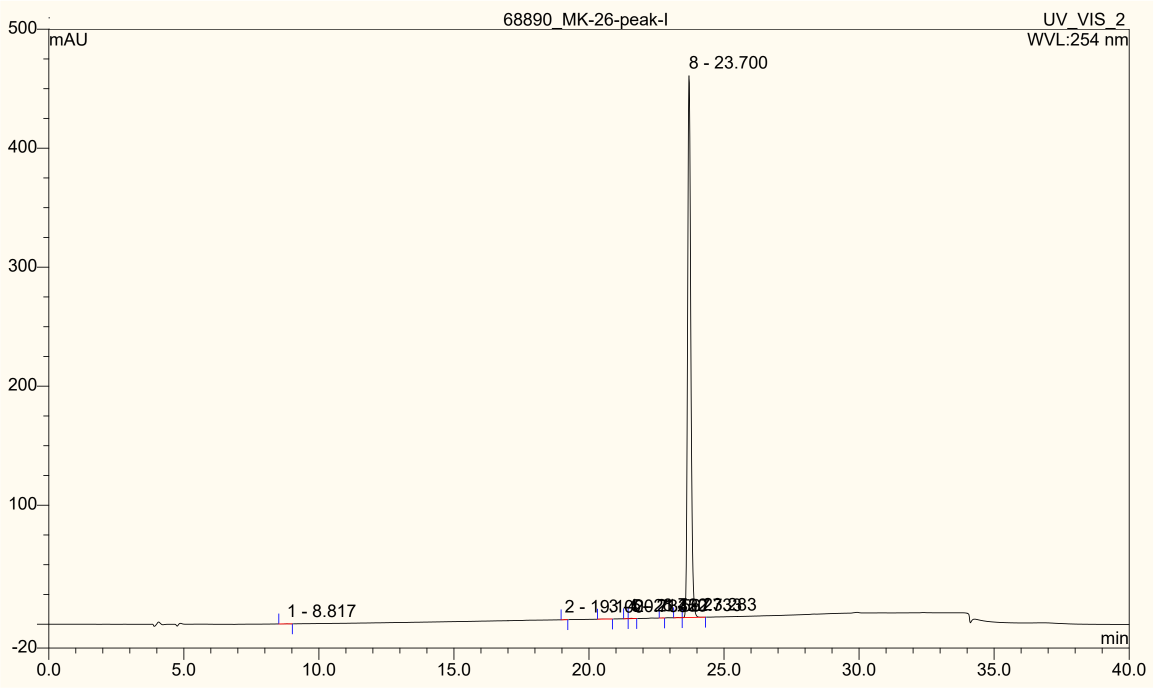

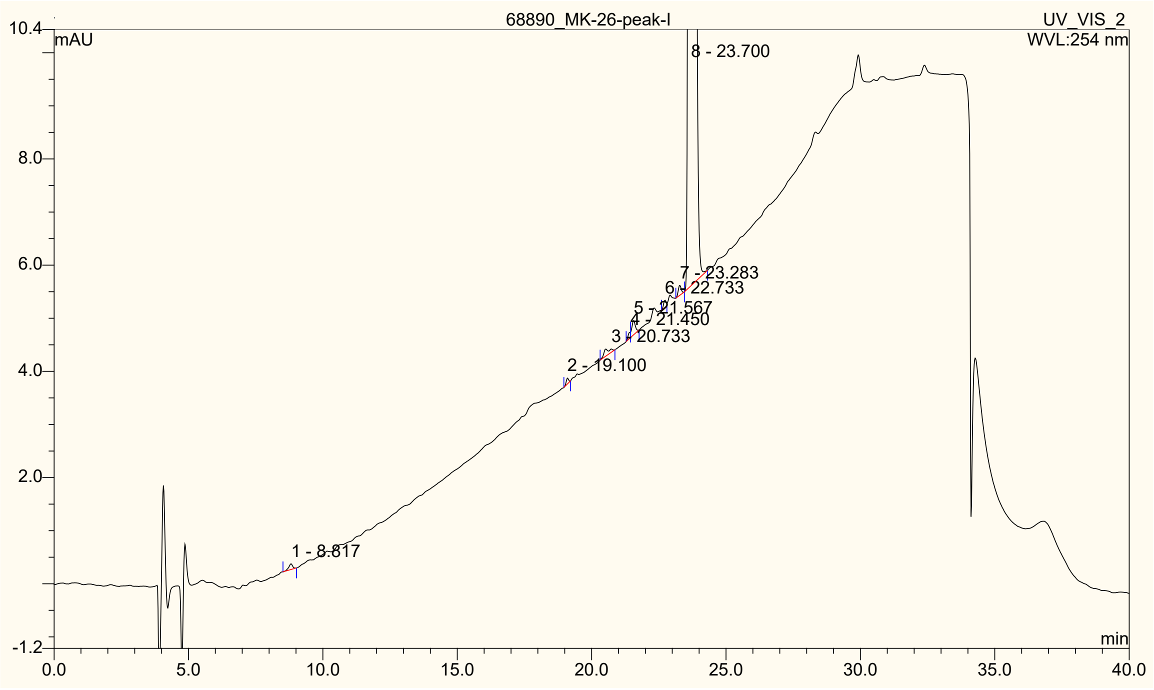

Retention Time: **23.70 min**

Relative Peak Area: **99.76 %**

1.4. Chiral purity

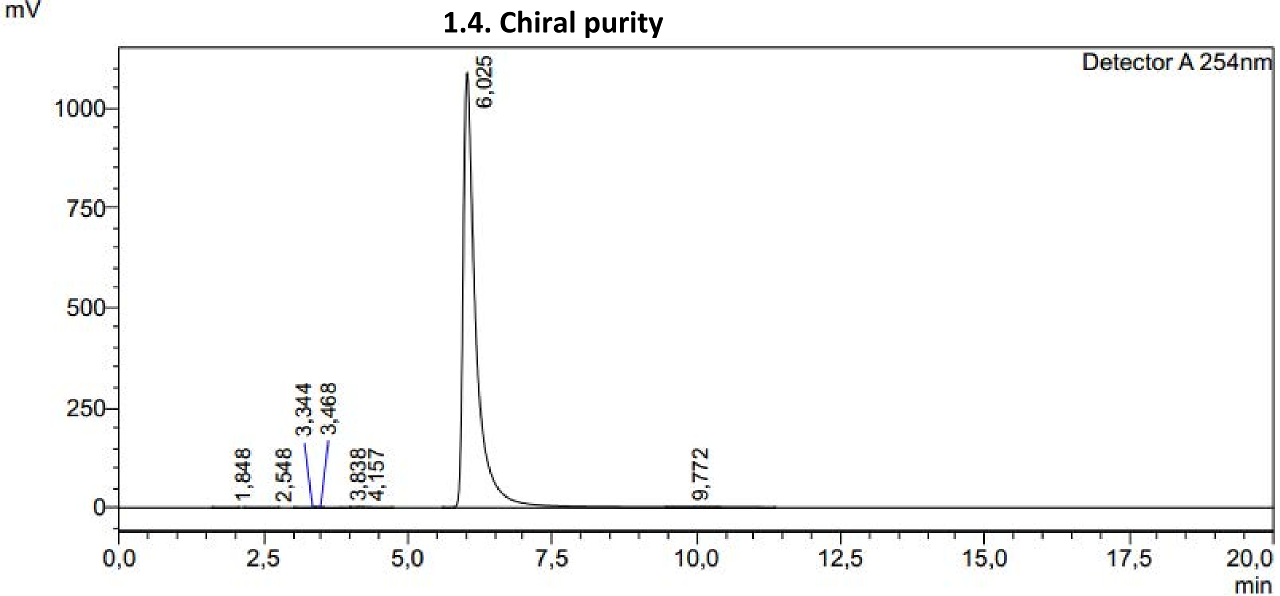

<Peak Table>

Detector A 254nm

Peak#	Ret. Time	Area	Area%
1	1,848	2888	0,017
2	2,548	2535	0,015
3	3,344	6720	0,040
4	3,468	11071	0,066
5	3,838	15963	0,095
6	4,157	32213	0,191
7	6,025	16745789	99,526
8	9,772	8327	0,049
Total		16825507	100,000

2. Chirally resolved *(R)*-MK-26 after racemic synthesis

2.1. ^{1}H and ^{13}C NMR spectra

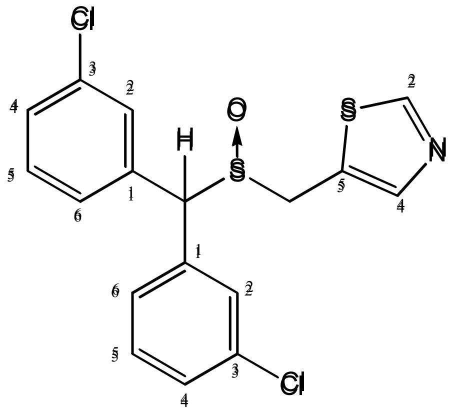

**MK026p2** in $CDCl_3$		^{1}H	^{13}C
Thia 2	CH	8,88	155,16
Thia 4	CH	7,71	144,29
Thia 5	C	--	124,51
Ph 1	C	--	136,20
Ph 1'	C	--	135,36
Ph 2	CH	7,36	128,67
Ph 2'	CH	7,34	129,32
Ph 3	C	--	135,62
Ph 3'	C	--	134,85
Ph 4	CH	7,41	130,93
Ph 4'	CH	7,33	130,14
Ph 5	CH	7,40	129,29
Ph 5'	CH	7,34	129,06
Ph 6	CH	7,27	127,60
Ph 6'	CH	7,28	126,70
CH	CH	4,57	68,55
CH_2	CH_2	4,17/3,92	46,96

MK026p2 in cdcl3 (Proton) 4.11.2019

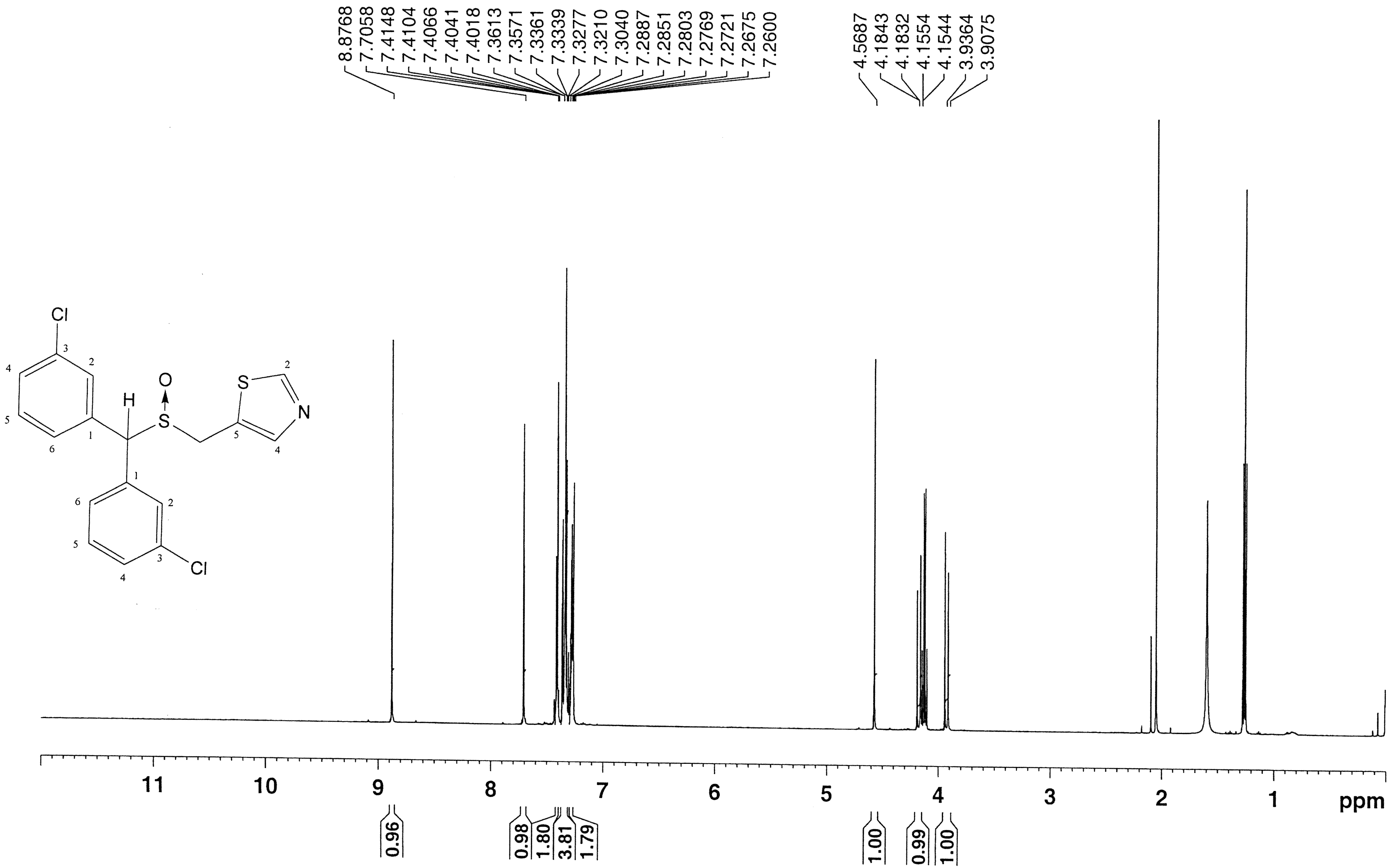

MK026p2 in cdcl3 (Proton) 4.11.2019

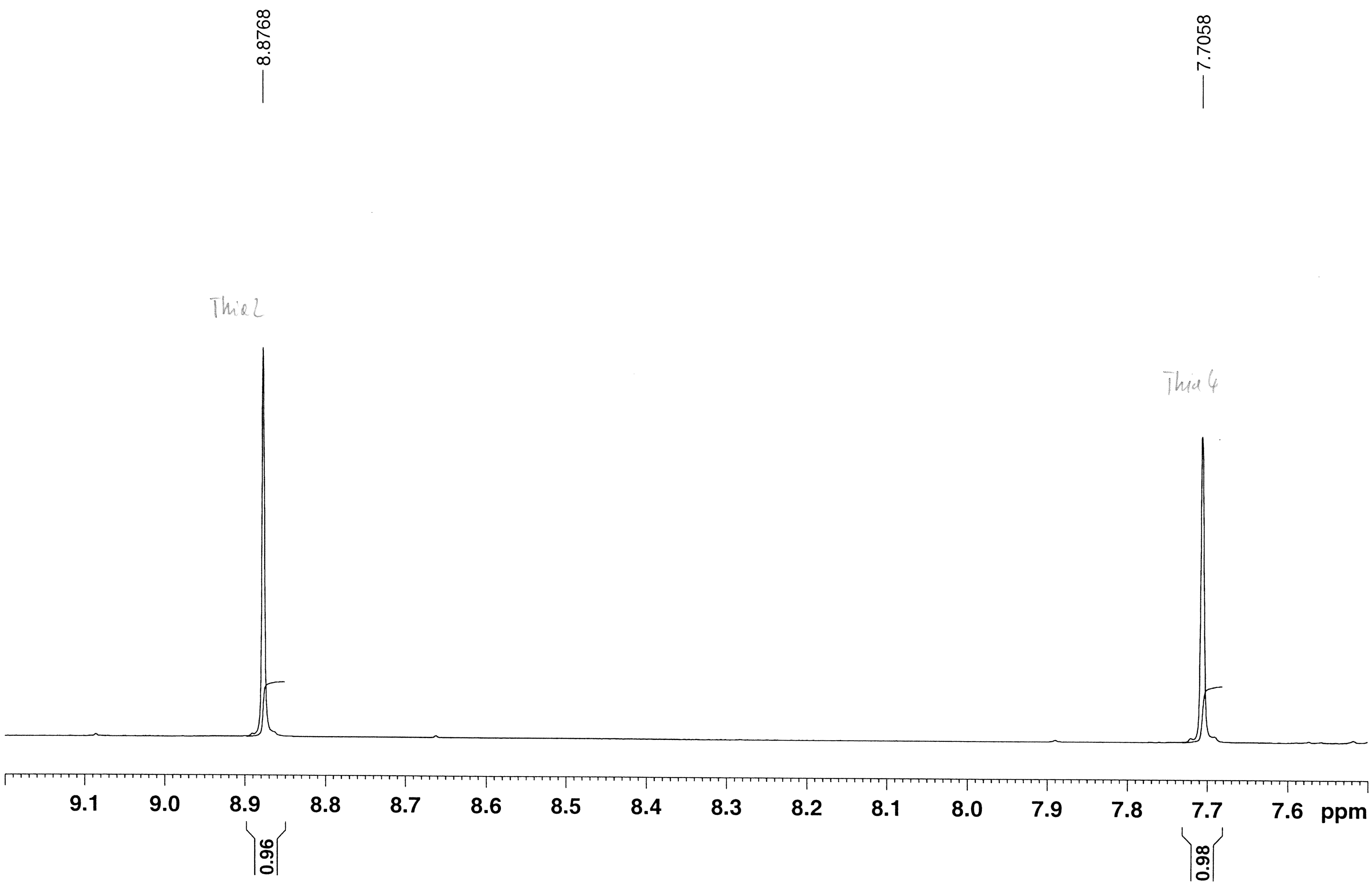

MK026p2 in cdcl3 (Proton) 4.11.2019

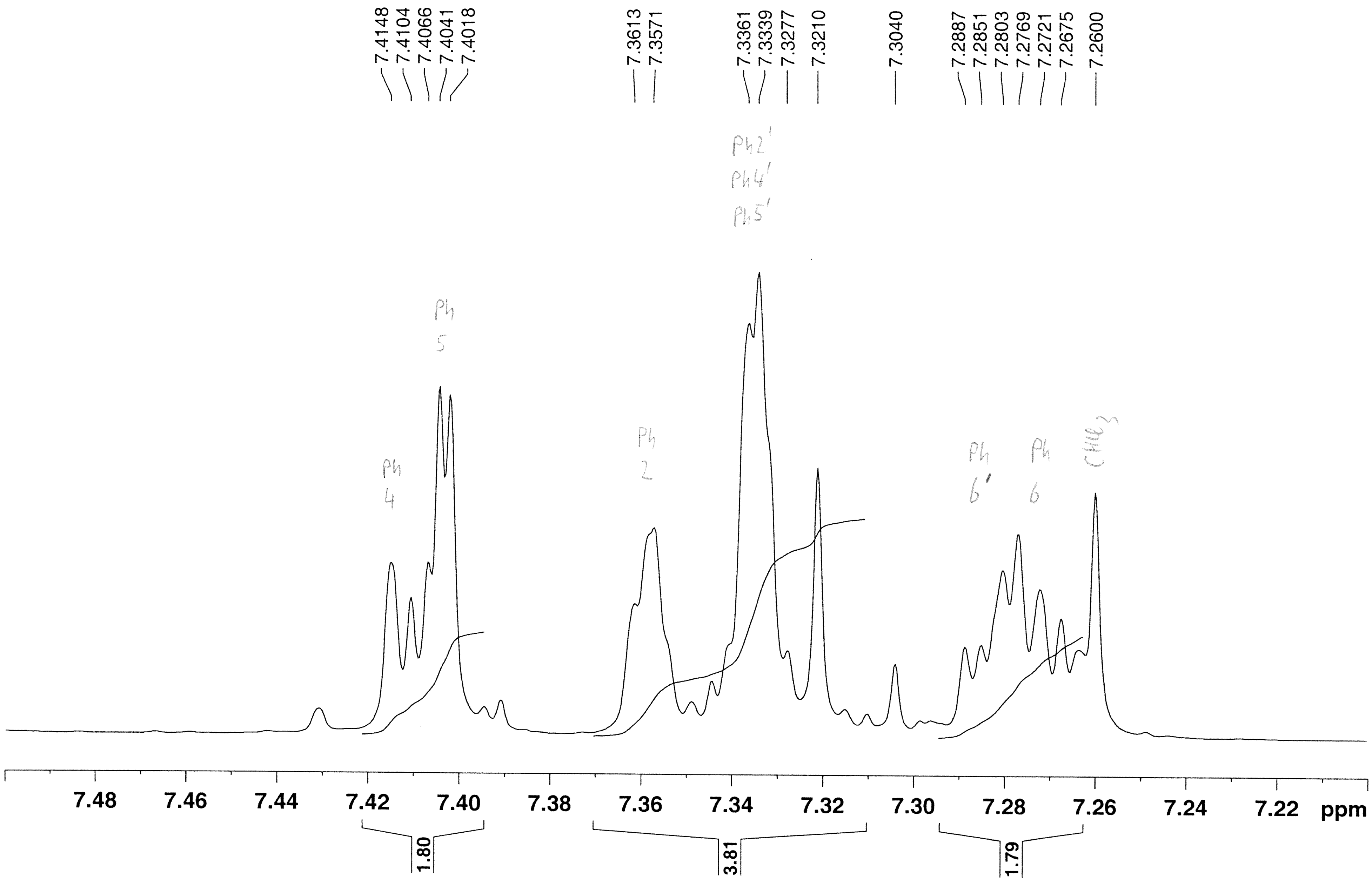

MK026p2 in cdcl3 (Proton) 4.11.2019

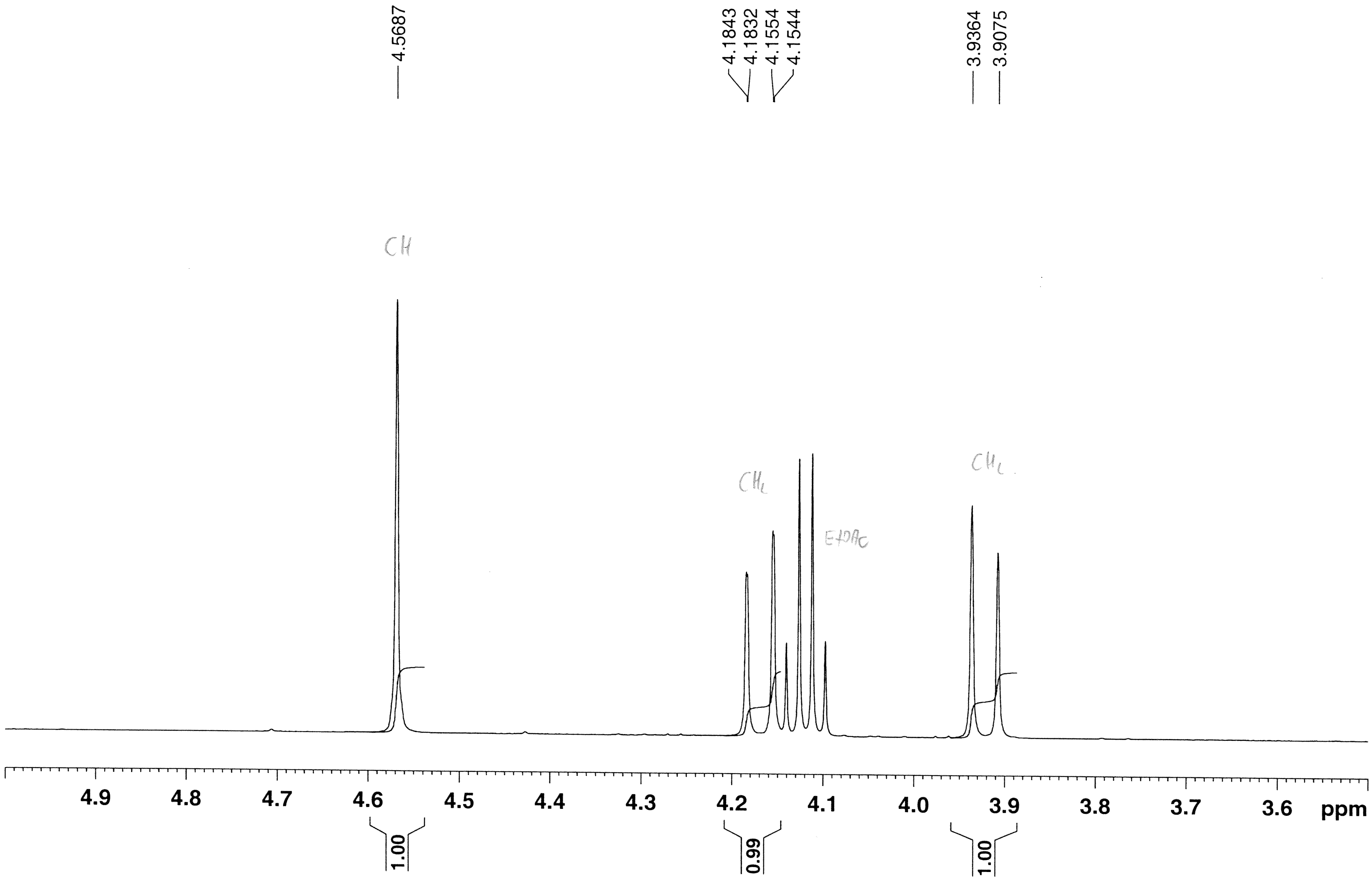

MK026p2 in cdcl3 (APT) 4.11.2019

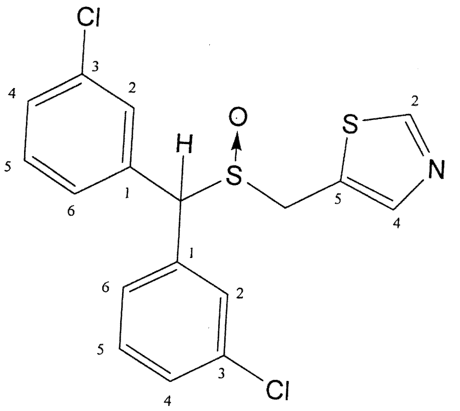

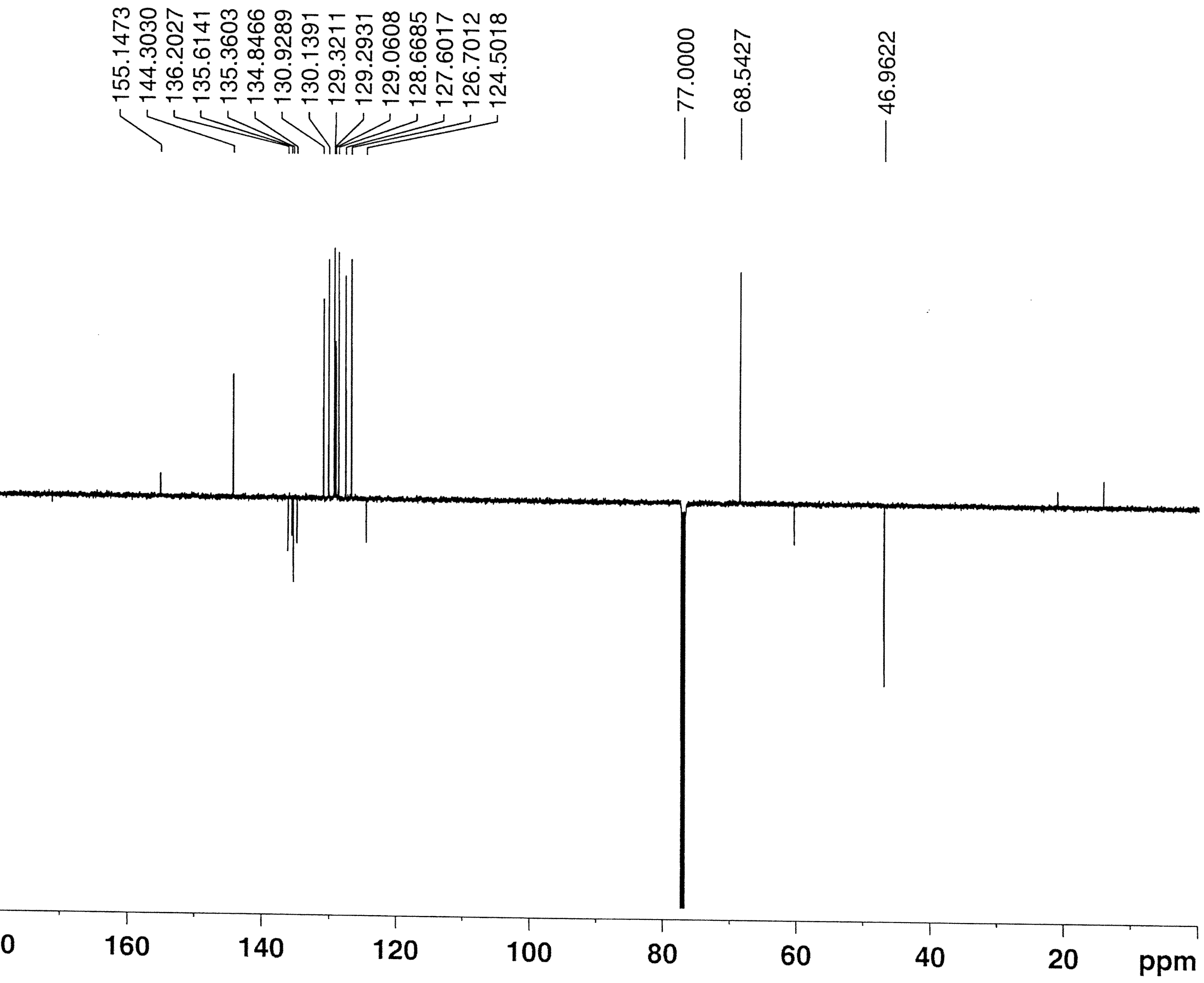

MK026p2 in cdcl3 (APT) 4.11.2019

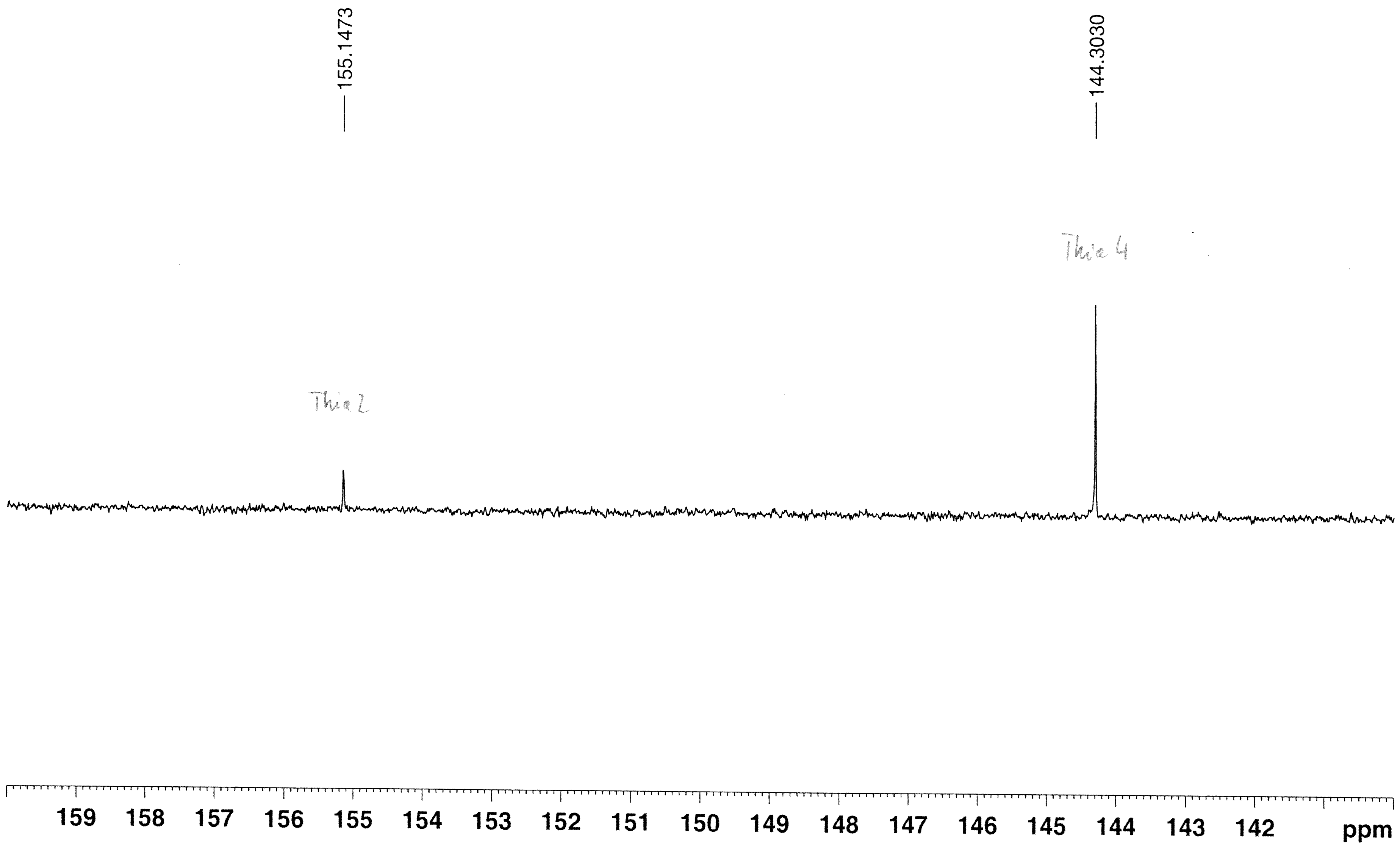

MK026p2 in cdcl3 (APT) 4.11.2019

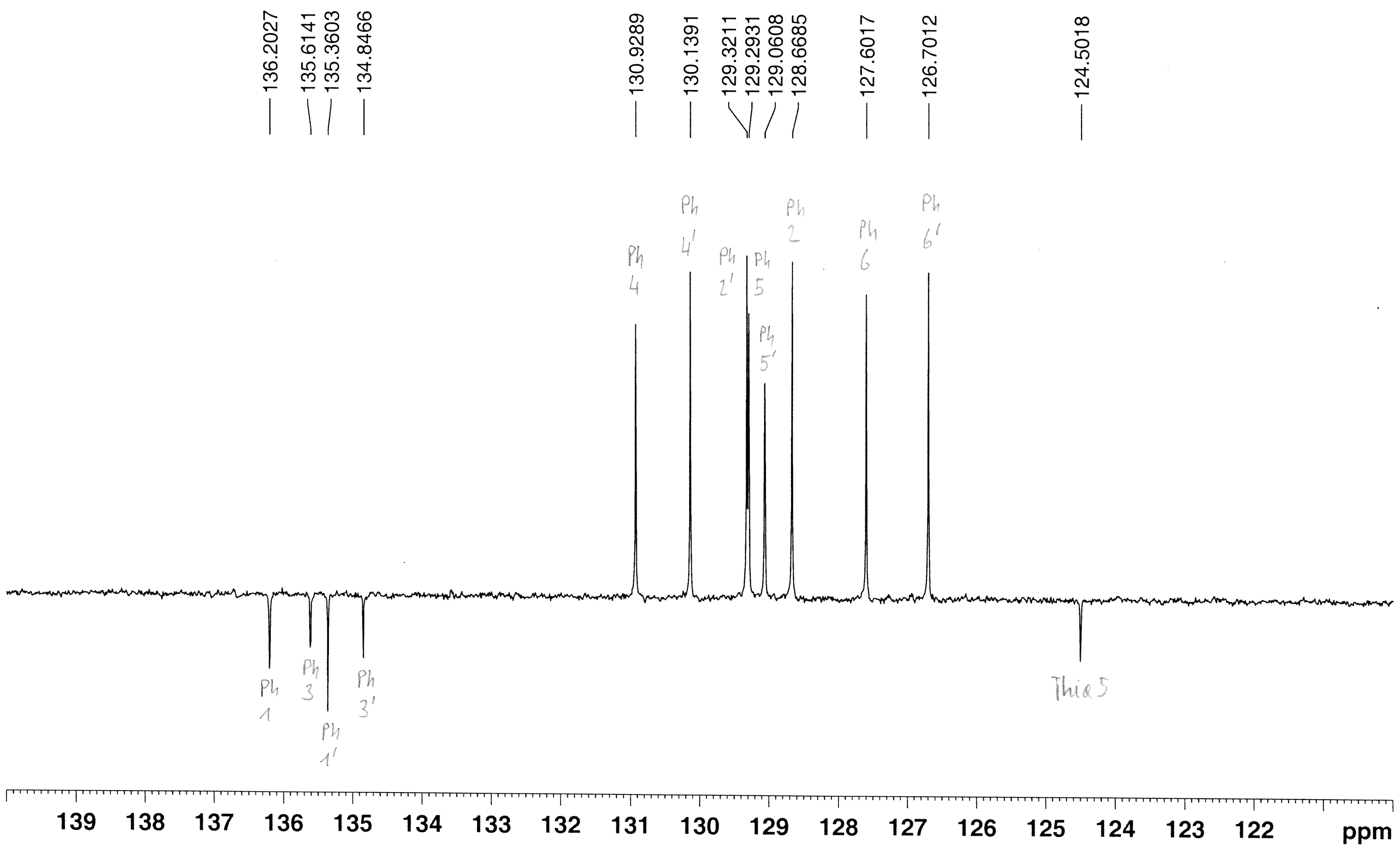

MK026p2 in cdcl3 (APT) 4.11.2019

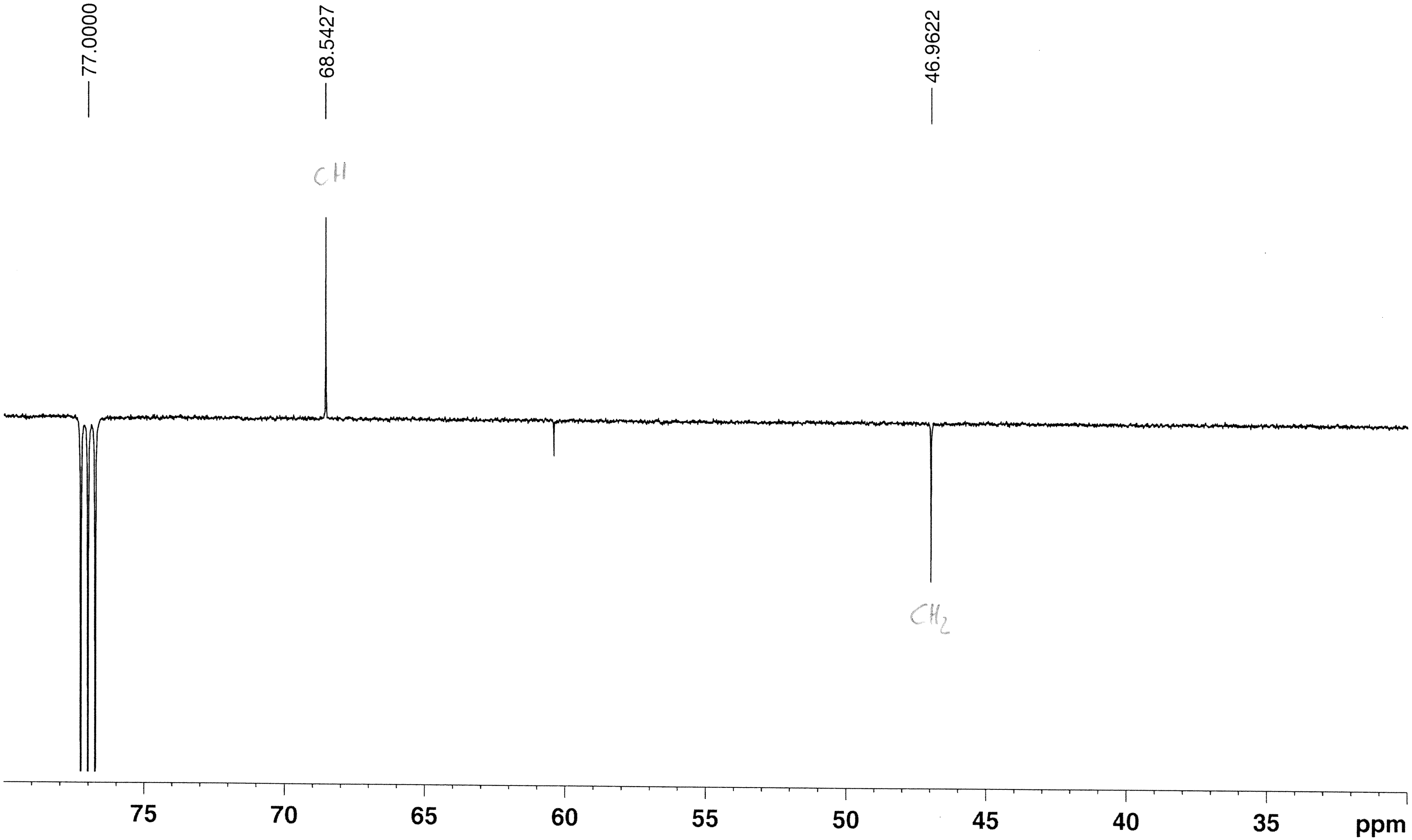

2.2. HRes MS spectra

Generic Display Report

Analysis Info

Analysis Name	D:\Data\Kalaba\68891000001.d	Acquisition Date	29/01/2020 19:05:14
Method	tune_low_MS_Service_01_20.m	Operator	msc
Sample Name	MK-26-peak-II	Instrument	maXis
Comment	Kalaba/Zehl<br>Ergebnis +/- 5ppm<br>ACN/MeOH + 1%H2O		

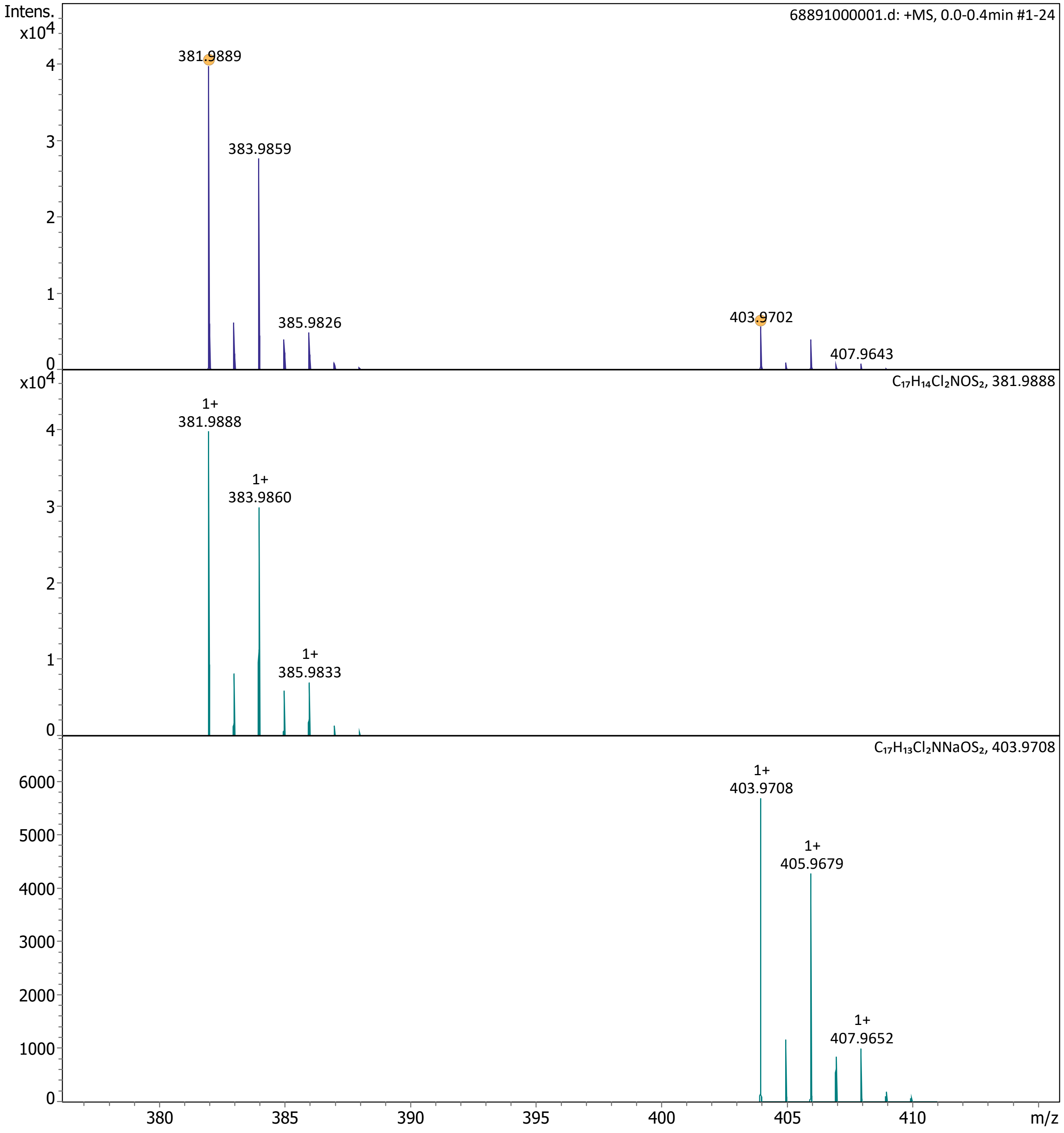

Mass Spectrum SmartFormula Report

Analysis Info

Analysis Name	D:\Data\Kalaba\68891000001.d
Method	tune_low_MS_Service_01_20.m
Sample Name	MK-26-peak-II
Comment	Kalaba/Zehl<br>Ergebnis +/- 5ppm<br>ACN/MeOH + 1%H2O

Acquisition Date	29/01/2020 19:05:14	
Operator	msc	
Instrument	maXis	255552.00016

Acquisition Parameter

Source Type	ESI	Ion Polarity	Positive	Set Nebulizer	0.4 Bar
Focus	Not active	Set Capillary	4200 V	Set Dry Heater	150 Â°C
Scan Begin	50 m/z	Set End Plate Offset	-500 V	Set Dry Gas	4.0 l/min
Scan End	2400 m/z	Set Charging Voltage	0 V	Set Divert Valve	Source
		Set Corona	0 nA	Set APCI Heater	0 Â°C

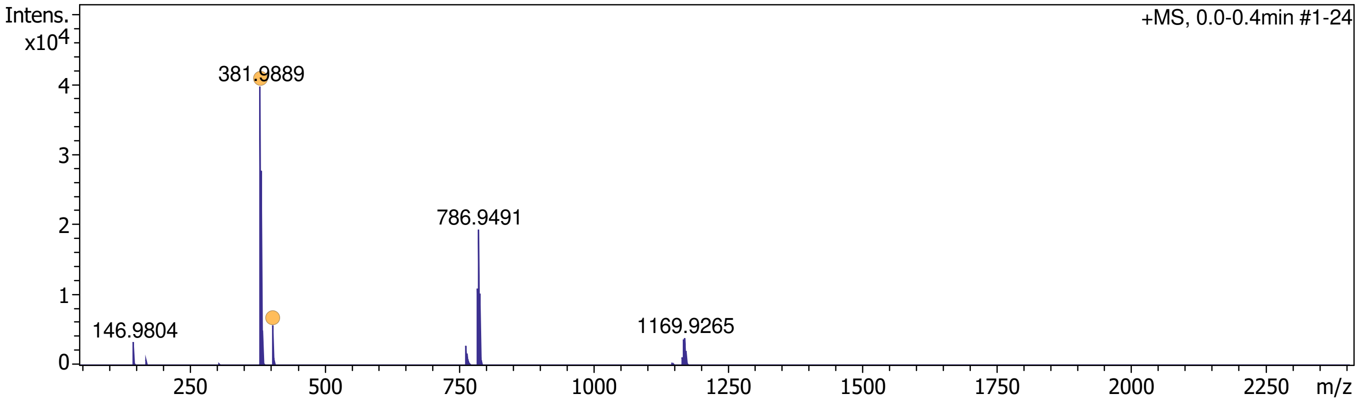

Meas. m/z	#	Ion Formula	m/z	err [ppm]	mSigma	# mSigma	Score	rdb	eÂ¯ Conf	N-Rule
381.9889	1	C10H6Cl2N11S	381.9900	2.8	6.5	1	75.04	21.0	even	ok
	2	C9H10Cl2N7O4S	381.9887	-0.7	13.1	2	100.00	16.0	even	ok
	3	C16H10Cl2NO6	381.9880	-2.5	16.3	3	66.54	18.0	even	ok
	4	C17H6Cl2N5O2	381.9893	1.0	26.2	4	71.80	23.0	even	ok
	5	C17H14Cl2NOS2	381.9888	-0.2	39.3	5	68.73	21.0	even	ok
	6	C9H18Cl2N3O3S3	381.9882	-1.9	47.8	6	39.86	14.0	even	ok
	7	C15H13ClN3OS3	381.9904	3.8	95.8	7	3.64	20.0	even	ok
	8	C18H9ClN3OS2	381.9870	-5.0	115.0	8	1.02	23.0	even	ok
	9	C12H7Cl3N9	381.9885	-1.2	130.5	9	1.21	22.0	even	ok
	10	C11H11Cl3N5O4	381.9871	-4.7	131.9	10	0.50	17.0	even	ok
	11	C14H9ClN3O6S	381.9895	1.5	144.5	11	0.35	17.0	even	ok
	12	C15H5ClN7O2S	381.9908	5.0	146.9	12	0.13	22.0	even	ok
	13	C11HClN13S	381.9882	-2.0	150.3	13	0.22	23.0	even	ok
	14	C22H5ClNO4	381.9902	3.2	164.6	14	0.06	24.0	even	ok
	15	C18HClN7O2	381.9875	-3.8	167.1	15	0.05	25.0	even	ok
	16	C11H12Cl4N7	381.9903	3.6	190.4	16	0.01	20.0	even	ok
	17	C10H16Cl4N3O4	381.9889	0.1	191.6	17	0.02	15.0	even	ok
	18	C16H16NS5	381.9881	-2.2	209.0	18	0.00	20.0	even	ok
	19	C12H16NO5S4	381.9906	4.4	228.0	19	0.00	14.0	even	ok
	20	C15H12NO5S3	381.9872	-4.5	246.9	20	0.00	17.0	even	ok
	21	C16H8N5OS3	381.9885	-1.0	249.2	21	0.00	22.0	even	ok
	22	C9H21Cl5NO4	381.9908	4.8	250.5	22	0.00	13.0	even	ok
	23	C11H12NO10S2	381.9897	2.1	291.4	23	0.00	11.0	even	ok
	24	C15H4N5O6S	381.9877	-3.2	314.5	25	0.00	19.0	even	ok
	25	C10H8NO15	381.9888	-0.2	380.9	27	0.00	8.0	even	ok
	26	C11H4N5O11	381.9902	3.3	383.4	29	0.00	13.0	even	ok
403.9702	1	C10H5Cl2N11NaS	403.9719	4.4	13.3	1	48.29	21.0	even	ok
	2	C16H9Cl2NNaO6	403.9699	-0.6	21.1	2	100.00	18.0	even	ok
	3	C17H13Cl2NNaOS2	403.9708	1.6	25.3	3	88.45	21.0	even	ok

Mass Spectrum SmartFormula Report

Meas. m/z	#	Ion Formula	m/z	err [ppm]	mSigma	# mSigma	Score	rdb	eÂ¯ Conf	N-Rule
	4	C17H5Cl2N5NaO2	403.9713	2.7	25.4	4	58.58	23.0	even	ok
	5	C13HCl2N11Na	403.9686	-3.9	27.0	5	41.36	24.0	even	ok
	6	C10H12ClN5NaO3S3	403.9683	-4.6	107.7	6	1.82	16.0	even	ok
	7	C12H6Cl3N9Na	403.9704	0.6	120.6	7	2.75	22.0	even	ok
	8	C18H8ClN3NaOS2	403.9690	-3.0	123.1	8	1.25	23.0	even	ok
	9	C11H10Cl3N5NaO4	403.9691	-2.7	123.1	9	1.54	17.0	even	ok
	10	C11H18Cl3NNaO3S2	403.9686	-3.9	147.7	10	0.26	15.0	even	ok
	11	C14H8ClN3NaO6S	403.9715	3.2	155.4	11	0.13	17.0	even	ok
	12	C10H4ClN9NaO4S	403.9688	-3.4	160.1	12	0.09	18.0	even	ok
	13	C11ClN13NaS	403.9701	-0.1	161.3	13	0.17	23.0	even	ok
	14	C22H4ClNNaO4	403.9721	4.8	173.4	14	0.02	24.0	even	ok
	15	C18ClN7NaO2	403.9694	-1.8	176.6	15	0.04	25.0	even	ok
	16	C10H15Cl4N3NaO4	403.9709	1.8	185.1	16	0.03	15.0	even	ok
	17	C16H15NNaS5	403.9700	-0.3	218.5	17	0.00	20.0	even	ok
	18	C15H11NNaO5S3	403.9692	-2.5	257.0	18	0.00	17.0	even	ok
	19	C16H7N5NaOS3	403.9705	0.8	259.0	19	0.00	22.0	even	ok
	20	C11H11NNaO10S2	403.9717	3.7	301.8	20	0.00	11.0	even	ok
	21	C14H7NNaO10S	403.9683	-4.6	322.0	21	0.00	14.0	even	ok
	22	C15H3N5NaO6S	403.9696	-1.3	324.1	22	0.00	19.0	even	ok
	23	C10H7NNaO15	403.9708	1.6	390.6	23	0.00	8.0	even	ok
	24	C11H3N5NaO11	403.9721	4.9	392.8	24	0.00	13.0	even	ok

2.3. General purity determined by HPLC on a C18 column

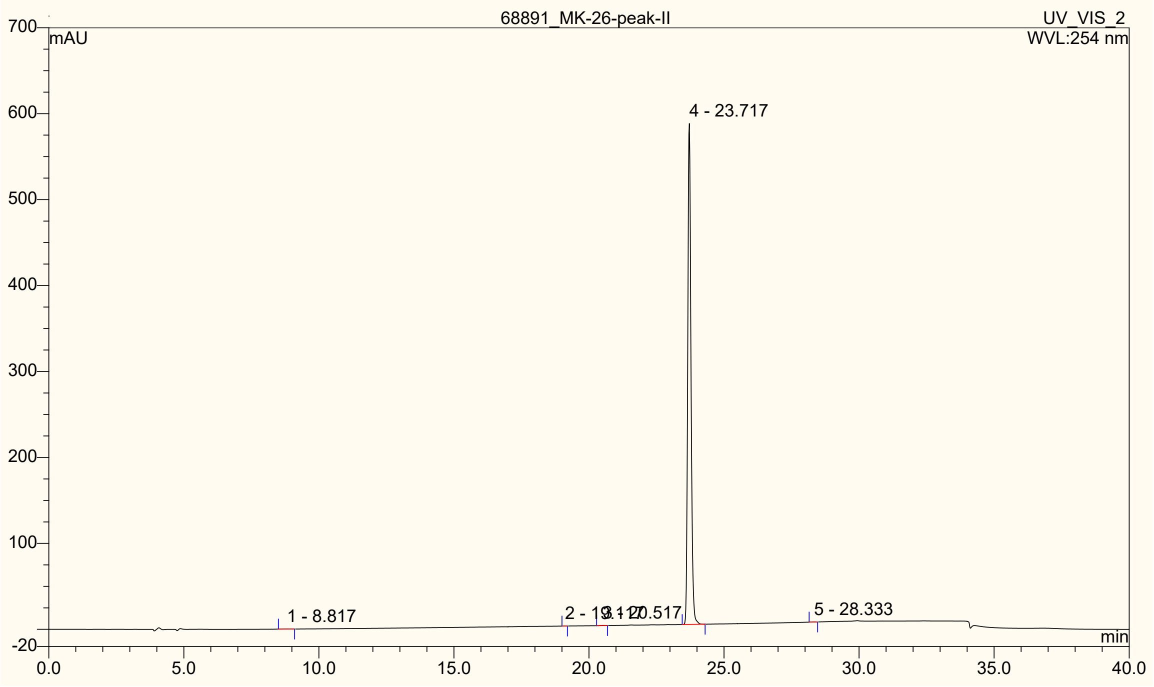

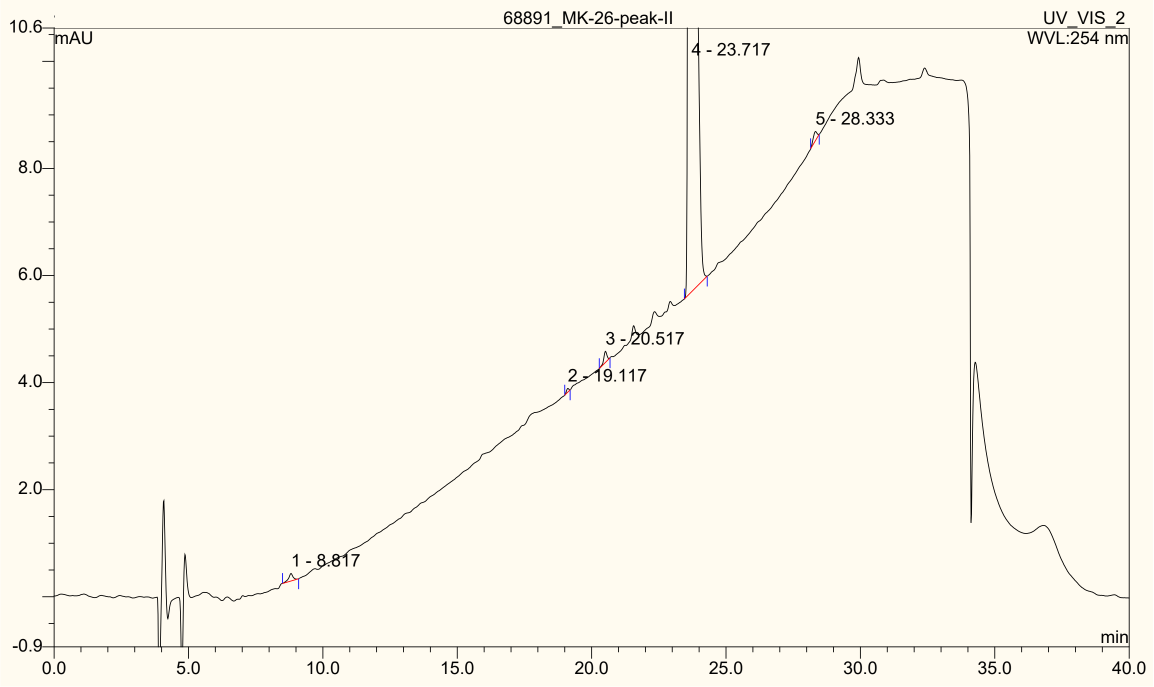

Retention Time: **23.72 min**

Relative Peak Area: **99.89 %**

2.4. Chiral purity

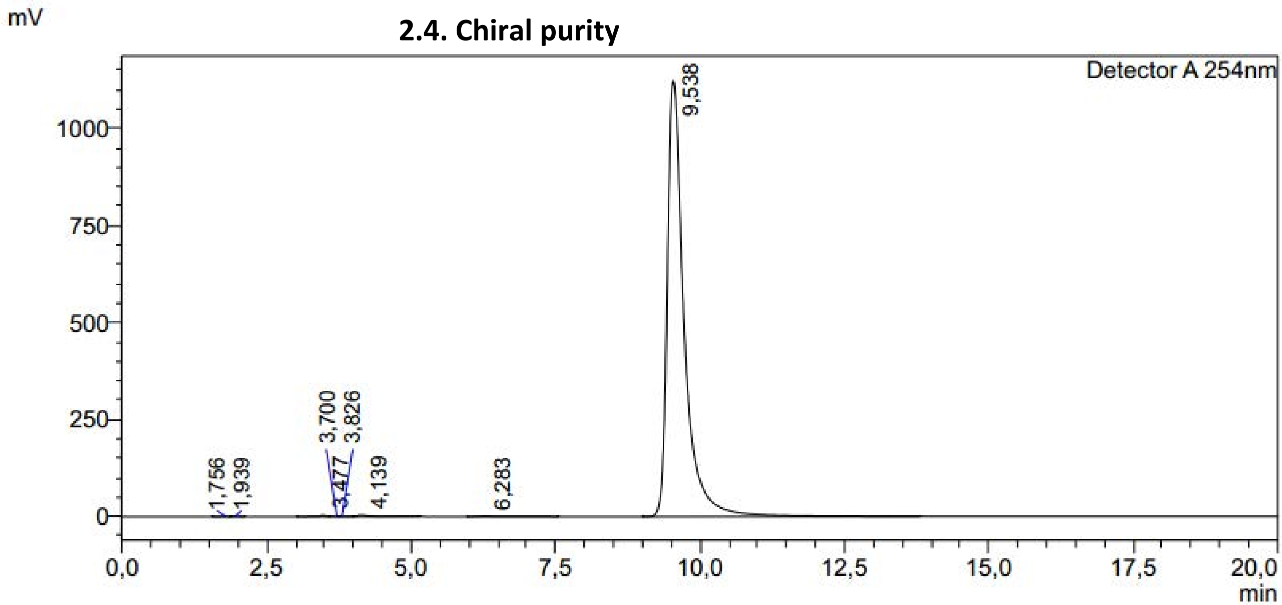

<Peak Table>

Detector A 254nm

Peak#	Ret. Time	Area	Area%
1	1,756	1405	0,006
2	1,939	1477	0,006
3	3,477	24351	0,106
4	3,700	4444	0,019
5	3,826	8293	0,036
6	4,139	48683	0,211
7	6,283	26292	0,114
8	9,538	22934377	99,501
Total		23049321	100,000

3. Enantioselectively synthesised *(S)*-MK-26

3.1. ^{1}H and ^{13}C NMR spectra

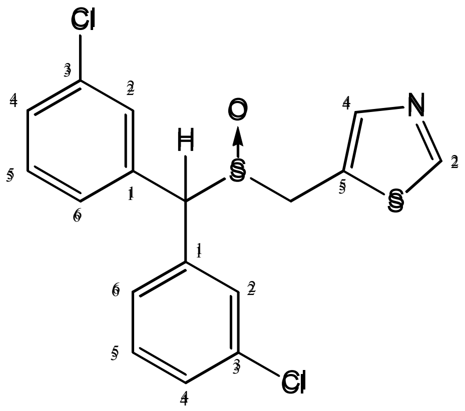

**VD-US26** in $CDCl_3$		^{1}H	^{13}C
Thia 2	CH	8,83	155,12
Thia 4	CH	7,66	144,28
Thia 5	C	--	124,49
Ph 1	C	--	136,19
Ph 1	C	--	135,35
Ph 2	CH	7,30	129,30
Ph 2	CH	7,32	128,64
Ph 3	C	--	135,58
Ph 3	C	--	134,81
Ph 4	CH	7,36	129,26
Ph 4	CH	7,29	129,03
Ph 5	CH	7,36	130,90
Ph 5	CH	7,29	130,12
Ph 6	CH	7,23	127,58
Ph 6	CH	7,24	126,68
CH	CH	4,53	68,51
CH_2	CH_2	4,13/3,88	46,95

VD-US26 in cdcl3 (Proton) 26.2.2021

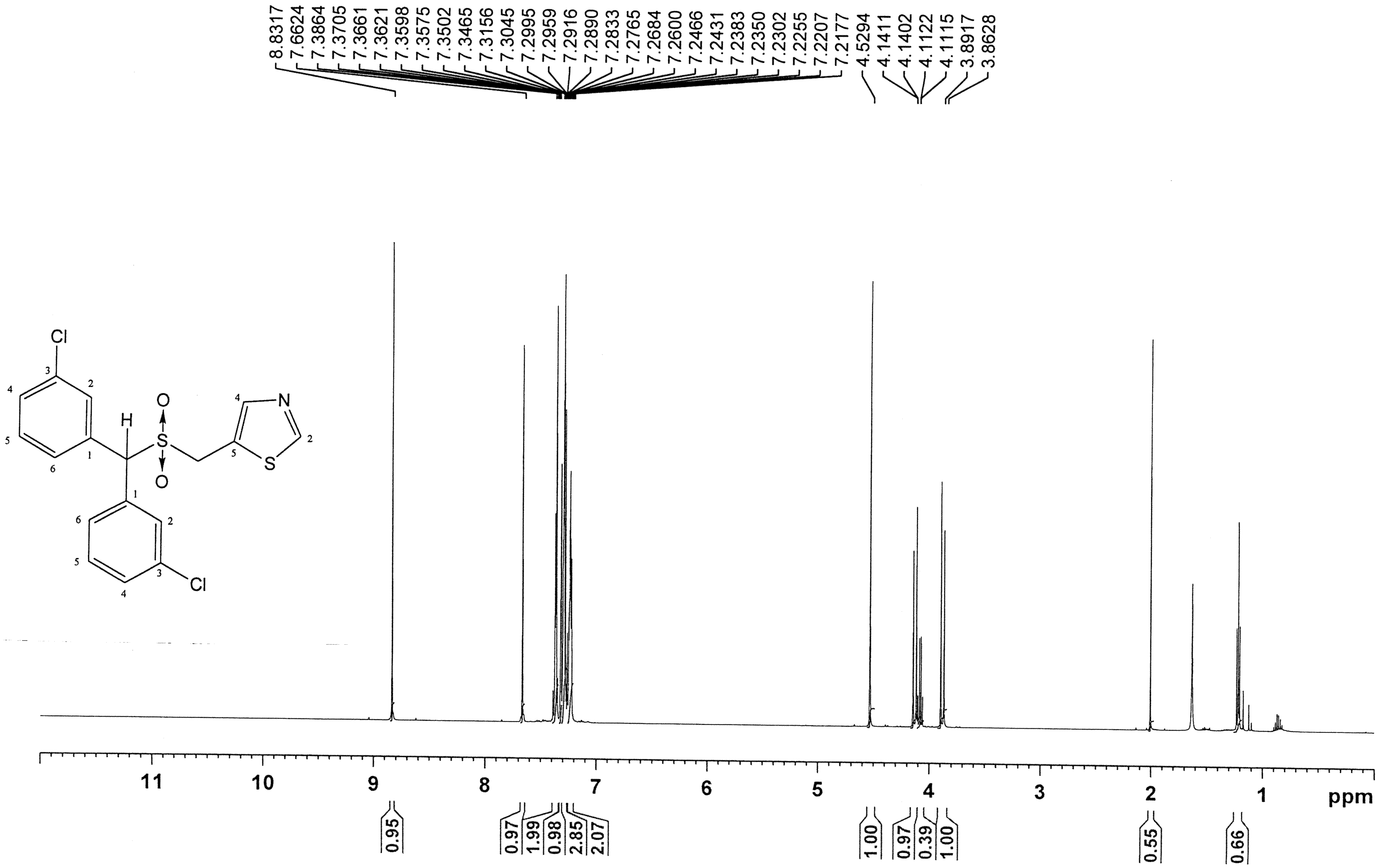

VD-US26 in cdcl3 (Proton) 26.2.2021

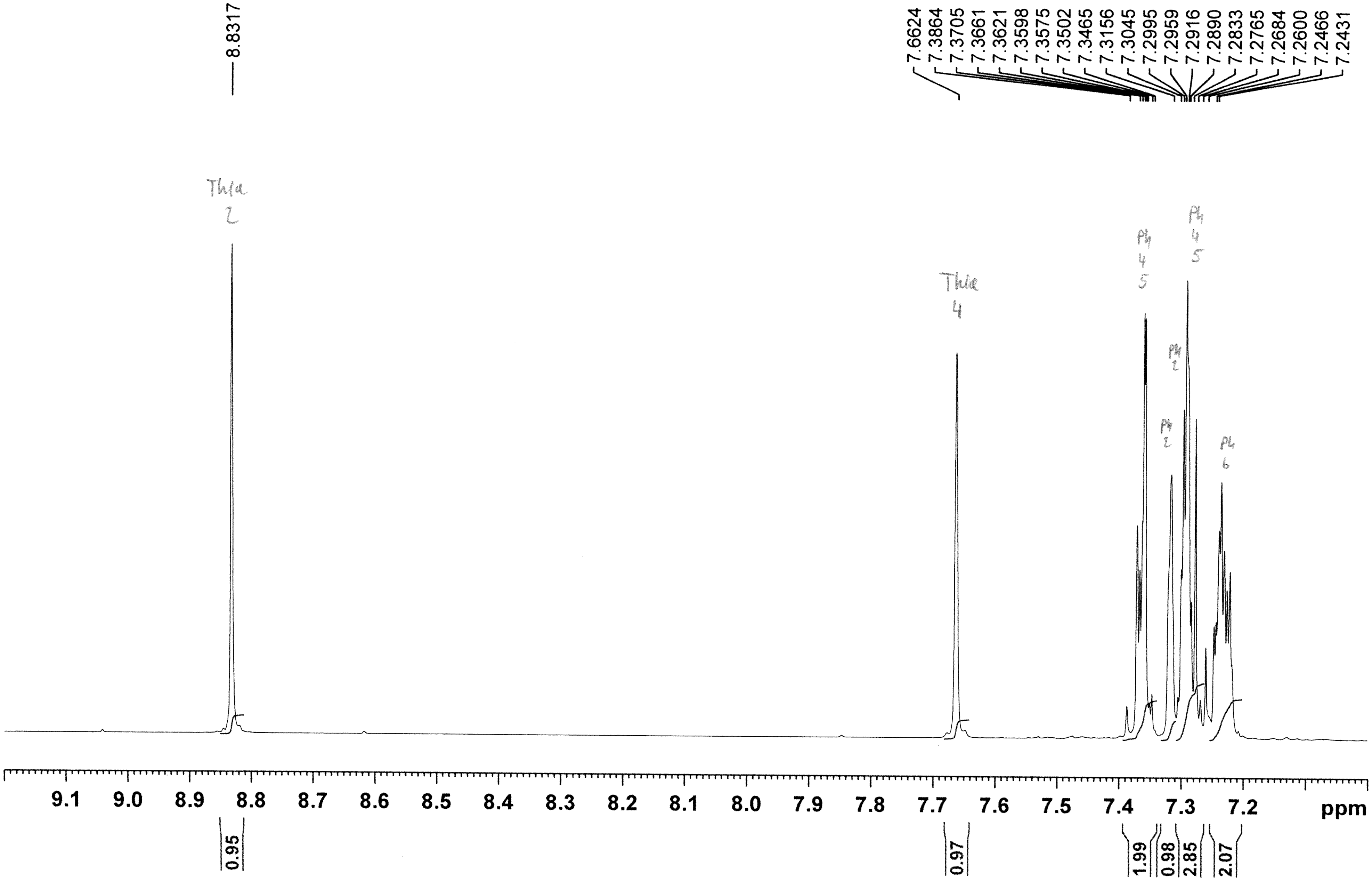

VD-US26 in cdcl3 (Proton) 26.2.2021

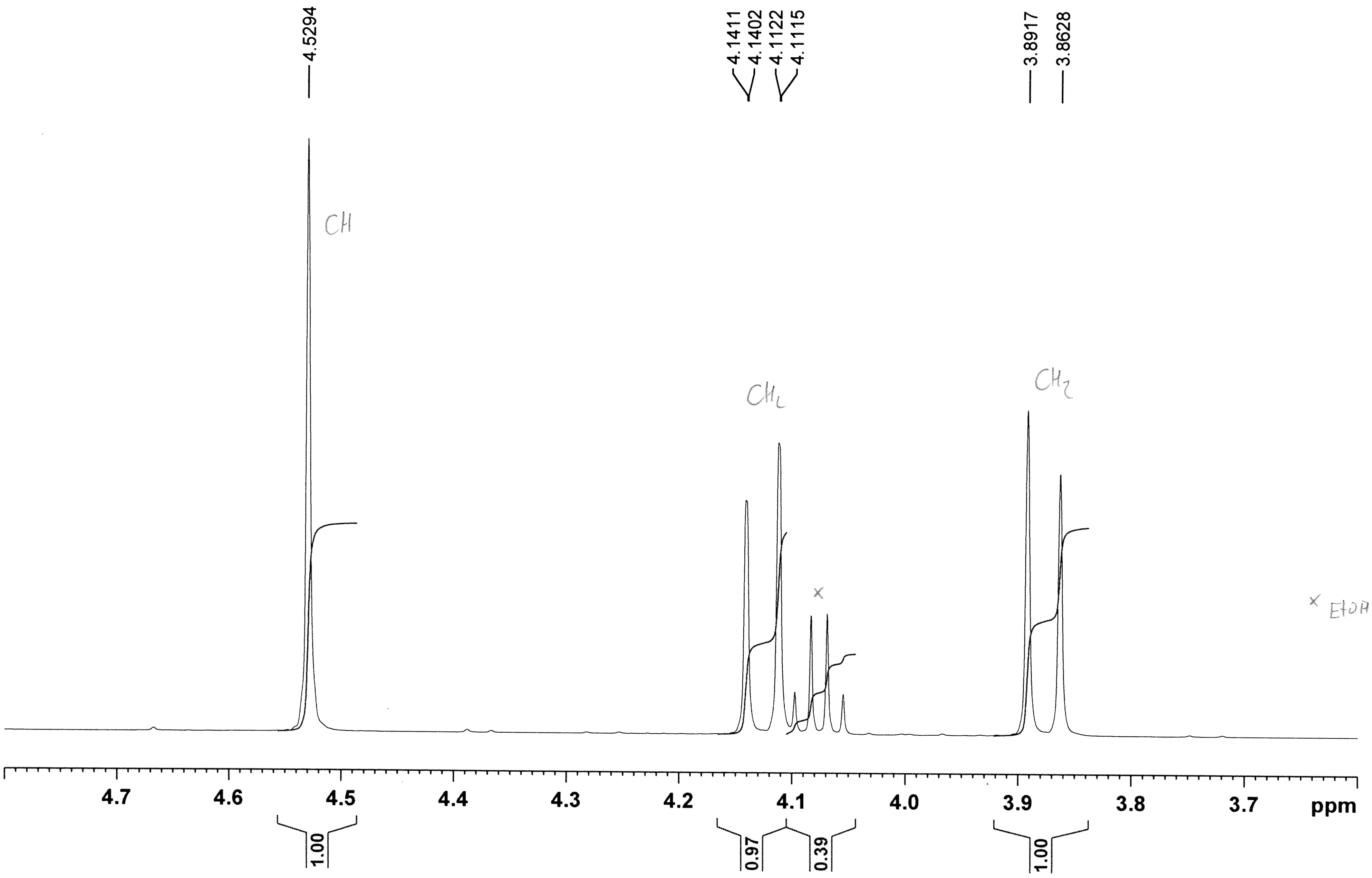

VD-US26 in cdcl3 (APT) 26.2.2021

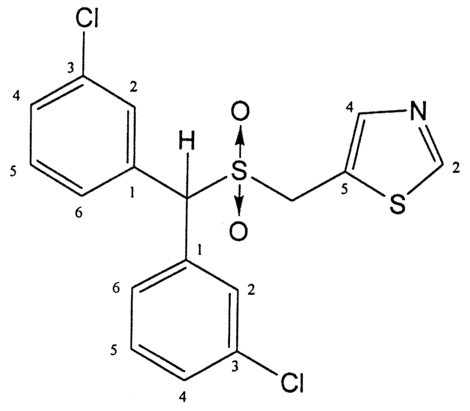

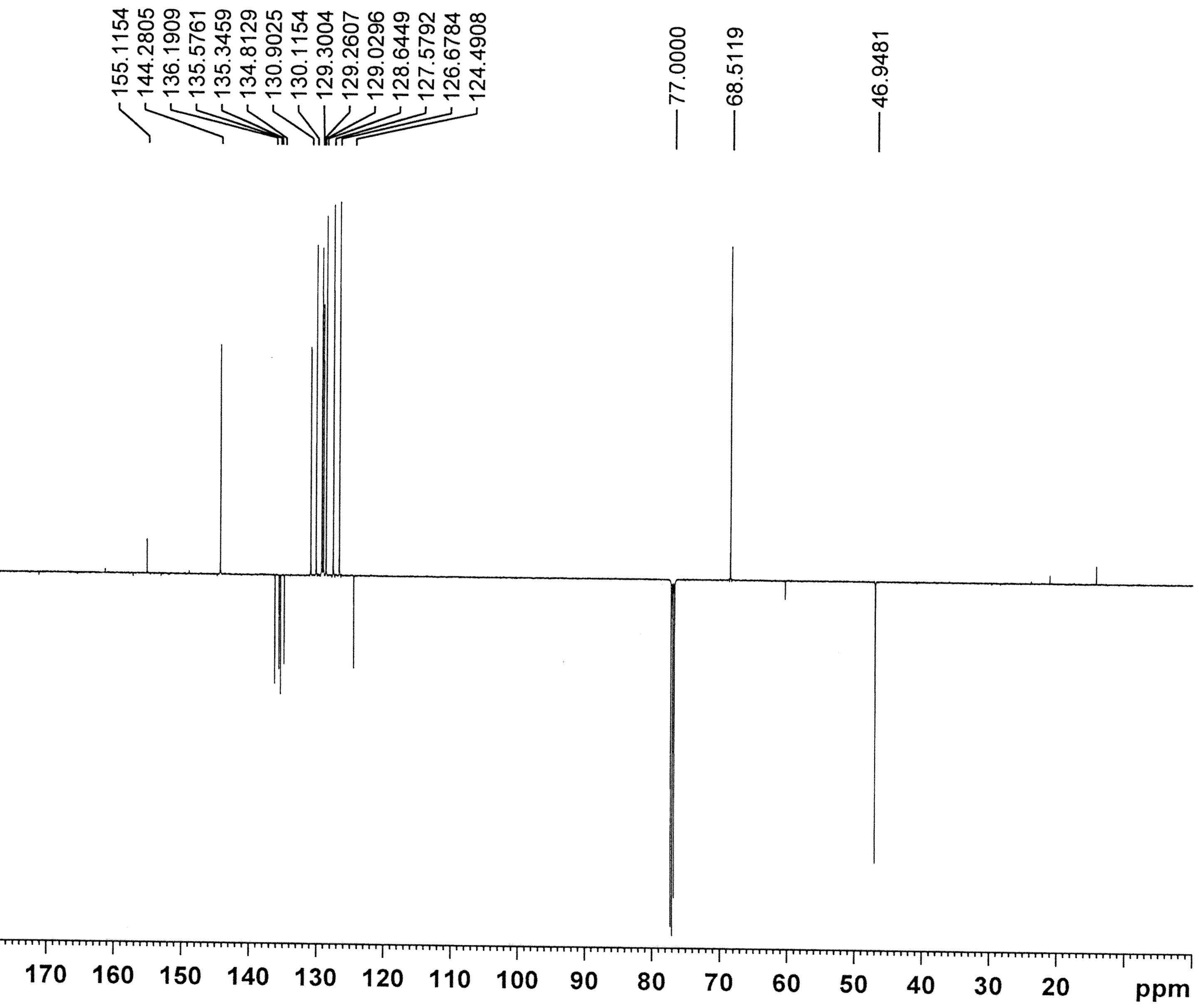

VD-US26 in cdcl3 (APT) 26.2.2021

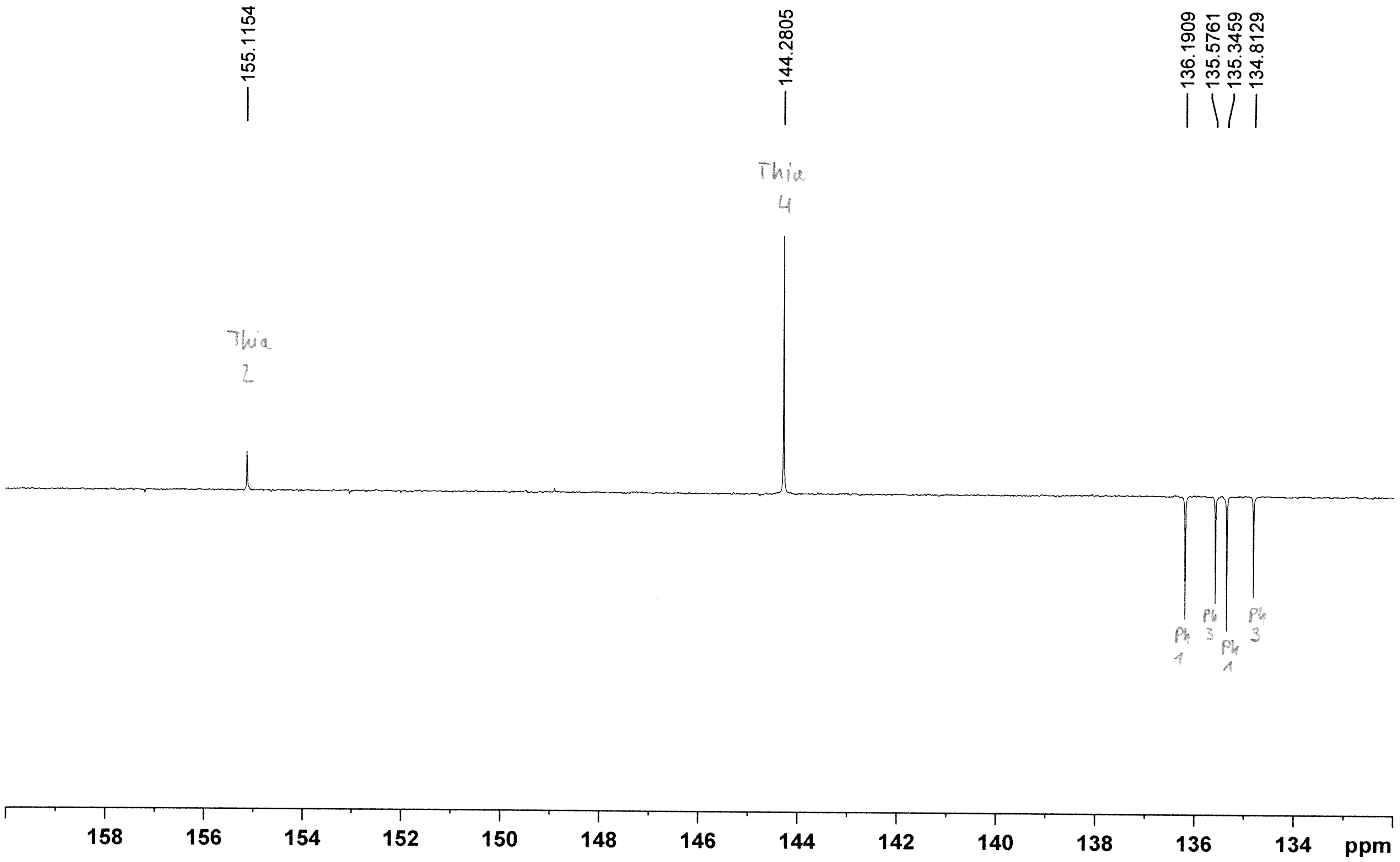

VD-US26 in cdcl3 (APT) 26.2.2021

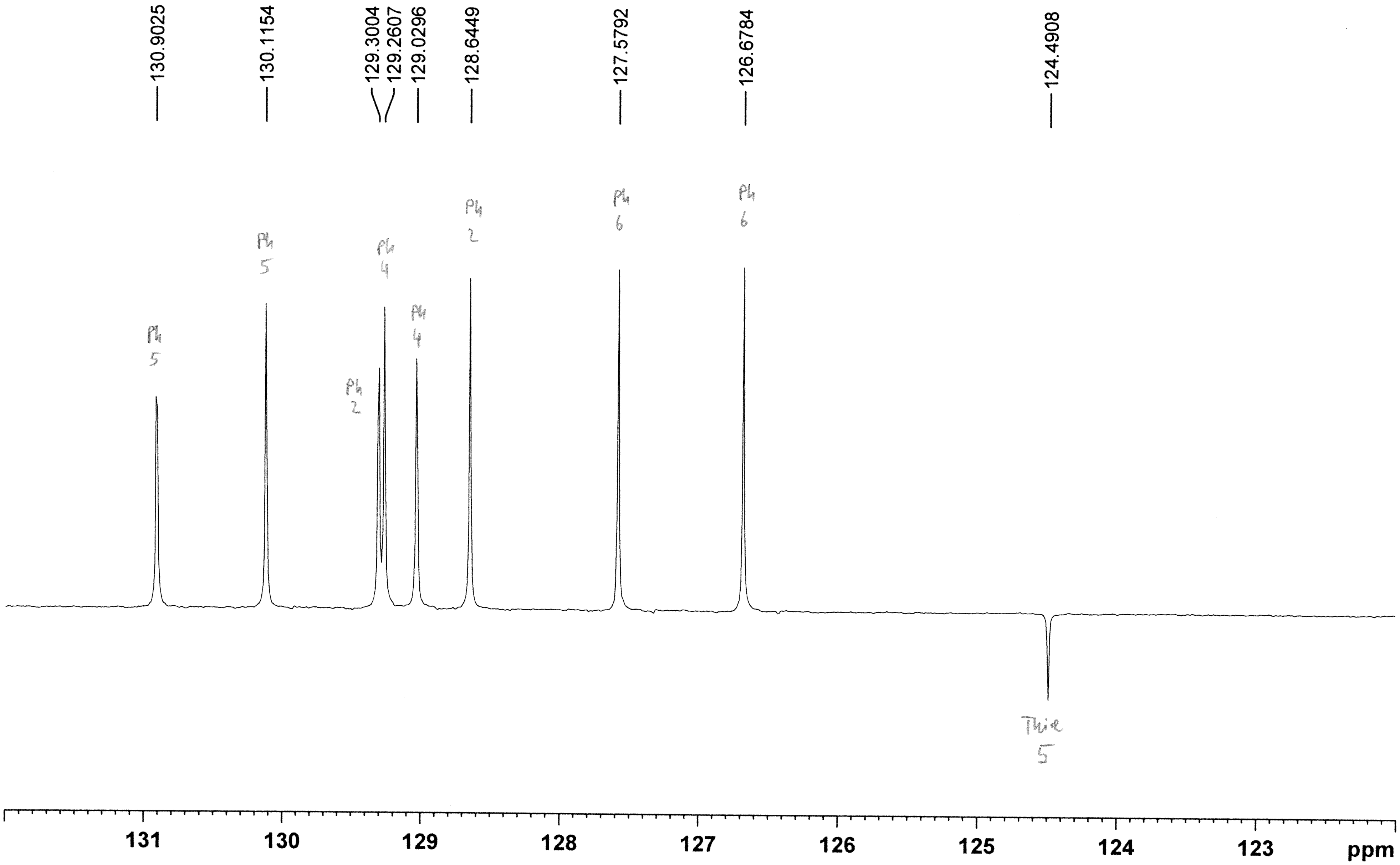

VD-US26 in cdcl3 (APT) 26.2.2021

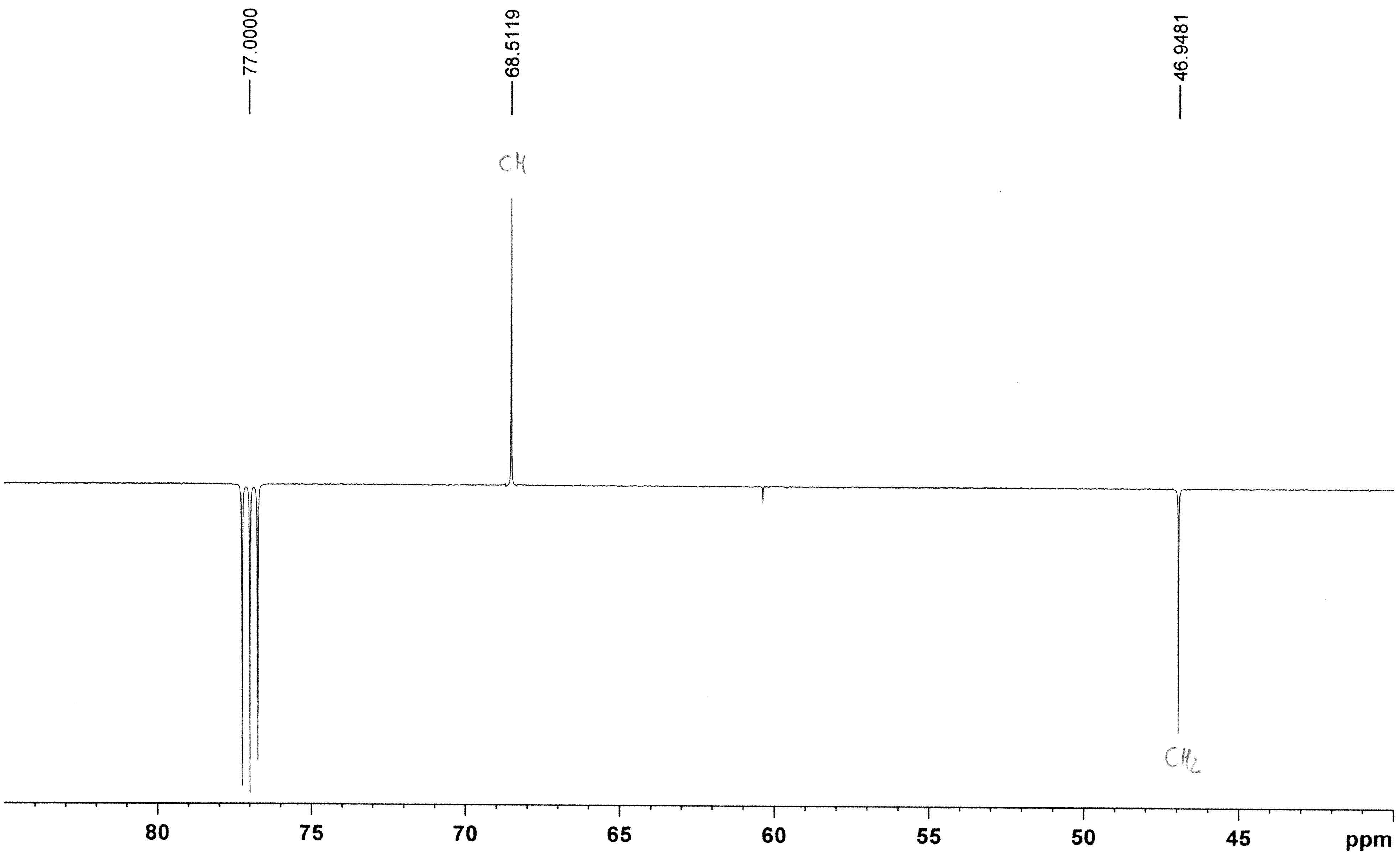

VD-US26 in cdcl3 (COSY) 26.2.2021

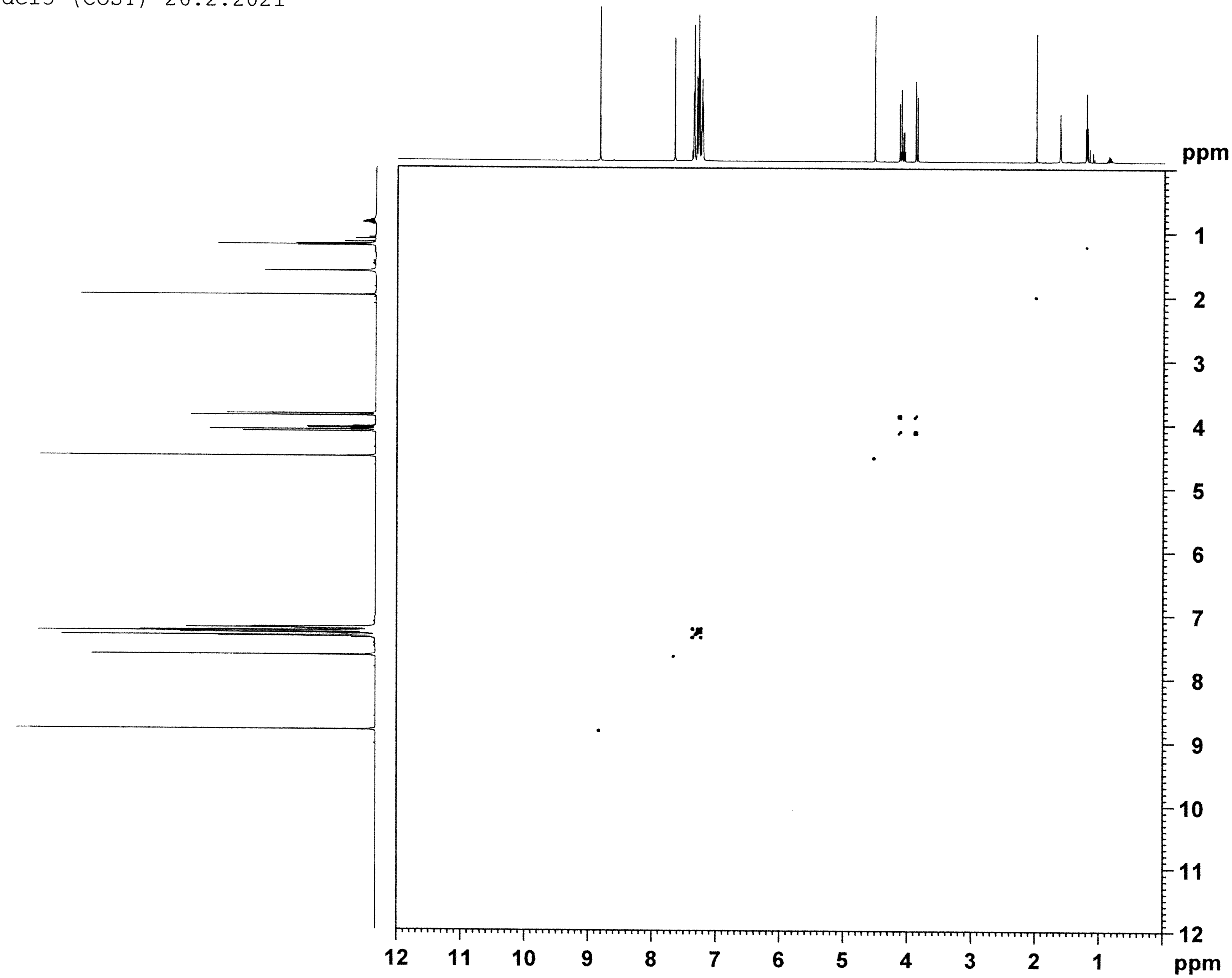

VD-US26 in cdcl3 (COSY) 26.2.2021

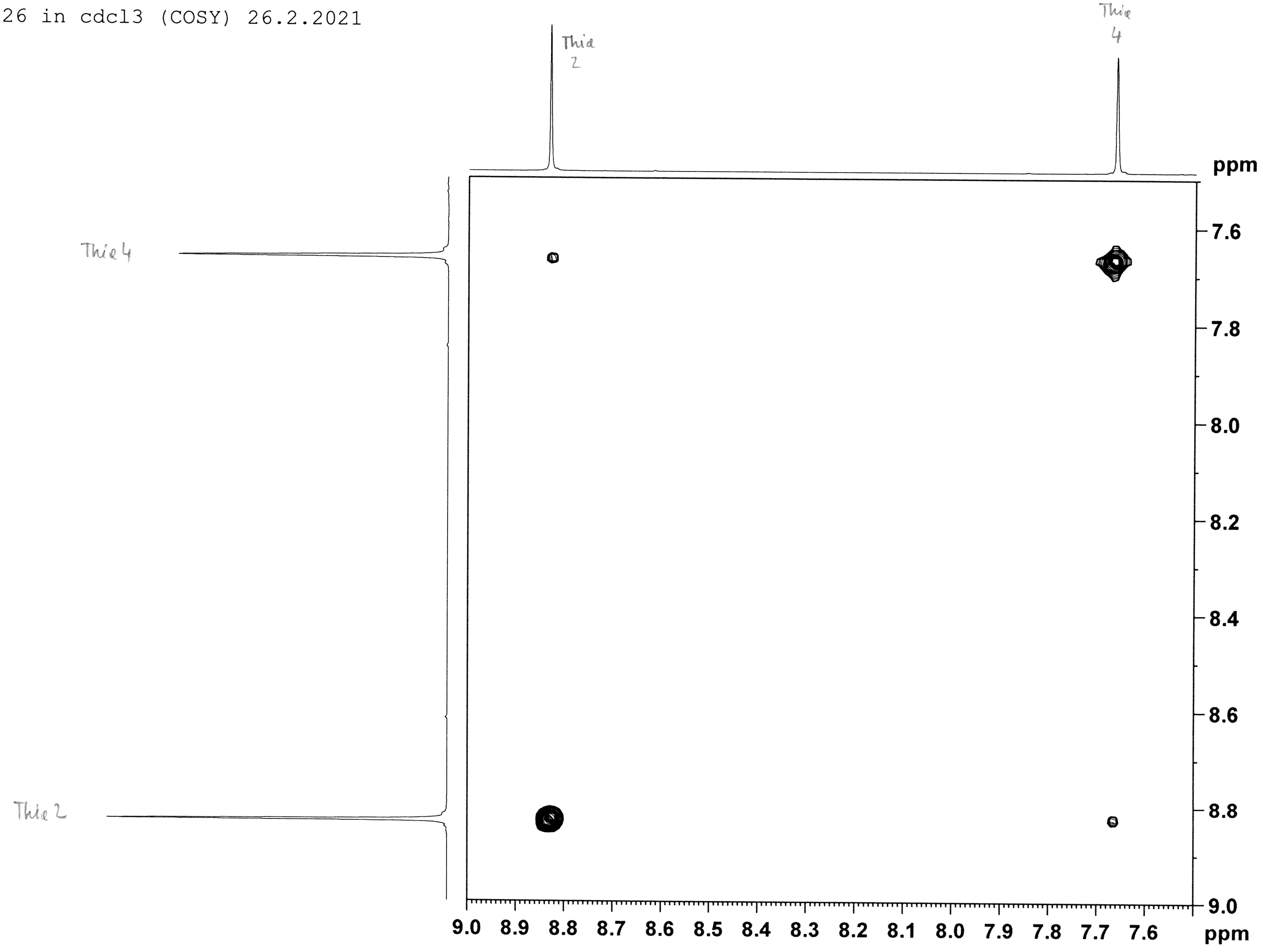

VD-US26 in cdcl3 (COSY) 26.2.2021

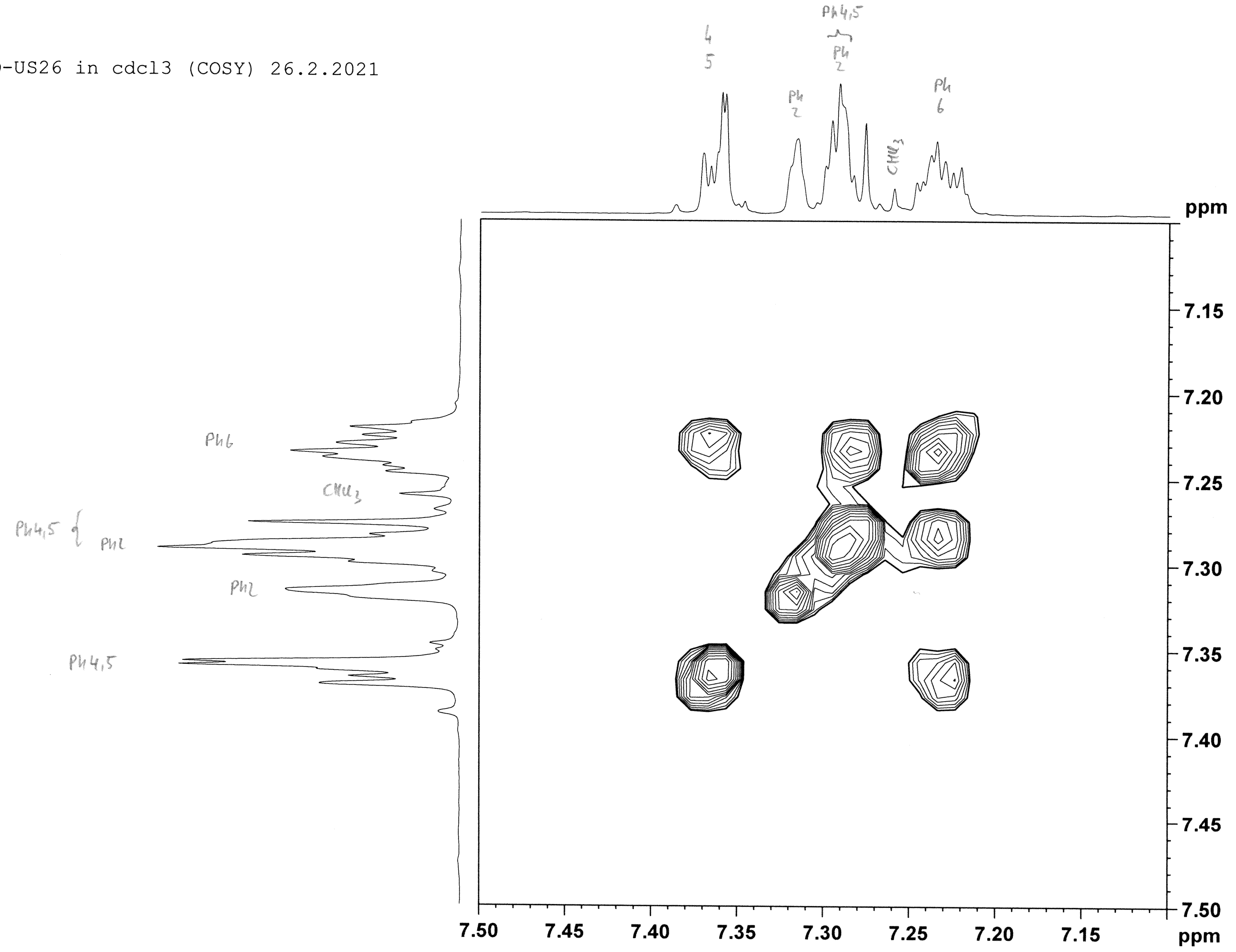

VD-US26 in cdcl3 (COSY) 26.2.2021

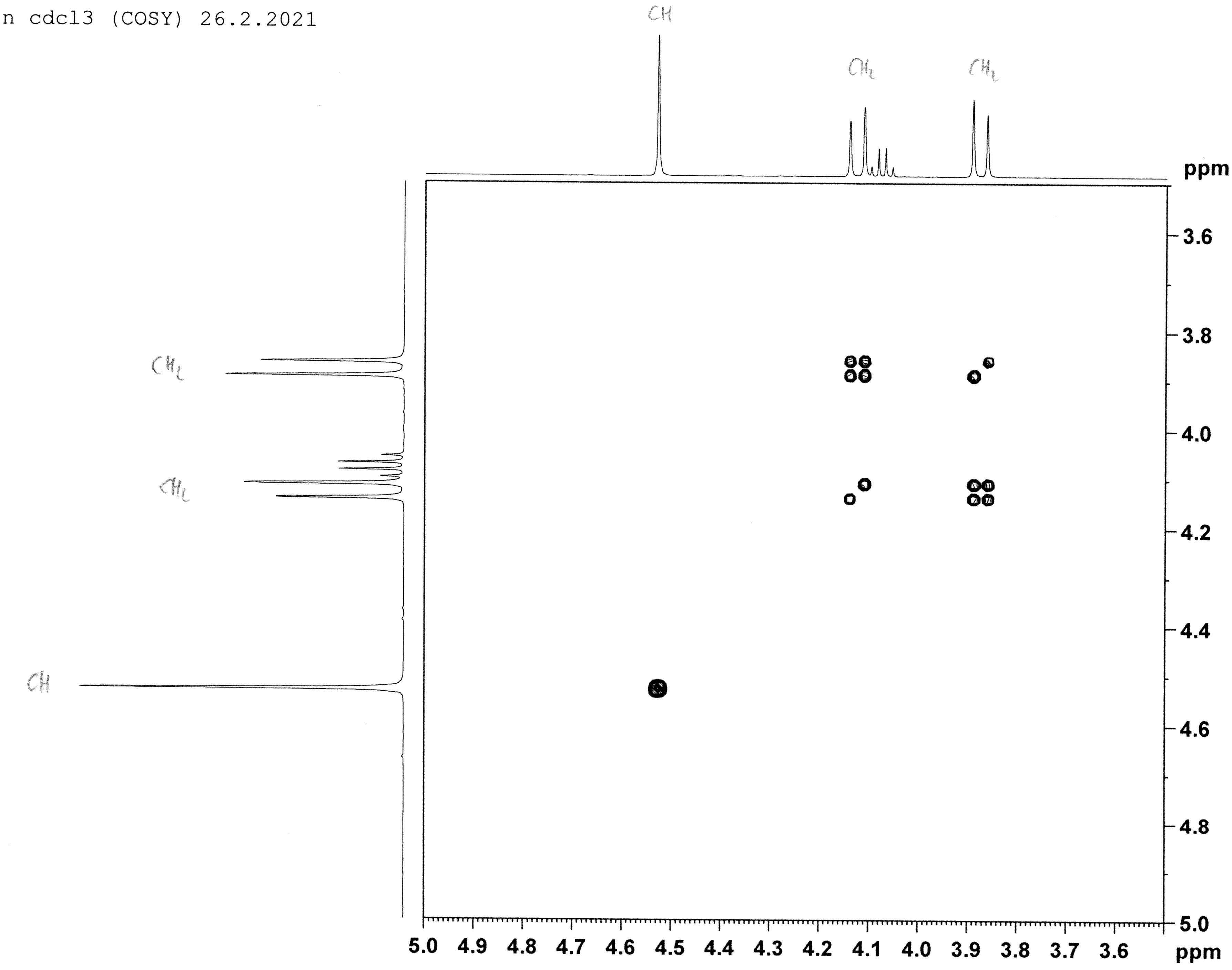

VD-US26 in cdcl3 (HSQC) 26.2.2021

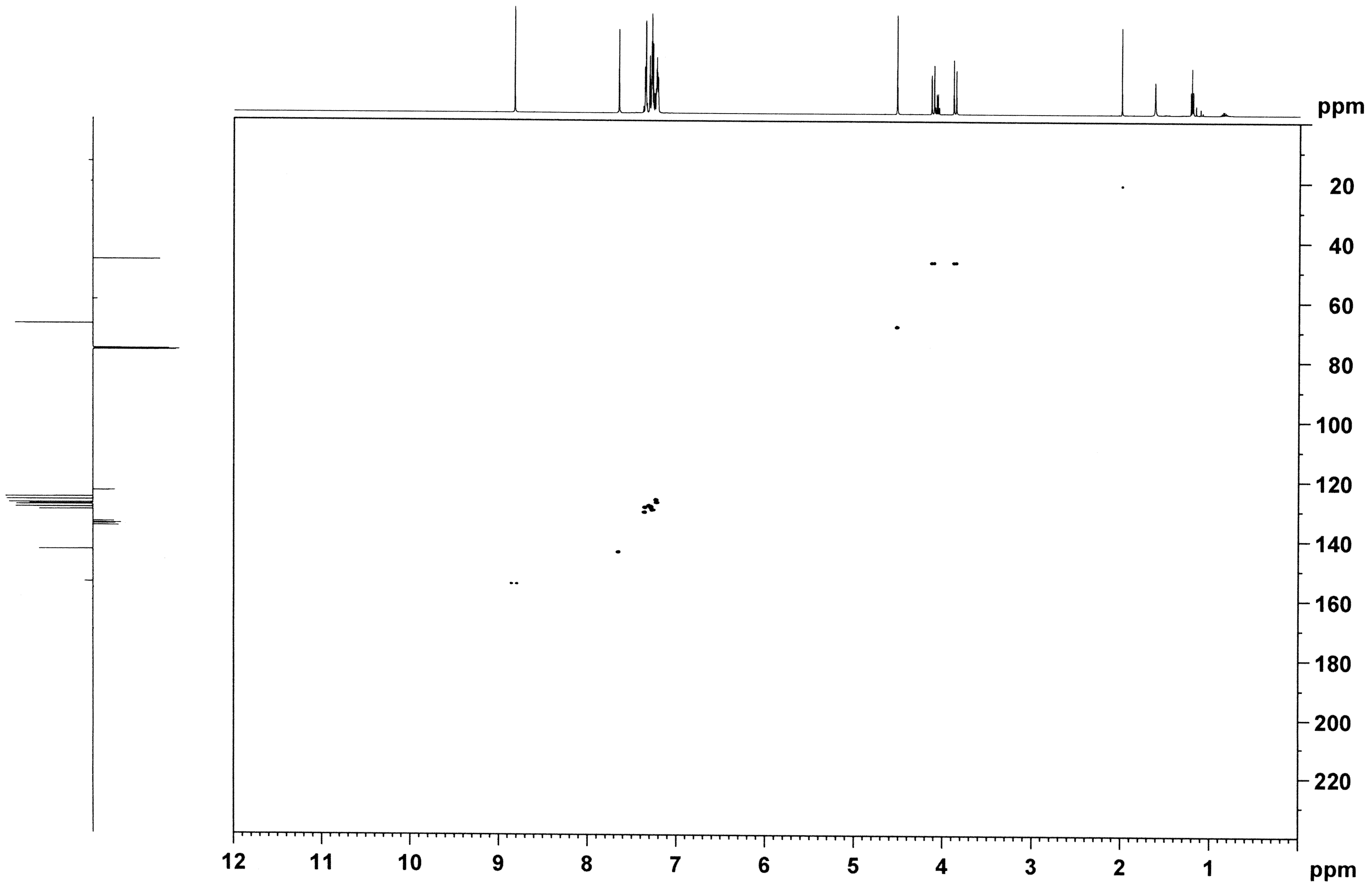

VD-US26 in cdcl3 (HSQC) 26.2.2021

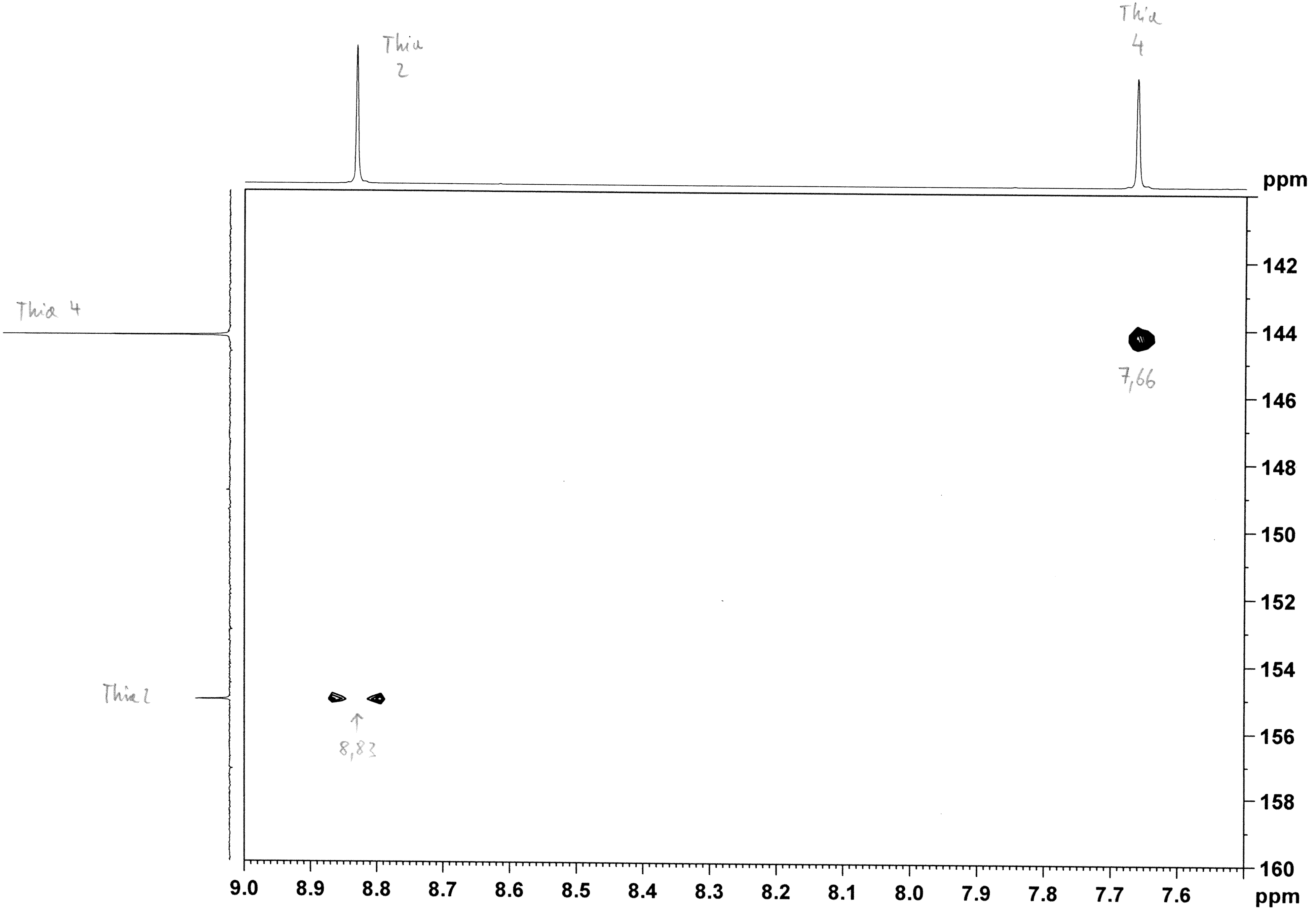

VD-US26 in cdcl3 (HSQC) 26.2.2021

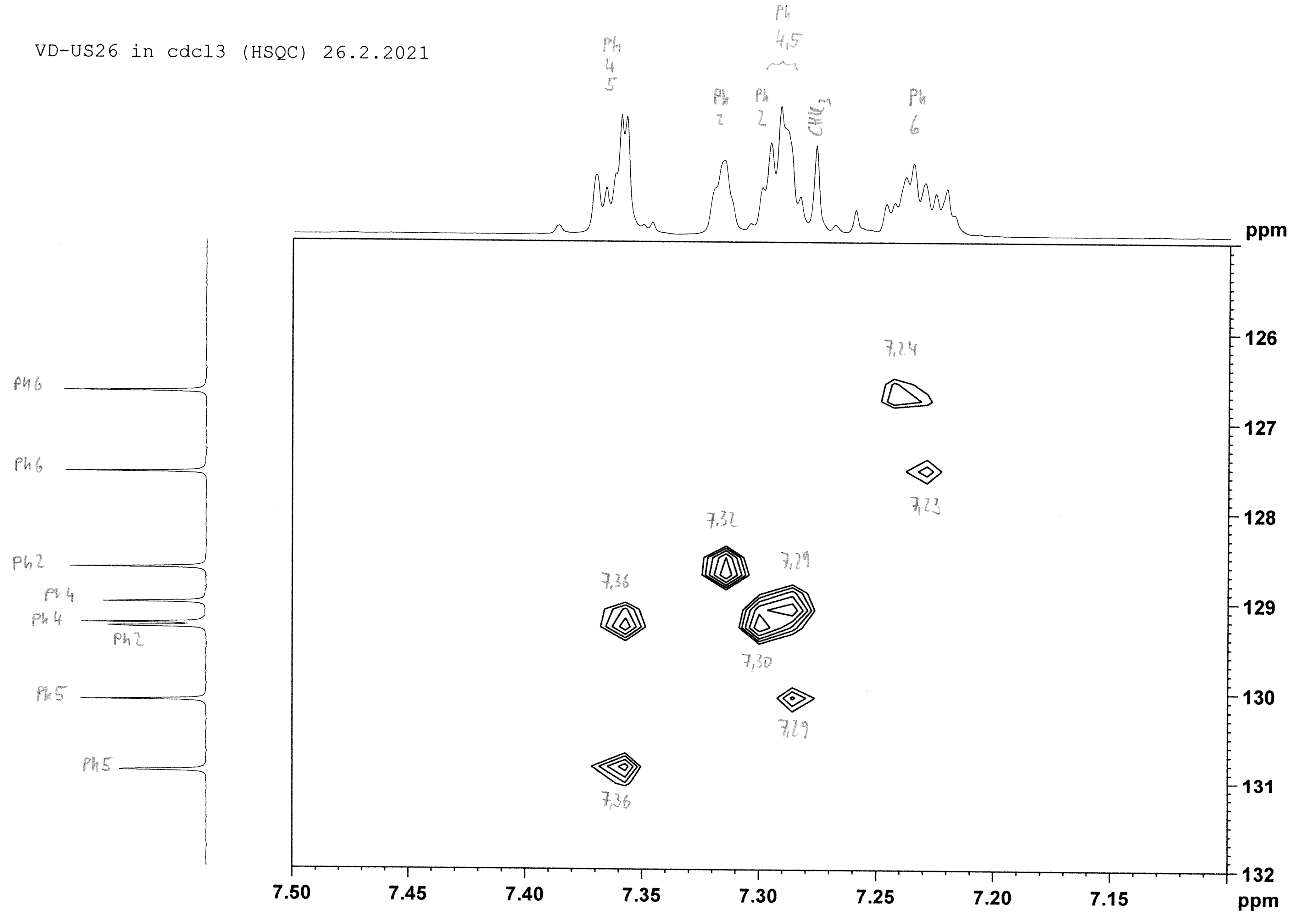

VD-US26 in cdcl3 (HSQC) 26.2.2021

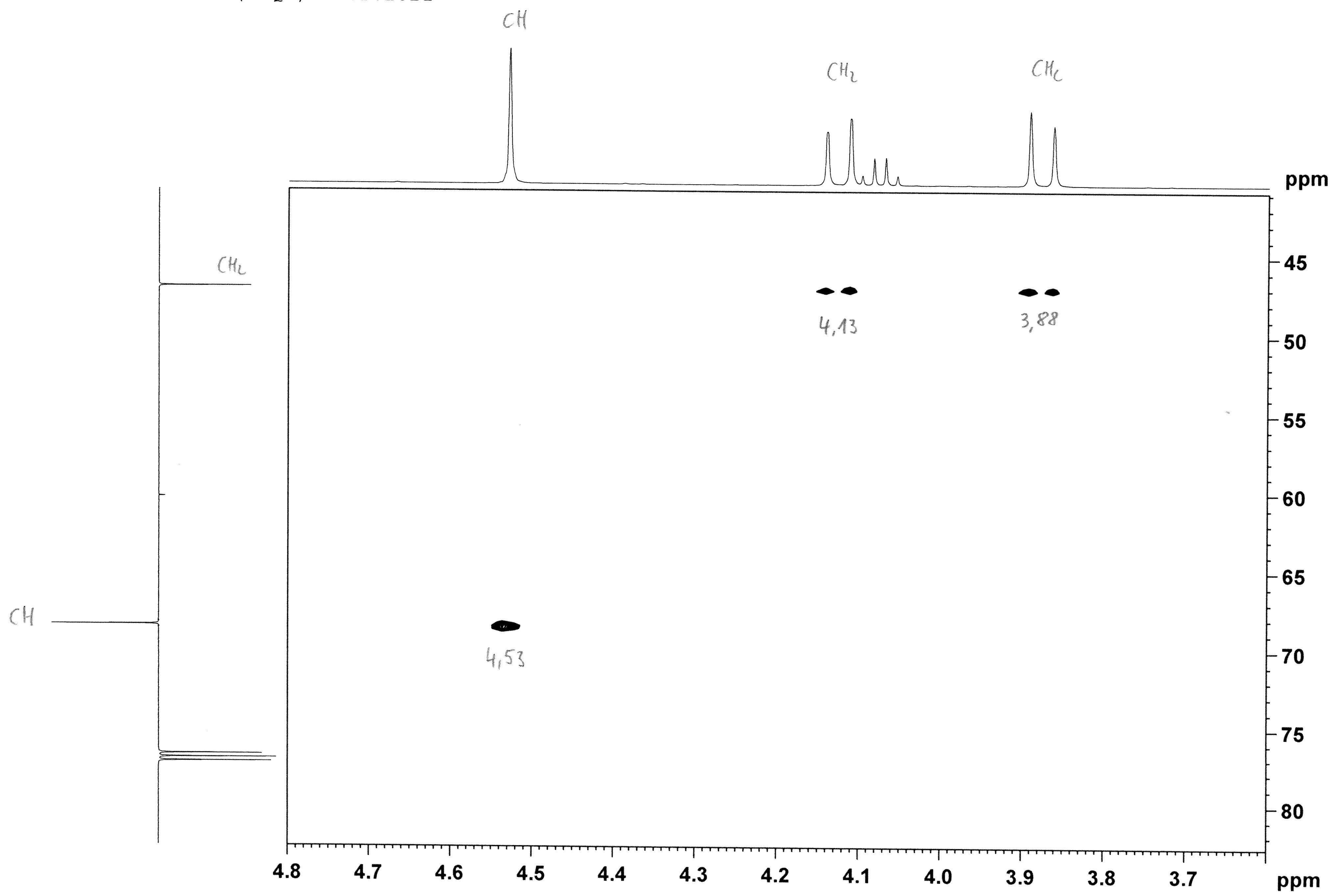

VD-US26 in cdcl3 (HMBC) 26.2.2021

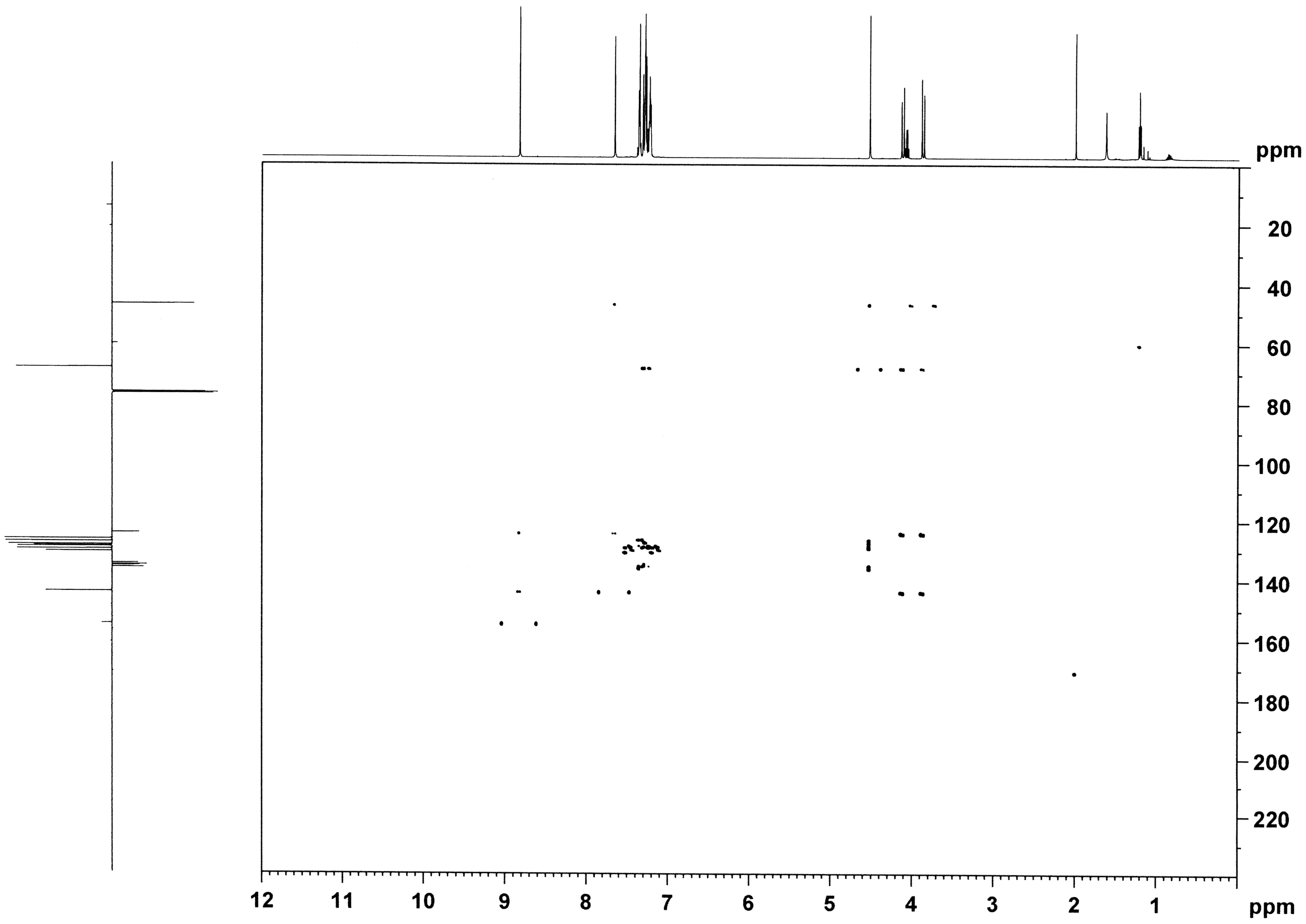

VD-US26 in cdcl3 (HMBC) 26.2.2021

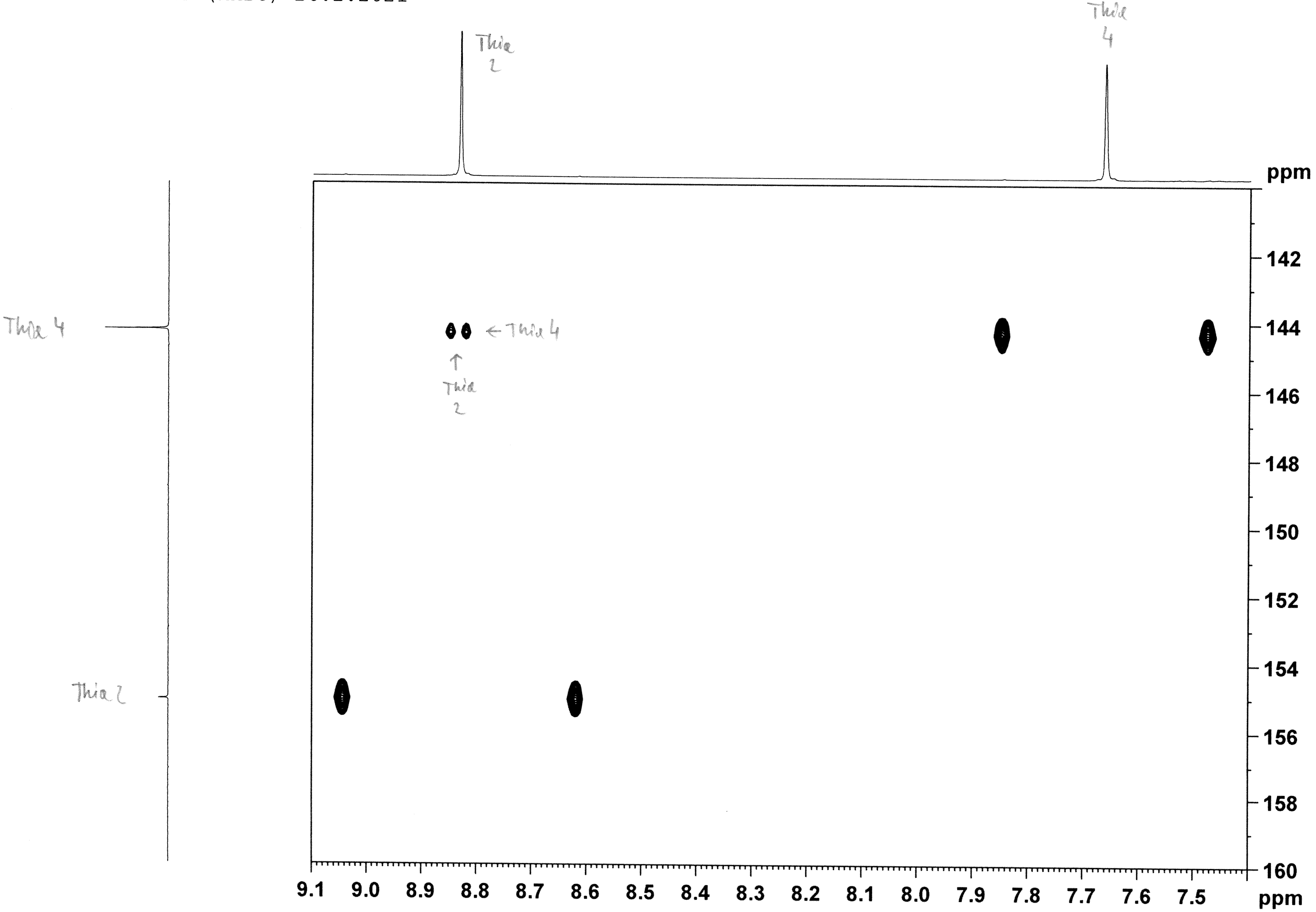

VD-US26 in cdcl3 (HMBC) 26.2.2021

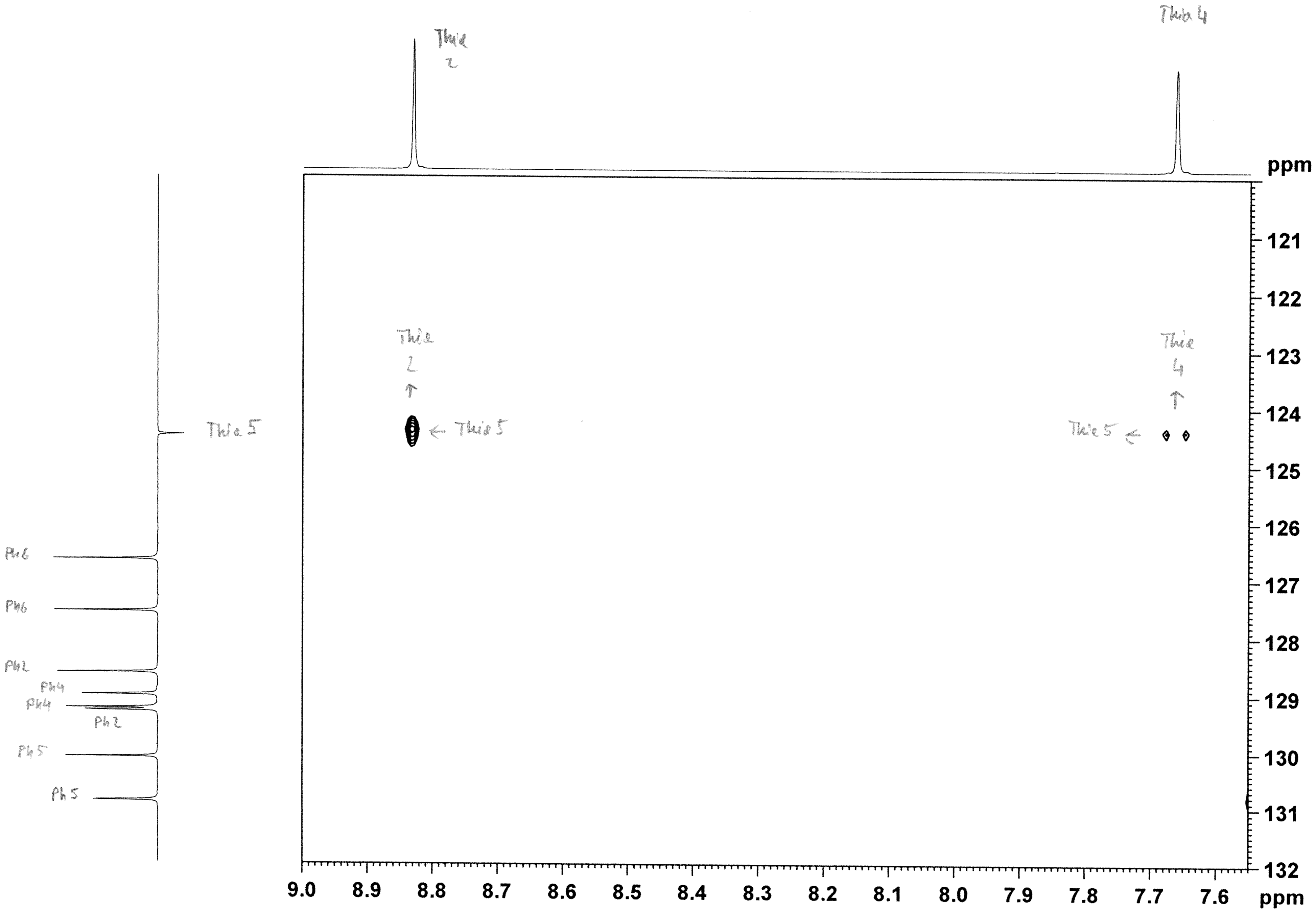

VD-US26 in cdcl3 (HMBC) 26.2.2021

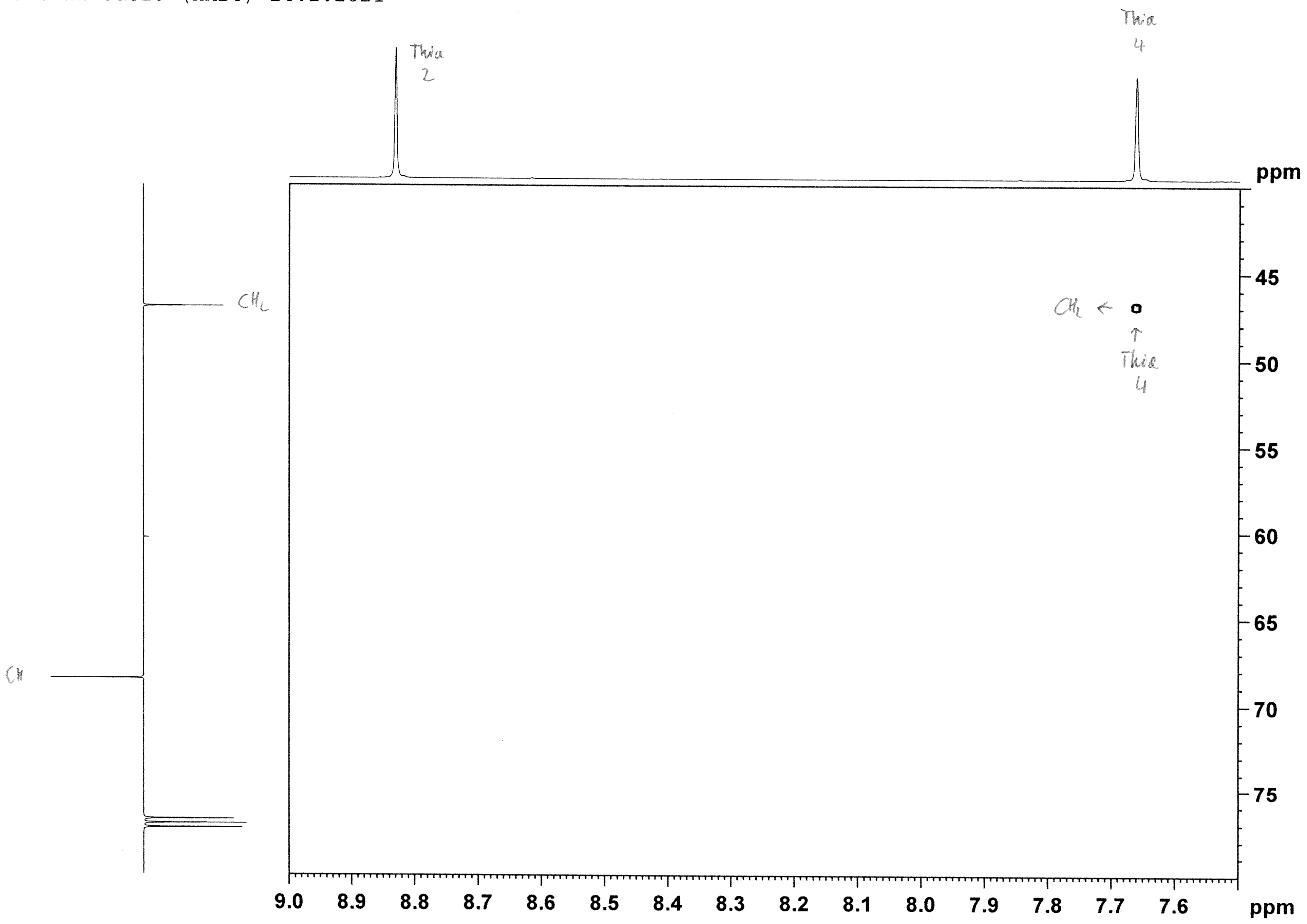

VD-US26 in cdcl3 (HMBC) 26.2.2021

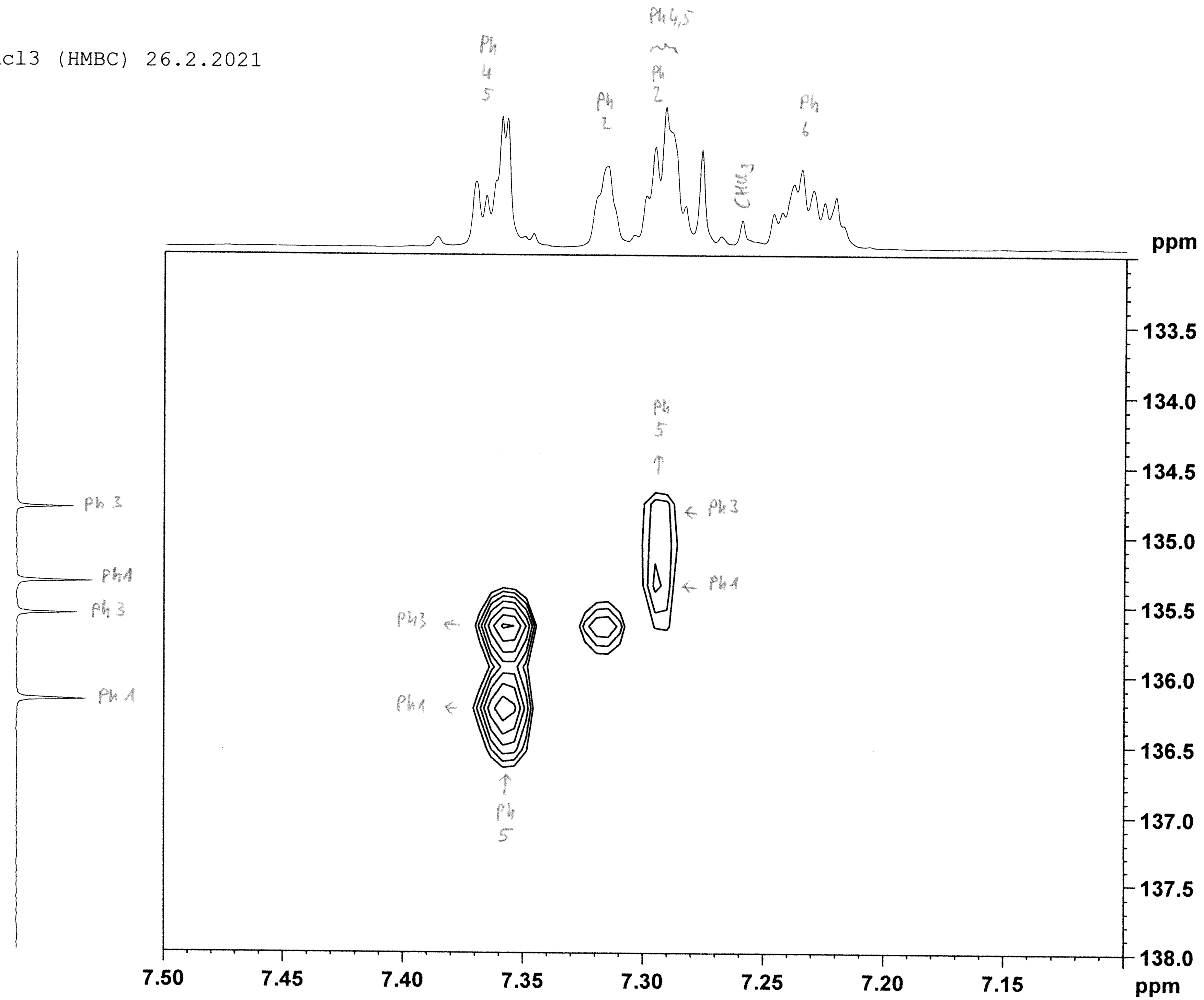

VD-US26 in cdcl3 (HMBC) 26.2.2021

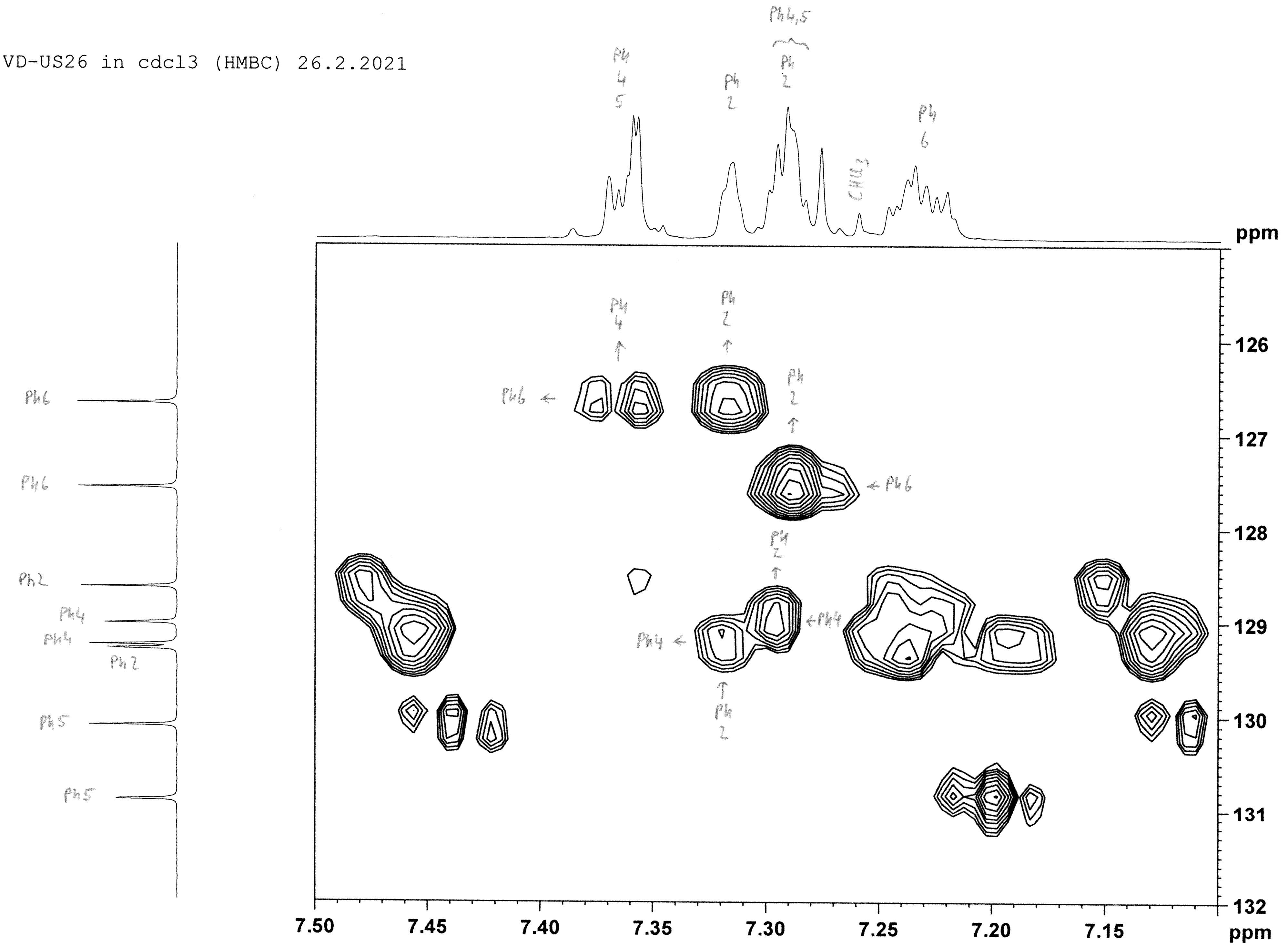

VD-US26 in cdcl3 (HMBC) 26.2.2021

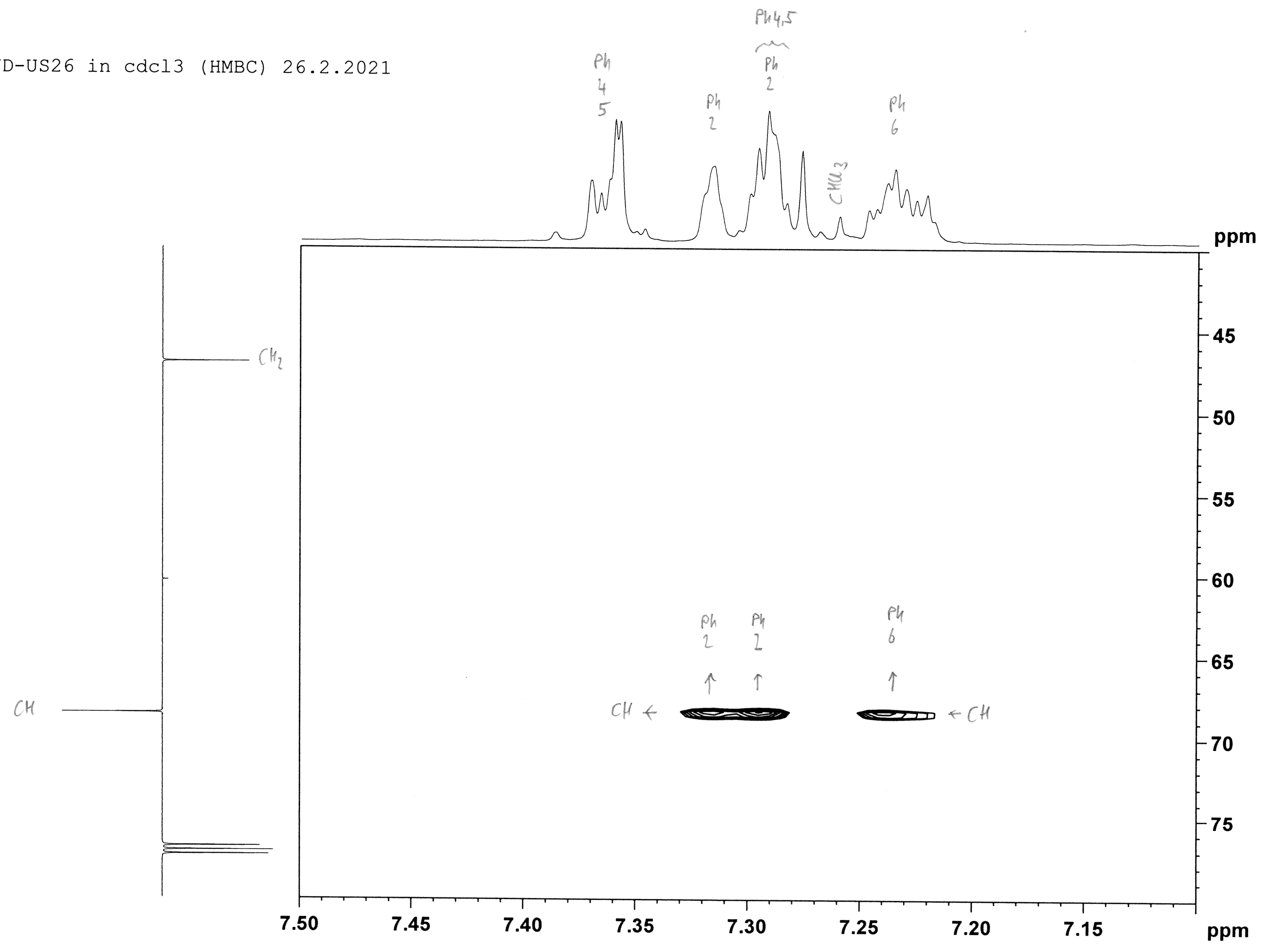

VD-US26 in cdcl3 (HMBC) 26.2.2021

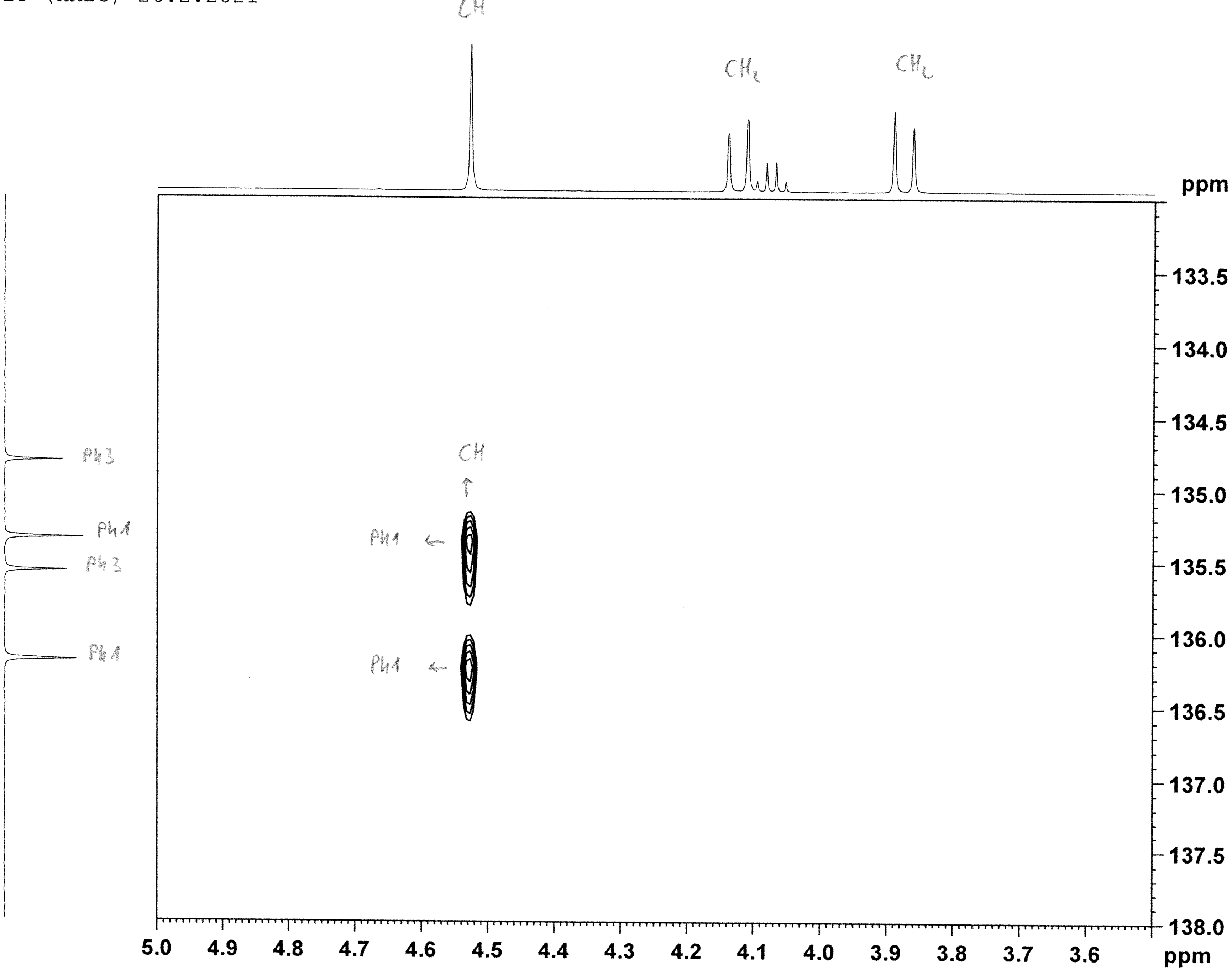

VD-US26 in cdcl3 (HMBC) 26.2.2021

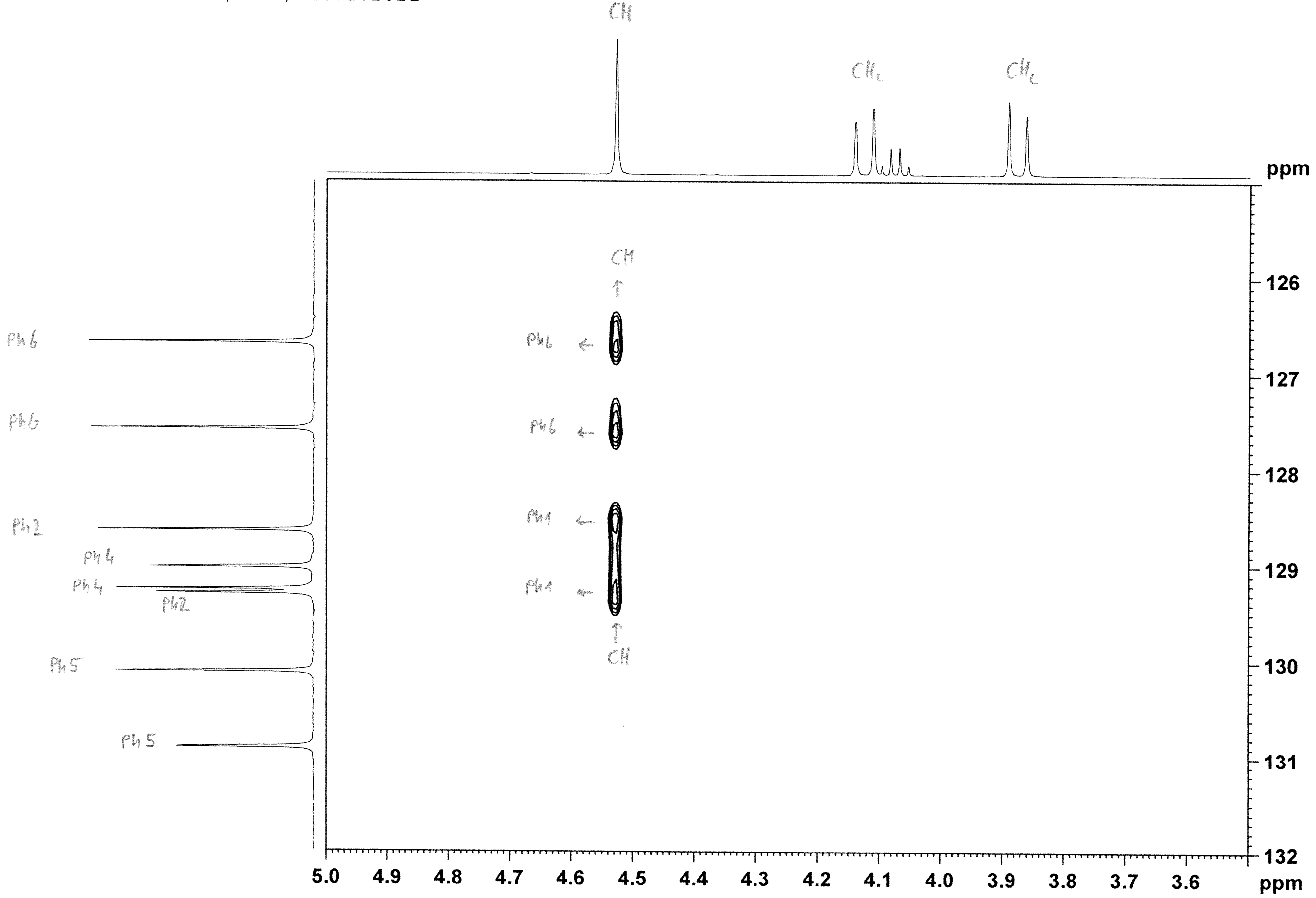

VD-US26 in cdcl3 (HMBC) 26.2.2021

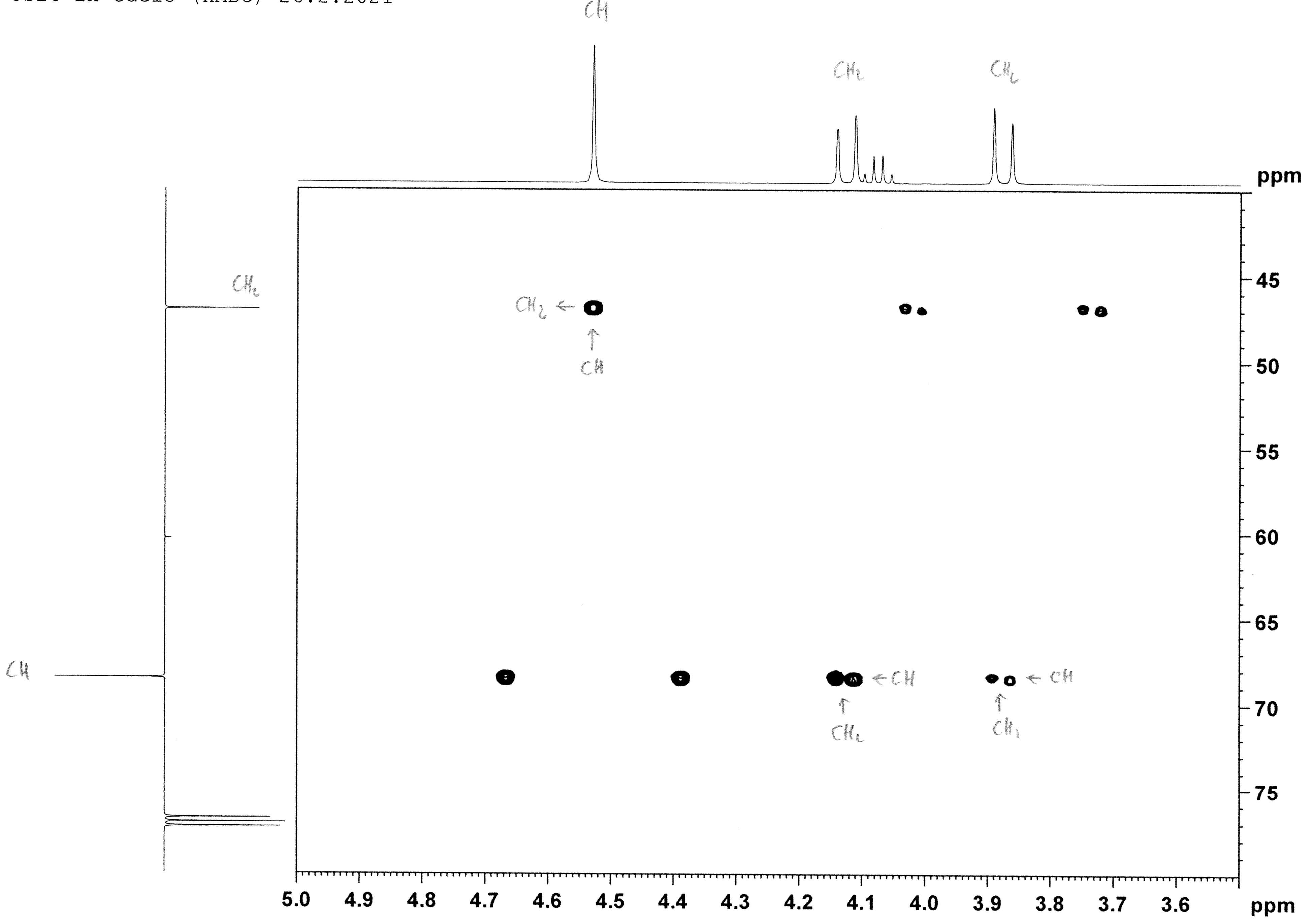

3.2. HRes MS spectra

Generic Display Report

Analysis Info

Analysis Name	D:\Data\Kalaba\78079000001.d	Acquisition Date	08/03/2021 15:32:45
Method	tune_low_MS_Service_03_21.m	Operator	msc
Sample Name	VD-US26	Instrument	maXis
Comment	Kalaba / Zehl<br>Ergebnis +/- 5 ppm<br>ACN / MeOH + 1% H2O		

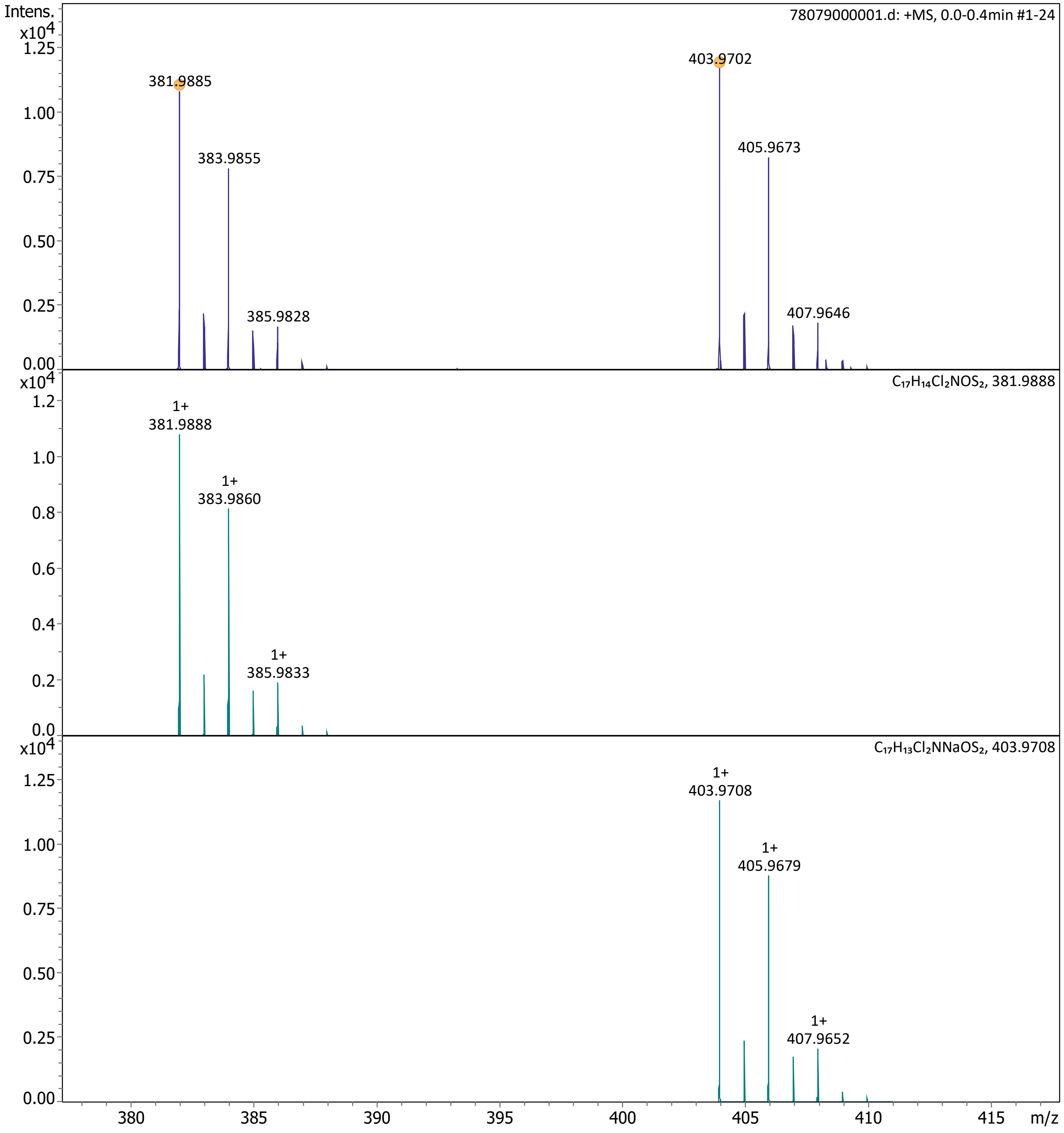

Mass Spectrum SmartFormula Report

Analysis Info

Analysis Name	D:\Data\Kalaba\78079000001.d	Acquisition Date	08/03/2021 15:32:45
Method	tune_low_MS_Service_03_21.m	Operator	msc
Sample Name	VD-US26	Instrument	maXis 255552.00016
Comment	Kalaba / Zehl<br>Ergebnis +/- 5 ppm<br>ACN / MeOH + 1% H2O		

Acquisition Parameter

Source Type	ESI	Ion Polarity	Positive	Set Nebulizer	0.4 Bar
Focus	Not active	Set Capillary	4200 V	Set Dry Heater	180 Â°C
Scan Begin	80 m/z	Set End Plate Offset	-500 V	Set Dry Gas	4.0 l/min
Scan End	1900 m/z	Set Charging Voltage	0 V	Set Divert Valve	Source
		Set Corona	0 nA	Set APCI Heater	0 Â°C

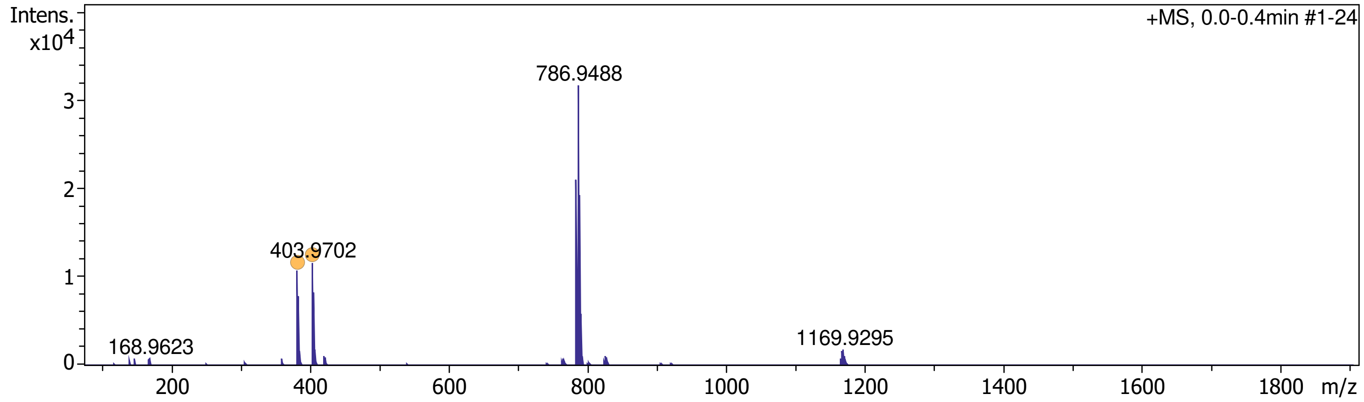

Meas. m/z	#	Ion Formula	Score	m/z	err [mDa]	err [ppm]	mSigma	rdb	eÂ¯ Conf	N-Rule
381.9885	1	C17H14Cl2NOS2	100.00	381.9888	0.4	1.0	13.6	21.0	even	ok
	2	C17H6Cl2N5O2	47.37	381.9893	0.8	2.2	30.0	23.0	even	ok
	3	C16H10Cl2NO6	55.55	381.9880	-0.5	-1.3	30.7	18.0	even	ok
	4	C13H2Cl2N11	21.40	381.9866	-1.8	-4.8	36.3	24.0	even	ok
	5	C15H13ClN3OS3	1.11	381.9904	1.9	5.0	111.6	20.0	even	ok
	6	C12H7Cl3N9	3.41	381.9885	-0.0	-0.0	113.7	22.0	even	ok
	7	C18H9ClN3OS2	0.58	381.9870	-1.5	-3.8	129.3	23.0	even	ok
	8	C14H9ClN3O6S	0.07	381.9895	1.0	2.7	164.3	17.0	even	ok
	9	C22H5ClNO4	0.01	381.9902	1.7	4.4	179.7	24.0	even	ok
	10	C18HClN7O2	0.02	381.9875	-1.0	-2.6	183.8	25.0	even	ok
403.9702	1	C17H13Cl2NNaOS2	100.00	403.9708	0.5	1.4	20.1	21.0	even	ok
	2	C16H9Cl2NNaO6	85.04	403.9699	-0.3	-0.8	25.3	18.0	even	ok
	3	C17H5Cl2N5NaO2	59.25	403.9713	1.0	2.5	25.5	23.0	even	ok
	4	C13HCl2N11Na	34.58	403.9686	-1.7	-4.1	30.8	24.0	even	ok
	5	C12H6Cl3N9Na	3.03	403.9704	0.2	0.4	118.8	22.0	even	ok
	6	C11H10Cl3N5NaO4	1.50	403.9691	-1.2	-2.9	122.2	17.0	even	ok
	7	C18H8ClN3NaOS2	1.12	403.9690	-1.3	-3.2	123.5	23.0	even	ok
	8	C11H18Cl3NNaO3S2	0.27	403.9686	-1.6	-4.1	145.8	15.0	even	ok
	9	C14H8ClN3NaO6S	0.12	403.9715	1.2	3.0	157.2	17.0	even	ok
	10	C11ClN13NaS	0.14	403.9701	-0.1	-0.3	163.0	23.0	even	ok
	11	C22H4ClNNaO4	0.02	403.9721	1.9	4.6	173.5	24.0	even	ok
	12	C18ClN7NaO2	0.04	403.9694	-0.8	-2.0	177.3	25.0	even	ok
	13	C11H11Cl4N7Na	0.02	403.9722	2.0	4.9	180.6	20.0	even	ok

3.3. General purity determined by HPLC on a C18 column

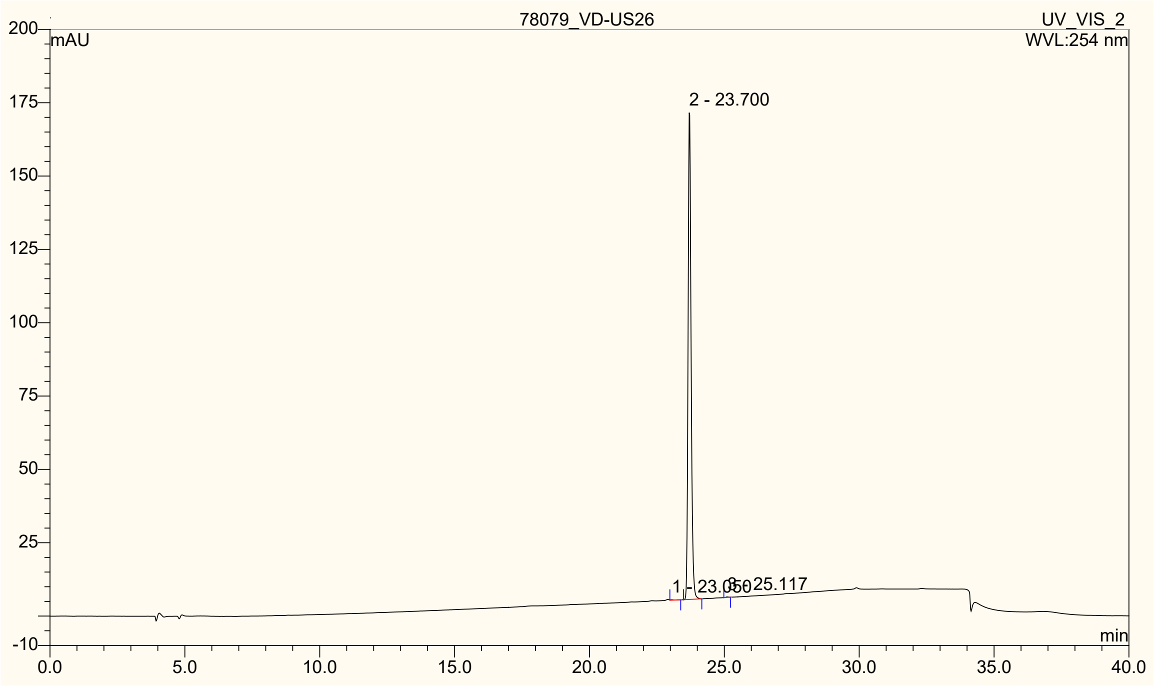

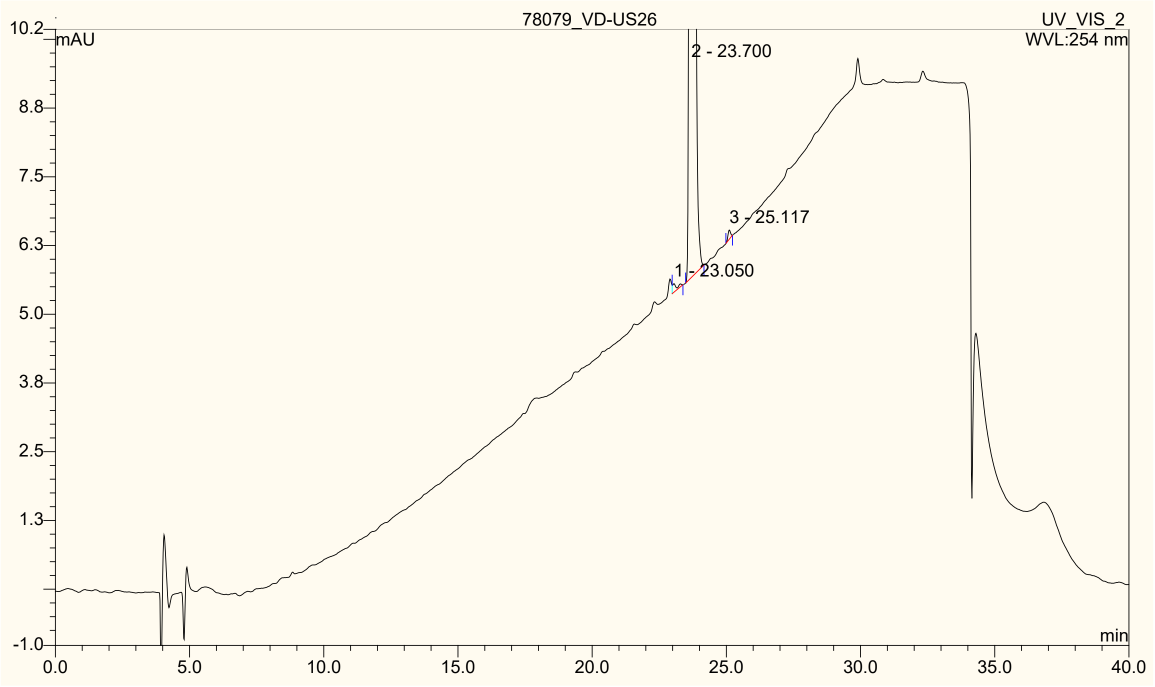

Retention Time: **23.70 min**

Relative Peak Area: **99.78 %**

3.4. Chiral purity

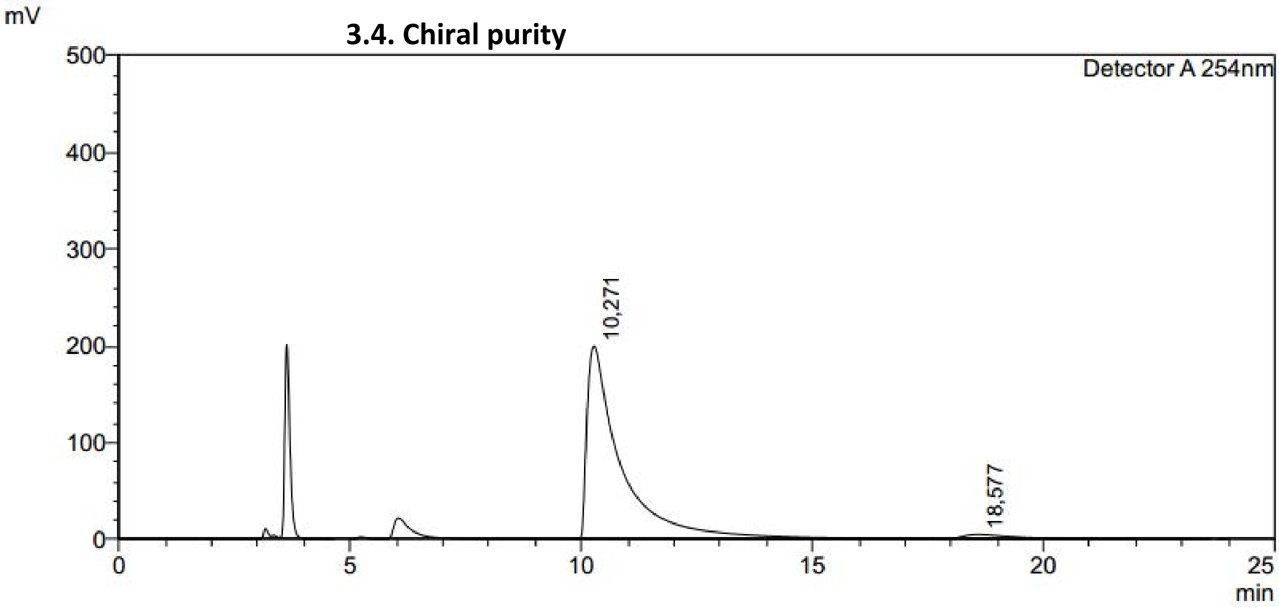

<Peak Table>

Detector A 254nm

Peak#	Ret. Time	Area	Area%
1	10,271	10453608	96,427
2	18,577	387349	3,573
Total		10840957	100,000

4. Absolute configuration attribution for *(S)*- and *(R)*-MK-26 gained via racemic oxidation and chiral resolution

Title:

VCD Absolute Configuration Determination Report

GENERAL INFORMATION	
**Customer**	Vienna
**Sales Order Number**	2021-91 LSV-BTE
**Sample code (BT ref.)**	**MK-26 P1 / MK-26 P2**
**Sample description (Customer ref.)**	**MK-26 P1 / MK-26 P2**
**VCD-spectrometer**	ChiralIR w/ Dual*PEM*
**Report prepared by: (name / signature as needed)**	Jordan Nafie
**Report validated and signed by**	Rina K Dukor
**Date**	December 20, 2021
**RESULTS**	
Absolute Configuration of MK-26 P2 is (R)<br>Absolute Configuration of MK-26 P1 is (S)	Confidence Level: **98%**
**MEASUREMENT PARAMETERS**	
**Concentration**	12mg / 150uL
**Solvent**	$CDCl_3$
**Instrument Resolution**	4 cm^{-1}
**PEM setting**	1400 cm^{-1}
**Number of scans/Measurement time**	12 hours per enantiomer
**Sample cell**	BaF_2
**Path length**	100 µm
**CALCULATION DETAILS**	
**Molecular Mechanics Force Field**	**MMFF94 (Compute VOA)**
**DFT Software version**	**Gaussian '09**
**Number of conformers used for Boltzmann sum**	**37 (cc-pVTZ / B3PW91)**
**Methodology and basis sets for DFT calculations**	**6-31G(d), cc-pVTZ / B3LYP, B3PW91 / CPCM (Chloroform)**
**Enantiomer used for calculation**	**R (Sulfur)**
**Total calculated conformers**	**150 6-31G(d) / 68 cc-pVTZ**
**Number of low-energy conformations shown in report**	**4**

COMMENTS

The confidence level is a measure of the degree of congruence between a calculated and measured spectrum. If identical spectra are being compared the confidence level is 100%. The confidence level (CL) is not the likelihood that the assignment is correct. Rather it's a measure of quality or degree of agreement between calculated and measured spectra. With a CL of 98% for this molecule, the visual agreement between measured and calculated spectra is excellent – this is a very high confidence assignment. Four different calculations were performed, two functionals (B3LYP and B3PW91) each with two basis sets (6-31G(d) and cc-pVTZ) – the CPCM solvent shell method was employed in each cases. While all four indicated the same result for stereochemistry – the best overall match was cc-pVTZ / B3PW91. The chiral center in this molecule is the sulfur atom of the sulfoxide functional group.

Title:

VCD Absolute Configuration Determination Report

Structure of MK-26 P2:

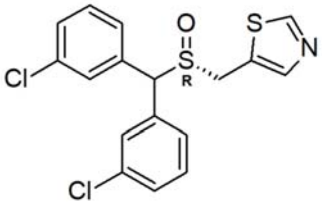

MK-26 P2

Structure of MK-26 P1:

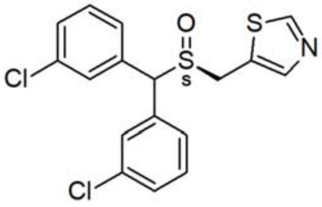

MK-26 P1

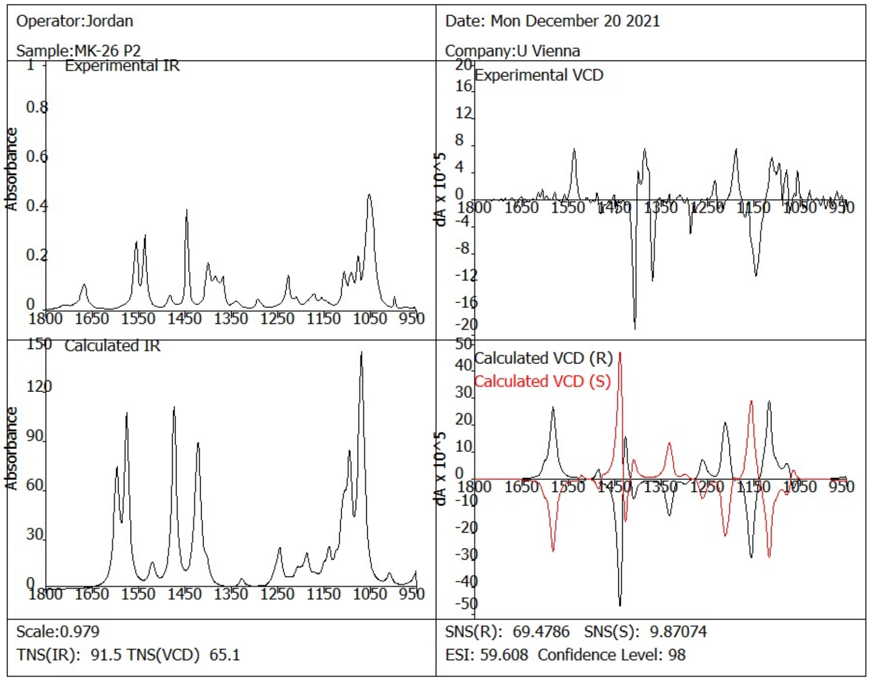

CompareVOA Results.
Please note: In this plot the frequency scaling factor is not applied.

Table 1. Numerical comparison describing the similarity in the range of 950- 1800 cm^{-1} between the calculated IR and VCD spectra for the **(R)** enantiomer at the cc-pVTZ / B3PW91 w/ CPCM (Chloroform) level and the observed IR and VCD spectra for **MK-26 P2**.

Cal. (950-1800cm^{-1})	Numerical comparison	Observed **MK-26 P2**
**(R)**	scaling factor	0.979
	IR similarity (%)	91.5
	$^{a}\sum$ (%)	69.4786
	$^{b}\Delta$ (%)	59.608
	Confidence Level (%)	98

$^{a}\sum$: single VCD similarity, gives the similarity between the calculated and observed VCD spectra.
$^{b}\Delta$: enatiomeric similarity index, gives the difference between the values of $\sum$ for both enantiomers of a given diastereoisomer.

VCD Absolute Configuration Determination Report

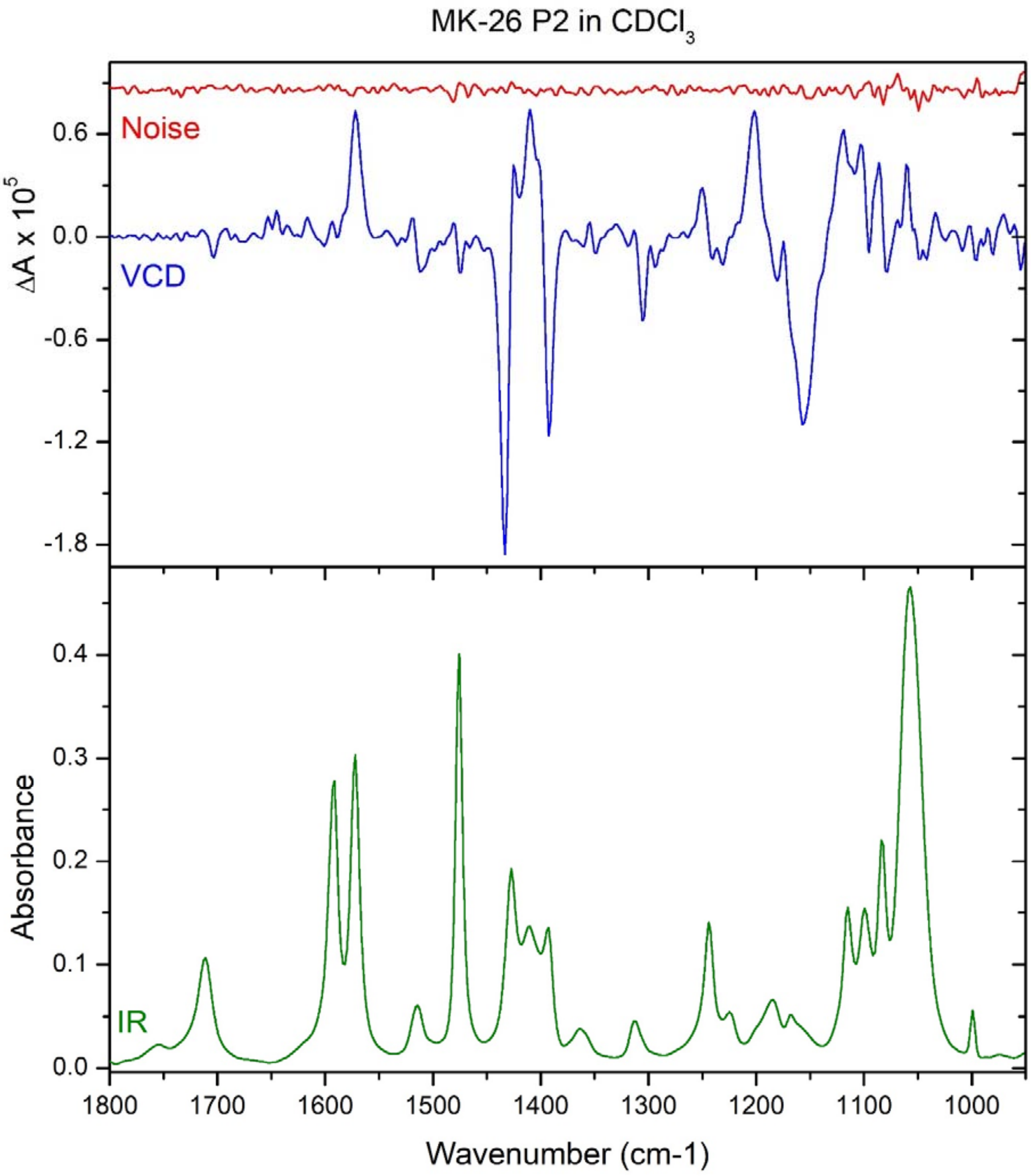

IR (lower frame) and VCD (upper frame) spectra of **MK-26 P2** in $CDCl_3$; 100um path-length cell with BaF_2 windows; 12 h collection for each enantiomer; instrument optimized at 1400 cm^{-1}. Solvent subtracted IR and enantiomer subtracted VCD spectra are shown. Uppermost trace is the VCD noise spectrum.

Title:

VCD Absolute Configuration Determination Report

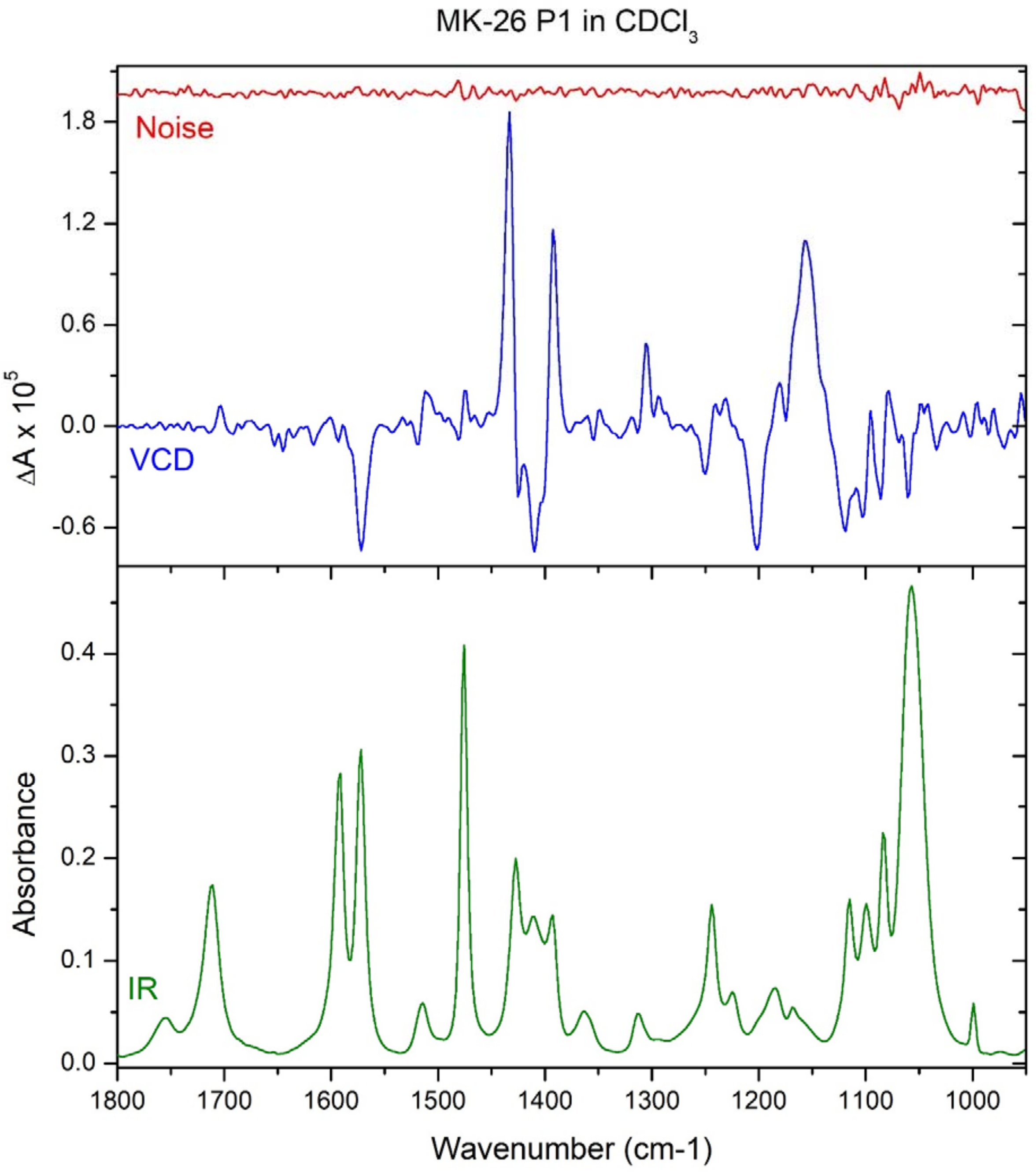

IR (lower frame) and VCD (upper frame) spectra of **MK-26 P1** in $CDCl_3$; 100um path-length cell with BaF_2 windows; 12 h collection for each enantiomer; instrument optimized at 1400 cm^{-1}. Solvent subtracted IR and enantiomer subtracted VCD spectra are shown. Uppermost trace is the VCD noise spectrum.

VCD Absolute Configuration Determination Report

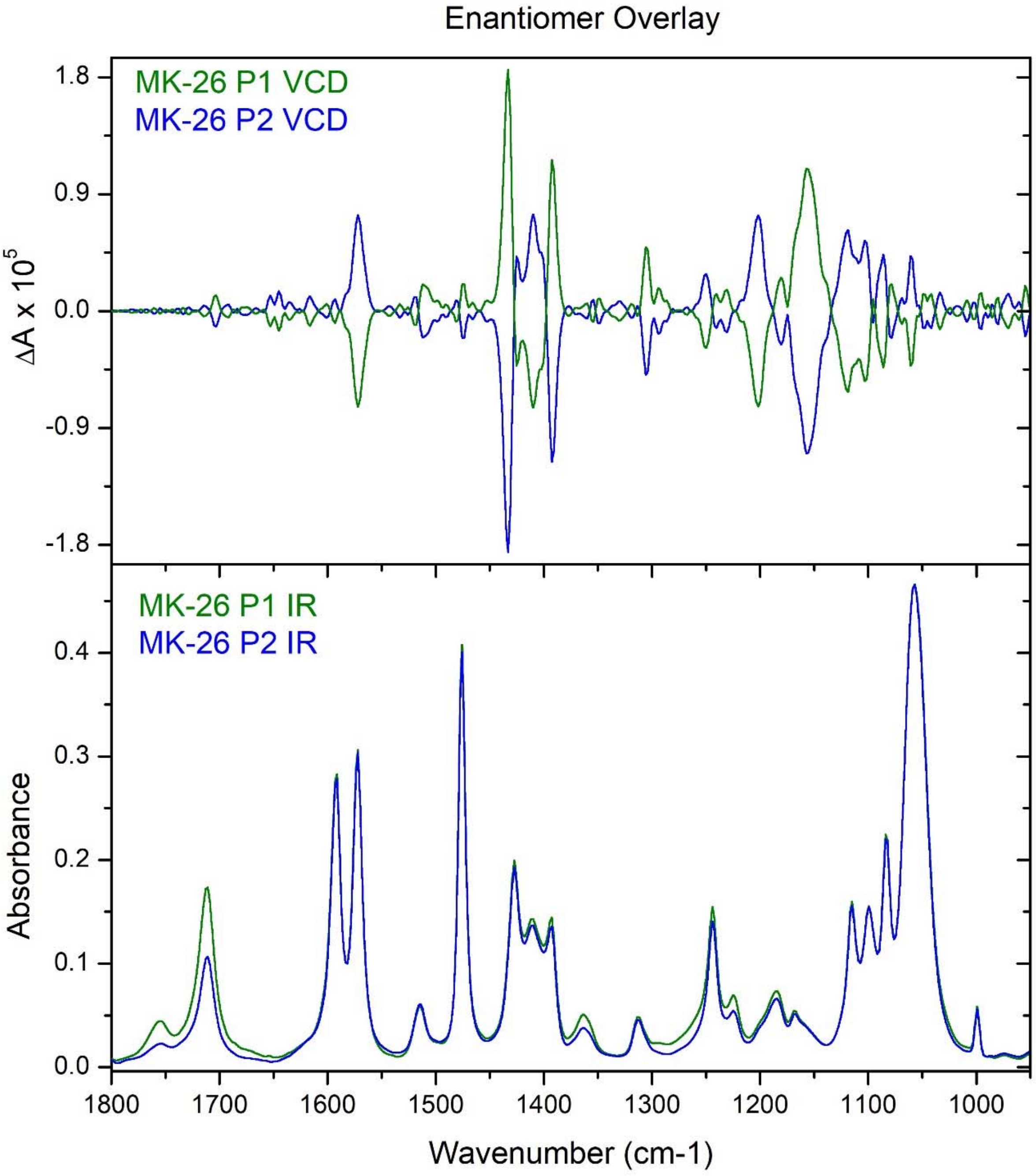

Overlay of both enantiomers, **MK-26 P2** and **MK-26 P1**. The IR are nearly identical as expected. The VCD are mirror images due to the half difference processing (E1 – E2) / 2.

Title:

VCD Absolute Configuration Determination Report

MK-26 P2 Measured vs. Calculated (R)

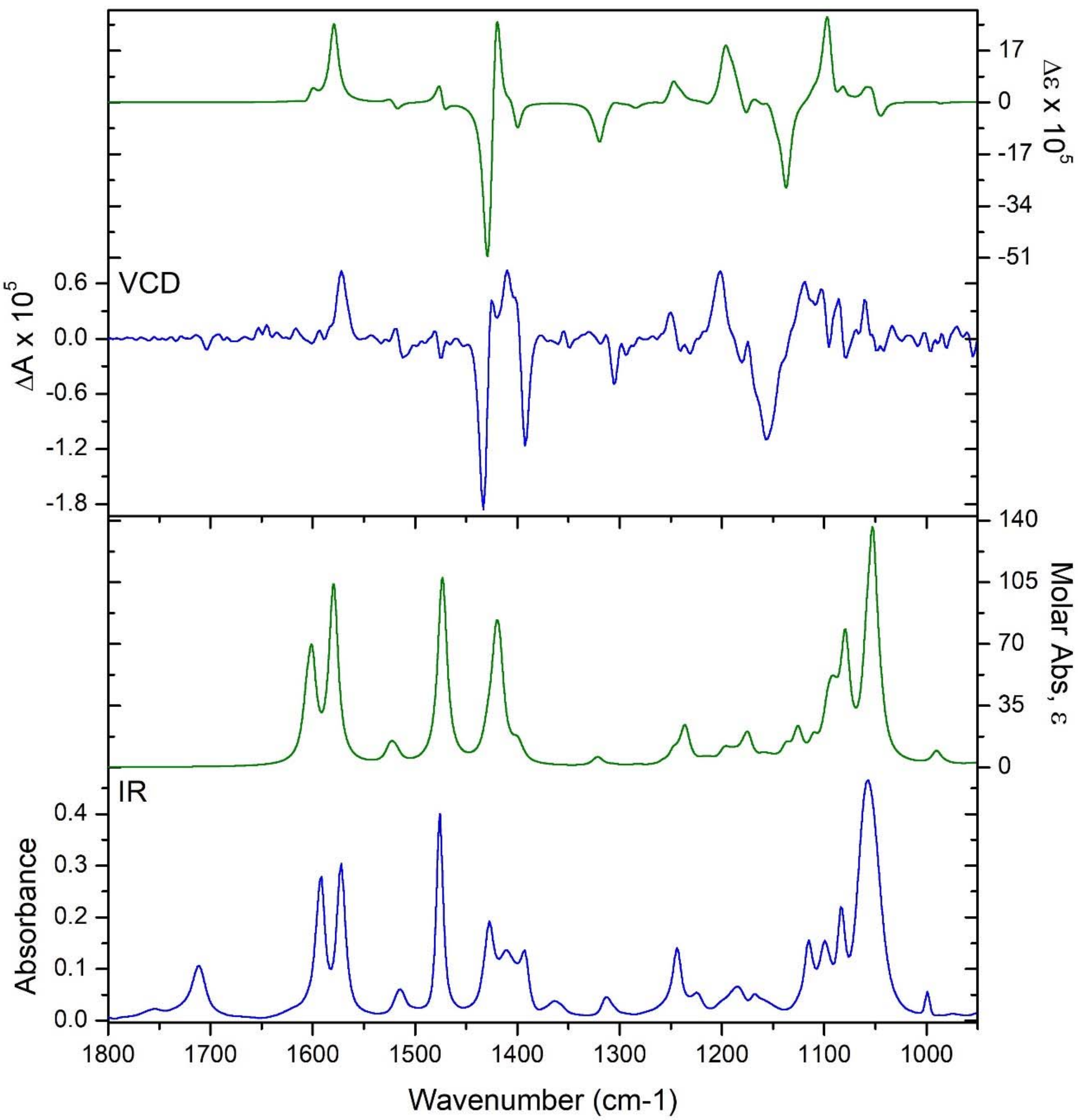

IR (lower frame) and VCD (upper frame) spectra observed for **MK-26 P2** (left axes) compared with Boltzmann-averaged spectra of the calculated conformations for the **(R)** configuration, (right axes).

17546 Bee Line Hwy | Jupiter, Florida 33478, USA
ph. 561 625.0133 | fax. 561 625.0717 | www.biotools.us

Title:

VCD Absolute Configuration Determination Report

Four lowest energy conformers (of 37 from Boltzmann average) - (R) Configuration:

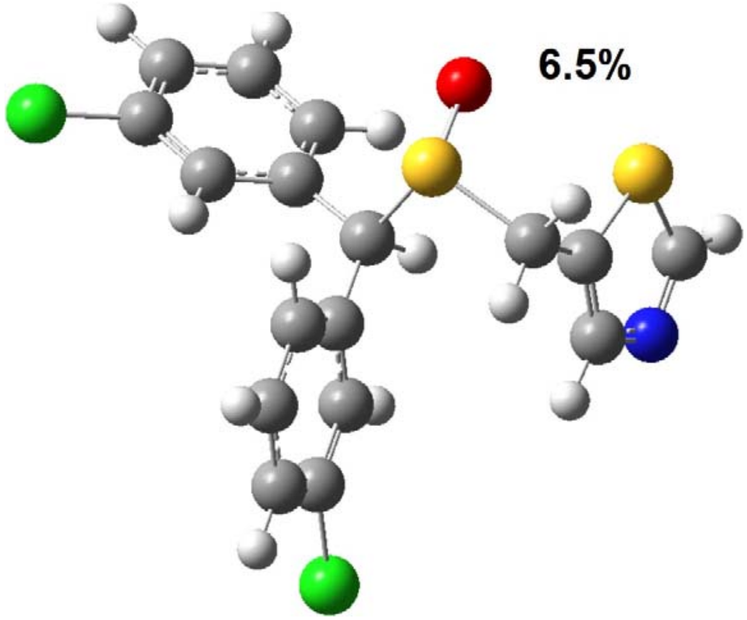

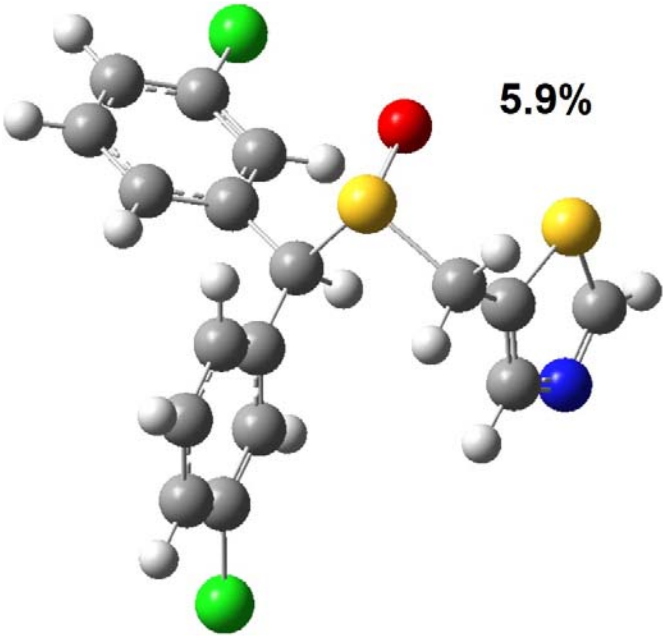

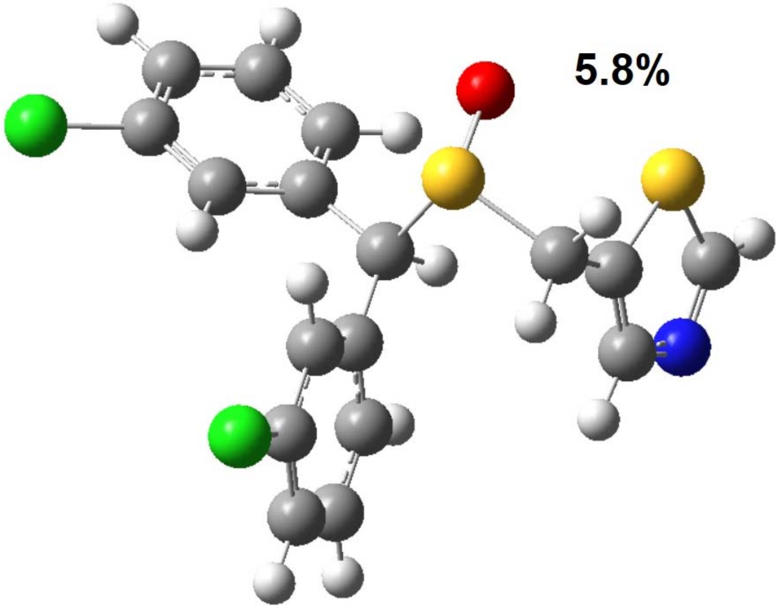

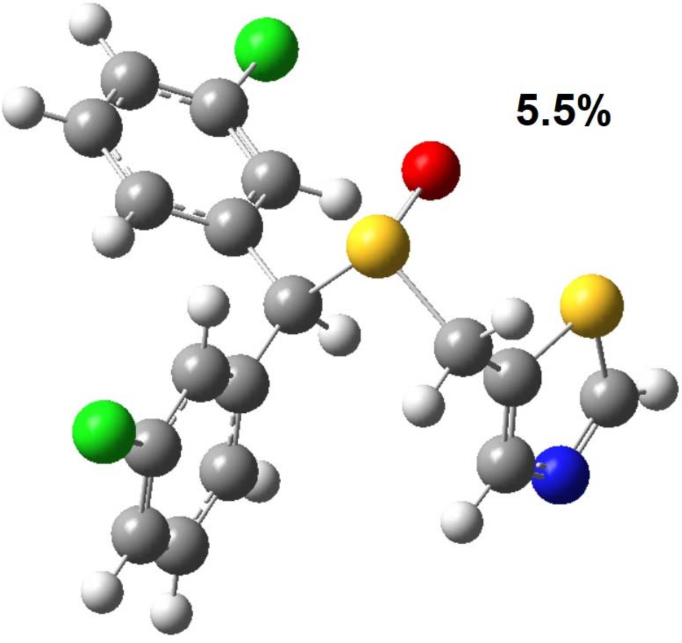

17546 Bee Line Hwy │ Jupiter, Florida 33478, USA
ph. 561 625.0133 │ fax. 561 625.0717 │ www.biotools.us

VCD Absolute Configuration Determination Report

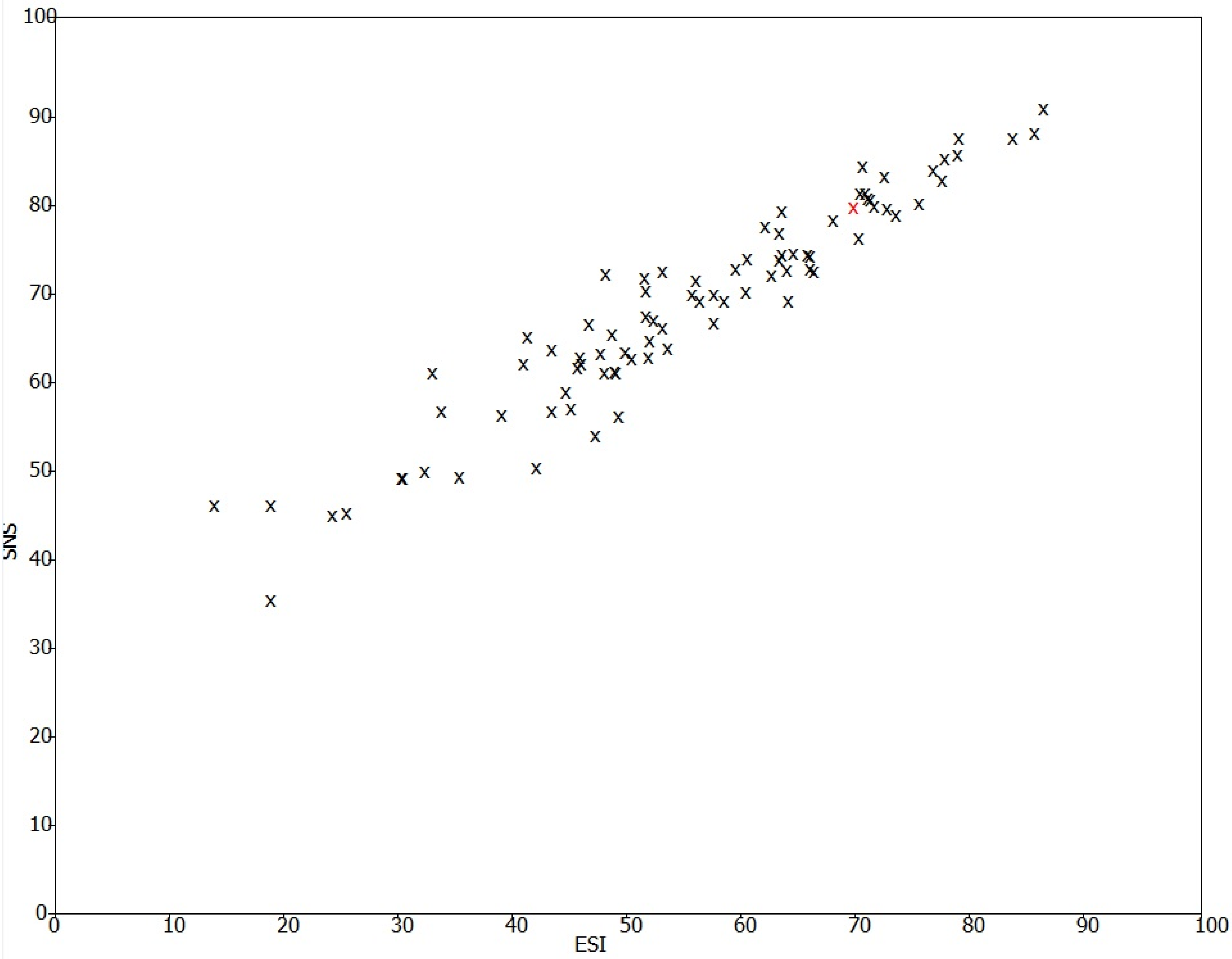

Plot of ESI (similarity of correct enantiomer minus incorrect enantiomer to calculated) vs SNS (overall similarity of correct enantiomer to calculated) for a library of correct assignments verified independently by X-Ray other method (Black X marks). Red X is **MK-26 P2**.

17546 Bee Line Hwy | Jupiter, Florida 33478, USA
ph. 561 625.0133 | fax. 561 625.0717 | www.biotools.us
